# Evolutive emergence and divergence of an Ig regulatory node: An environmental sensor getting cues from the aryl hydrocarbon receptor?

**DOI:** 10.3389/fimmu.2023.996119

**Published:** 2023-02-03

**Authors:** Pietro D'Addabbo, Domenico Frezza, Courtney E.W. Sulentic

**Affiliations:** ^1^ Department of Biology, University of Bari “Aldo Moro”, Bari, Italy; ^2^ Department of Biology E. Calef, University of Rome Tor Vergata, Rome, Italy; ^3^ Department of Pharmacology & Toxicology, Boonshoft School of Medicine, Wright State University, Dayton, OH, United States

**Keywords:** B lymphocytes, AhR (aryl hydrocarbon receptor), dioxin, antibody expression, evolution and regulation of the immunoglobulin heavy chain (IgH), IgH 3′ regulatory region (3′RR), species differences, human polymorphisms

## Abstract

One gene, the immunoglobulin heavy chain (*IgH*) gene, is responsible for the expression of all the different antibody isotypes. Transcriptional regulation of the *IgH* gene is complex and involves several regulatory elements including a large element at the 3’ end of the *IgH* gene locus (3’RR). Animal models have demonstrated an essential role of the 3’RR in the ability of B cells to express high affinity antibodies and to express different antibody classes. Additionally, environmental chemicals such as aryl hydrocarbon receptor (AhR) ligands modulate mouse 3’RR activity that mirrors the effects of these chemicals on antibody production and immunocompetence in mouse models. Although first discovered as a mediator of the toxicity induced by the high affinity ligand 2,3,7,8-tetracholordibenzo-p-dioxin (dioxin), understanding of the AhR has expanded to a physiological role in preserving homeostasis and maintaining immunocompetence. We posit that the AhR also plays a role in human antibody production and that the 3’RR is not only an *IgH* regulatory node but also an environmental sensor receiving signals through intrinsic and extrinsic pathways, including the AhR. This review will 1) highlight the emerging role of the AhR as a key transducer between environmental signals and altered immune function; 2) examine the current state of knowledge regarding *IgH* gene regulation and the role of the AhR in modulation of Ig production; 3) describe the evolution of the *IgH* gene that resulted in species and population differences; and 4) explore the evidence supporting the environmental sensing capacity of the 3’RR and the AhR as a transducer of these cues. This review will also underscore the need for studies focused on human models due to the premise that understanding genetic differences in the human population and the signaling pathways that converge at the 3’RR will provide valuable insight into individual sensitivities to environmental factors and antibody-mediated disease conditions, including emerging infections such as SARS-CoV-2.

## Introduction

Decreased antibody (Ig) production significantly impacts human health by weakening the ability to maintain immunocompetence and survive infectious diseases (1–4). Conversely, antibodies against self-proteins play a major role in autoimmune diseases (5–7). The immunoglobulin heavy chain (*IgH*) gene is responsible for the expression of all Ig classes/isotypes, i.e. IgM, IgD, IgG, IgE, and IgA. These isotypes have different effector functions in mediating immunity (8–11). Therefore, environmental and/or genetic-induced alterations in processes that control *IgH* expression will significantly affect human health. However, the ability to directly determine the human health impact of environmental exposures is challenging due to the limited endpoints that can be evaluated in exposed populations and the difficulty in assessing altered immune function (12). However, epidemiology and clinical studies examining Ig levels as well as responses to vaccines are supportive of an increased risk of altered immunity to common infections following environmental exposures to chemicals and pollutants that induce low or moderate immune suppression (4, 13, 14). Studies have primarily relied on animal models (i.e. mouse and rat) to directly evaluate the immunomodulatory potential of chemicals and the underlying mechanisms (15). Several high-profile incidents of human exposures to 2,3,7,8-tetrachlorodibenzo-p-dioxin (dioxin) and polychlorinated biphenyls (PCB) lead to the discovery of the aryl hydrocarbon receptor (AhR), which binds with varying affinity to a plethora of environmental chemicals and pollutants (16). In animal models, chemicals that bind the AhR are well-established inhibitors of *IgH* expression and Ig levels though the actual mechanistic role of the AhR is less clear (**Table 1**). Additionally, epidemiological studies and *in vitro* studies with human B cells support altered Ig levels following exposure to AhR ligands, though the studies are limited and suggest variation in sensitivity (**Table 1**). Interestingly, it is becoming increasingly evident that there are a number of endogenous, microbial-derived, and dietary AhR ligands and that the AhR is a regulator of various immune functions in response to the environment (70, 71). This suggests a central physiological role of the AhR in preserving homeostasis and maintaining immunocompetence (72–75). Furthermore, the AhR is gaining attraction as a potential therapeutic target in inflammatory conditions with an AhR ligand currently in FDA review to treat psoriasis (76–79). However, in the context of human *IgH* expression and Ig production, the effects of AhR or its mechanistic role is not well defined (**Table 1**). This is further complicated by the fact that our understanding of basic immune function has been largely based on mouse models. This is noteworthy because of the species differences in AhR ligand binding and signaling (73) as well as the significant differences in the *IgH* gene between rodents and humans (80). These differences will likely translate to functional differences. Given the importance of antibodies in defense and disease, this represents a significant knowledge gap in human B-cell function and impact of environmental exposures. This review will highlight the current state of knowledge regarding *IgH* gene regulation and role of the AhR in modulation of Ig production, the significant species and population differences in the *IgH* gene, and the evidence supporting the environmental sensing capacity of the 3′RR regulatory node with AhR signaling as a key transducer between environmental stressors and altered immune function. This review will also underscore the need for studies focused on human models due to the premise that understanding genetic differences in the human population and the signaling pathways that converge at the 3′RR will provide valuable insight into individual sensitivities to environmental factors and antibody-mediated disease conditions.

**Table 1:** Effects of dioxin or dioxin-like chemicals on antibody production and role of the AhR.

Exposure to Dioxin or Dioxin-like Chemicals^1^			
Endpoint Measured	Rodent		Human
**μ heavy chain, light chain, and J chain expression**	***Inhibition*:** •Splenocytes (79) •B-cell line (248)* (17–19) •B-cell line, PCB exposure (20)^		***No effect:*** •Primary B cells (inhibition of secretion rather than gene expression) (17)
**Antibody-forming cell (AFC) response**	***Inhibition:*** •*In vivo* exposure (21)* (22, 23)^ (24–26) •*In vivo* PCB exposure (22)^ ***No effect:*** •IgG or IgA AFC in mediastinal lymph node following *in vivo* exposure in mice infected with influenza virus (27)		***Inhibition:*** •Primary B cells, B[a]P exposure (28)
**IgM**	***Inhibition:*** •Serum antigen-specific (22)^ •Plasma and BALF IgM following *in vivo* exposure in mice infected with influenza virus (27), •Primary B cells (29) •Interstrain differences in sensitivity of primary B cells (30)* •Splenocytes (17, 31) •B-cell line (32–34)* (18) •Rat intracellular IgM^+^ primary B cells; *in vivo* exposure then *in vitro* cellular stimulation (35)* •Serum antigen-specific IgM, PCB exposure (22)^ •B-cell line, PCB exposure (20)^ •Primary B cells, ITE exposure (36)* ***No effect:*** * Spleen and gut B2 cells; peritoneal B1 (CD5^+^) cells; *in vivo* exposure then *in vitro* cellular stimulation (37)		Evidence for Responders and non-responders (donor specific and B-cell type) ***Inhibition:*** • Primary B cells (17, 30, 38–40) • CD5^+^ innate-like primary B cells with high LCK (41, 42) • B-cell line (43)* • Serum (PCB contaminated rice bran oil in Taiwan and Japan) (44, 45) ***Increase:*** • Primary B cells (30, 39, 40) ***No effect:*** • Serum (Industrial Accident in Seveso, Italy) (46) • Primary B cells (30, 39, 40) • CD5^-^ primary B cells (41, 42) • Primary B cells from atopic patients and control subjects (47)
**IgG**	***Inhibition:*** •Serum antigen-specific IgG (22) •MOG-specific IgG in serum, spleen, spinal cord in experimental autoimmune encephalomyelitis model (48) •Total IgG1 and IgG3 secretion from primary B cells and splenocytes (49) •Plasma and BALF influenza specific IgG2a, IgG2b, IgG1 (27) •Serum total IgG1 (50) •% IgG3 primary splenic B cells after *in vitro* stimulation (51)* •IgG1 and mRNA for secreted IgG1 from primary B cells, ITE exposure (36)* ***Increase:*** •Serum total IgG1 and OVA-specific IgG1 in oral tolerance model (50) ***No effect:*** •mRNA for germline and membrane-bound IgG1 in primary B cells, ITE exposure (36)		***Inhibition:*** • Primary B cells (17) • Plasma IgG1 (Vietnam War Korean Veterans exposed to Agent Orange (52) • Serum total IgG (Industrial Accident in Seveso, Italy) (46) ***Increase:*** • Serum (PCB contaminated rice bran oil in Japan) (45) ***No effect:*** • All IgG subtypes, primary B cells from atopic patients and control subjects (47) • CD5^+^ innate-like and CD5^-^ primary B cells (42)
**IgA**	***Inhibition:*** •Fecal (53)* •Fecal in pups (male pups more sensitive than female) from TCDD-exposed dams due to impaired chemotaxis of B1 cells to gut (37) * % IgA primary splenic B cells after *in vitro* stimulation (51)* ***Increase:*** •Fecal (males more sensitive than females) (54) •Fecal, TCDF exposure (55) •Fecal and colon in colitis model (56)* •Fecal OVA-specific IgA in oral tolerance model (50) •Plasma influenza-specific (27) •B-cell line (33, 57)* ***No effect:*** •IgA AFC in mediastinal lymph node (27)		***Inhibition:*** • Serum (PCB contaminated rice bran oil in Taiwan and Japan) (44, 45) ***No effect:*** • Serum (Industrial Accident in Seveso, Italy) (46) • Both IgA subtypes, primary B cells from atopic patients and control subjects (47)
**IgE**	***Inhibition:*** •IgE and mRNA for secreted IgE from primary B cells, ITE exposure (36)* ***No effect:*** •mRNA for germline and membrane-bound IgE from primary B cells, ITE exposure (36)		***Increase:*** • Primary B cells from atopic patients but not control subjects (47) • Primary B cells but dependent on time of exposure to PAH-DEP after cellular stimulation (increase when PAH-DEP exposure was 2 to 5 days after stimulation) (58)
**Vaccine titers** **in exposed human cohorts**	Perinatal PCB exposure (Faroe Islands) associated with decreased Ig titers against Tetanus and Diphtheria vaccines (59, 60) Perinatal dioxin and dioxin- and non-dioxin-like PCB exposure (Norway) associated with decreased Ig titers against measles vaccine but not rubella, tetanus or influenza vaccines (61) Perinatal PCB-153^#^ exposure (eastern Slovakia) correlated with decreased antigen-specific IgA and IgG titers against tuberculosis vaccine (62)		
Endogenous role of AhR on antibody production			
Rodent		Human	
***AhR KO Mouse:*** •Increased antigen-induced total number of antibody-forming cells per spleen (21) •Increased serum OVA-specific IgG1 and IgE and total IgE but not total IgG1 (63) •Increased *aicda* mRNA and AID protein with B-cell stimulation and increased % IgA^+^ and IgG3^+^ cells (51) ***AhR KO Rat or AhRA treatment:*** •Increased stimulation-induced intracellular IgM^+^ B cells (35) ***Primary mouse splenocytes and B cells:*** •Cellular stimulation increased AhR levels and basal or ligand-induced activation (i.e. CYP induction and DRE binding, respectively) (64, 65) ***Mouse B-cell line:*** •Cellular stimulation increased AhR levels (32)		***Primary B cells and Burkitt’s lymphoma B-cell line:*** •Cellular stimulation increased AhR levels and basal or ligand-induced AhR activation (i.e. CYP induction) (66–68) ***CD5^+^ innate-like primary B cells:*** •Increased basal AhR levels and ligand-induced AhR activation compared to CD5^-^ B cells (42)	

^1^Effects listed are following dioxin exposure unless otherwise noted. *AhR-dependent effect; ^ indirect evidence for AhR-dependent effect; # non-dioxin-like PCB that has been shown to antagonize AhR activation and induction of CYP1A1 (20, 69). B[a]P, benzo[a]pyrene; PAH-DEP, polyaromatic hydrocarbons-diesel exhaust particles; TCDF, 2,3,7,8-tetrachloro-dibenzofuran; AhRA, AhR antagonist. References are indicated in parathesis.

## The aryl hydrocarbon receptor signaling pathway: More than a protective mechanism against environmental chemicals

**Figure 1:**
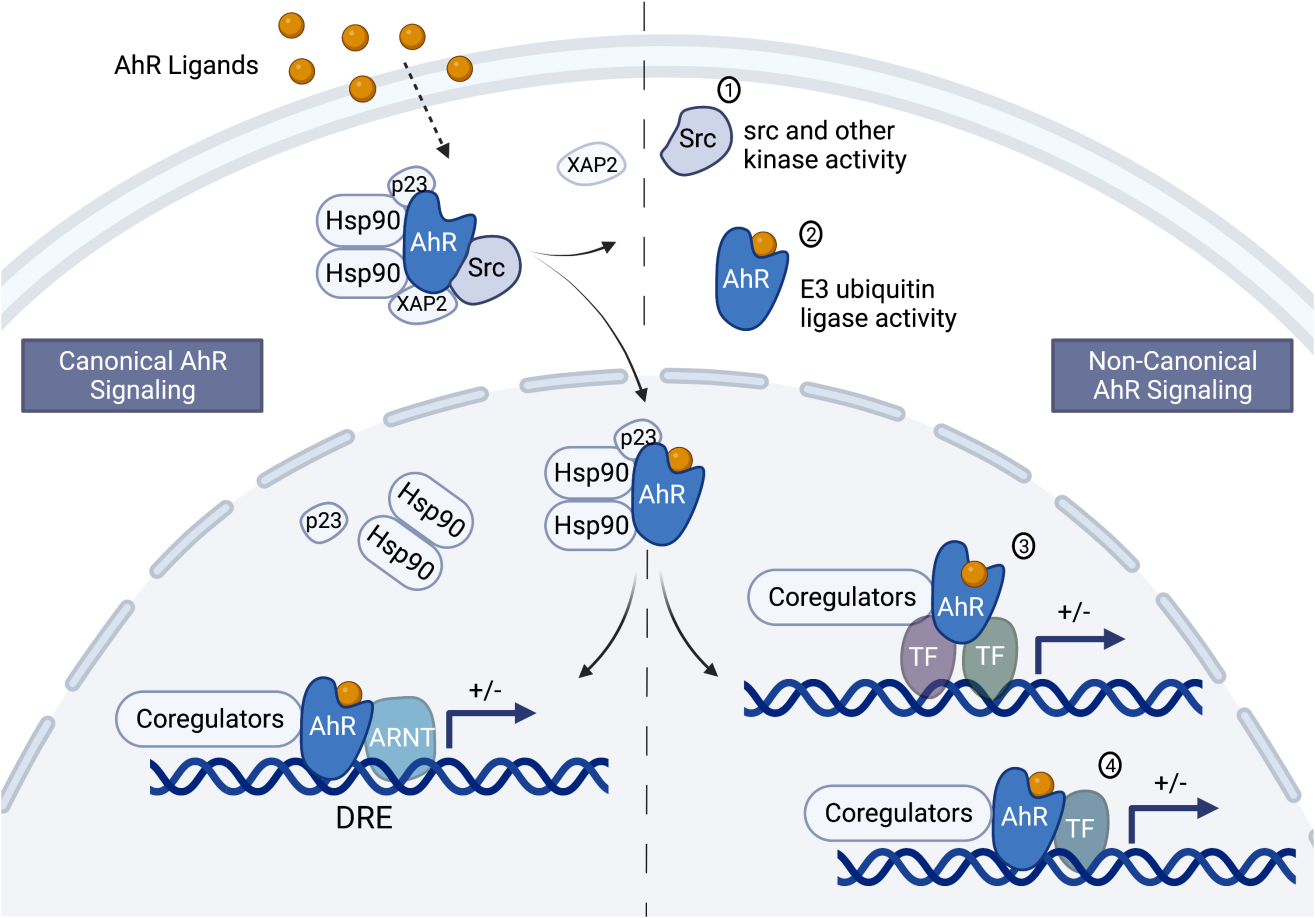
Schematic of the canonical and non-canonical aryl hydrocarbon receptor signaling pathways. Depending on the ligand and perhaps other unknown factors, aryl hydrocarbon receptor (AhR) signaling could occur through the canonical and/or the non-canonical pathway. In the cytosol, the AhR is complexed with Src kinase and several chaperone proteins, including the 90 kDa heat shock protein (hsp90), p23 and XAP2 (or AhR-interacting protein, AIP). The chaperone proteins protect the AhR from ubiquitination and keep it in a conformation that can bind ligand. Ligand binding causes a conformational change resulting in the disassociation of XAP2 and Src, exposure of the AhR nuclear localization signal, and AhR translocation into the nucleus. Once in the nucleus the other chaperone proteins dissociate from the AhR leading to canonical or non-canonical signaling. Canonical signaling (left side of the center, dashed line) involves dimerization of the AhR and the AhR nuclear translocator (ARNT) and binding to dioxin response elements in sensitive genes. The AhR/ARNT nuclear complex interacts with coregulators to either positively or negatively (indicated by the “+/- “) modulate gene transcription (bent arrow indicates the transcription start site). Non-canonical signaling (right side of the dashed center line) involves genomic and non-genomic signaling. The non-genomic signaling could be mediated through the release of Src or interactions with other kinases (1) and the E3 ubiquitin ligase activity of the AhR (2). The genomic signaling could be indirect (3) or direct (4) through interactions with transcription factors other than ARNT, such as NFκB/Rel proteins, AP-1, POU, NF-1, SP-1, and KLF-6. Figure was created with BioRender.com.

The AhR signaling pathway was first characterized in the upregulation of Phase I and II metabolic enzymes upon exposure to halogenated aromatic hydrocarbons (HAH), such as 2,3,7,8-tetrachlorodibenzo-p-dioxin (TCDD or dioxin). The AhR is a ligand-activated nuclear receptor that when activated by ligand translocates from the cytosol to the nucleus where it binds the AhR nuclear translocator (ARNT). The AhR/ARNT transcription factor complex binds to dioxin response elements (DRE) and through interactions between the AhR transactivation domain and co-activators or co-repressors modulates transcription of sensitive genes (81) (**Figure 1**, left side of dashed center line). A variety of environmental chemicals are ligands for the AhR, which first established AhR signaling as a protective pathway in clearing chemicals from the body through metabolism or conjugation to a more water-soluble chemical for excretion and elimination (82, 83). However, increasingly, studies are pointing to physiological roles of the AhR in a variety of tissues and cell types, including the immune system (84–87). Although endogenous ligands such as lipoxin A4 and tryptophan metabolites have been identified to bind the AhR, no clear physiological role has been attributed to both the AhR and the endogenous ligands (70, 88). Furthermore, AhR knockout studies in mice and human cell lines support differing biological effects on inflammatory responses and cytokine levels mediated by the AhR in the absence versus the presence of exogenous ligands (89–98).

The AhR signaling pathway appears to have evolved to include metabolizing/detoxifying activities. Despite conservation of the AhR through evolution, the *C. elegans (platelmint)* AhR homolog does not bind dioxins and has low amino acid identity with mouse and human in the ligand binding domain, suggesting either binding to unknown endogenous ligands or ligand-independent effects (99–102). This also suggests origins of a physiological function for the AhR distinct from its metabolizing/detoxifying activity (99–101).

In terms of ligand binding, the AhR, much like the estrogen receptor (ER), which is also a nuclear receptor, can bind a wide range of ligands. An increasing number of chemicals in the environment, including natural and industrial chemicals, as well as dietary and pharmaceutical chemicals have been shown to bind the AhR with varying affinity (71, 103). Interspecies and intraspecies differences in ligand affinity have also been found (**Table 2**). For instance, dioxin binds the human AhR with ~10-fold lower affinity than the AhR from a dioxin-sensitive mouse strain, which appears to be due to a single amino acid difference (106, 107). Additionally, the transactivation domain of the human and mouse AhR is only 58% homologous, which may result in interactions with different co-activators/co-repressors and perhaps explain the differential effects on genes modulated in human versus mouse following exposure to dioxin (73, 109–111). Furthermore, a humanized AhR mouse model exhibited different dioxin-induced biological effects as compared to a wildtype mouse, which could not be explained by differences in AhR binding affinity for dioxin (112). Taken together these results suggest significant species differences in AhR ligand binding and signaling through the C-terminal transactivation domain.

**Table 2:** Species differences in AhR ligand specificity and activation of the AhR.

Species	Ligand potency in activating the AhR	References
*Caenorhabditis elegans*	No ligand binding	(99–102)
*Xenopus laevis*	Dioxin < FICZ	(104)
Mouse	Dioxin ≥ FICZ	(104, 105)
Human	Dioxin ≤ FICZ < Indole, Indirubin	(71, 72, 105)
Ligand	Species differences in ligand potency	References
TCDD	Mouse > Human	(106, 107)
FICZ	Rat > Human	(108)
Indole	Human specific	(72)
Indirubin	Human > Mouse	(71, 105)

While the human AhR has lower affinity for dioxin as compared to the mouse AhR, the reverse is true for some AhR ligands. Indirubin, which is naturally produced by plants and bacteria, binds the human AhR with much higher affinity than the mouse AhR (71, 105). Indirubin has been detected in human urine at concentrations that induce AhR activity, indicating human exposure to biologically relevant concentrations of AhR ligands (113). Additionally, indole, a bacterial metabolite of tryptophan, is a human specific AhR ligand that does not bind the mouse AhR (72). In contrast to indole, the tryptophan-derived AhR ligand 6-formylindolo[3,2-*b*]carbazole (FICZ) is more potent at inducing AhR activity in a rat hepatic cell line as compared to one from human (108). When comparing the potency of FICZ versus TCDD in different species, FICZ was far more potent than dioxin in a *Xenopus laevis* cell line (104), while dioxin demonstrated more potency than FICZ in mouse hepatic cell lines (105). Whereas, in human hepatic cell lines, dioxin and FICZ were equipotent (105). These studies support the existence of multiple species-specific differences in the AhR signaling pathway (**Table 2**), thus leading to uncertainty in translating the findings from murine studies to the physiological and ligand-mediated effects of the AhR in humans.

The number of AhR ligands from dietary sources such as indirubin and polyphenols as well as bacterial AhR ligands such as indole and tryptophan metabolites suggest a modulatory role of the AhR in reacting/adapting to the environment (96, 105, 108, 113–115). Additionally, due to the number of bacterial metabolites that are AhR ligands, the AhR has been suggested to play a role in mucosal immunity as a sensor to control commensal bacteria (72, 84). *C. elegans* use bacteria as a food source and its primitive immune responses, termed “effector-triggered” immunity or surveillance immunity, are mainly directed against intrinsic bacterial attacks (116, 117). Perhaps the AhR evolved from a common ancestral gene (as represented by the ligand independent AhR in *C. elegans*) to bind bacterial ligands and efficiently fine tune a more complex immune response to commensal and pathogenic bacteria. There is a growing body of literature supporting a role of the AhR in most aspects of the immune system (78, 84, 118, 119). This review will focus on examining the role of the AhR in Ig production and the regulation of the *IgH* gene.

## Humoral immunity in defense and disease

Igs are only produced by B lymphocytes and are critical mediators of the humoral immune response. They circulate through the blood, lymph, mucosa, tissue fluids and secretions to protect against extracellular antigens or non-self molecules such as bacteria, parasites, and foreign macromolecules. The basic Ig unit consists of two identical ‘heavy chains’ and two identical ‘light chains’ linked together by disulfide bonds. Each heavy and light chain has a variable (V) region and a constant (C) region. The V regions encode for the antigen-binding pocket and have high sequence variability, whereas the C regions have very little sequence variability. The C region of the heavy chain portion of the Ig defines the five Ig classes (i.e. IgM, IgD, IgG, IgE, IgA) and consequently determines the antibody properties and effector functions. The main function of antibodies is to bind or opsonize (i.e. coat) the antigen to either neutralize it by preventing it from binding its cellular target, or to flag it for destruction and/or clearance by soluble immune mediators (e.g. complement proteins) and other immune cells (e.g. phagocytes) (120).

**Figure 2:**
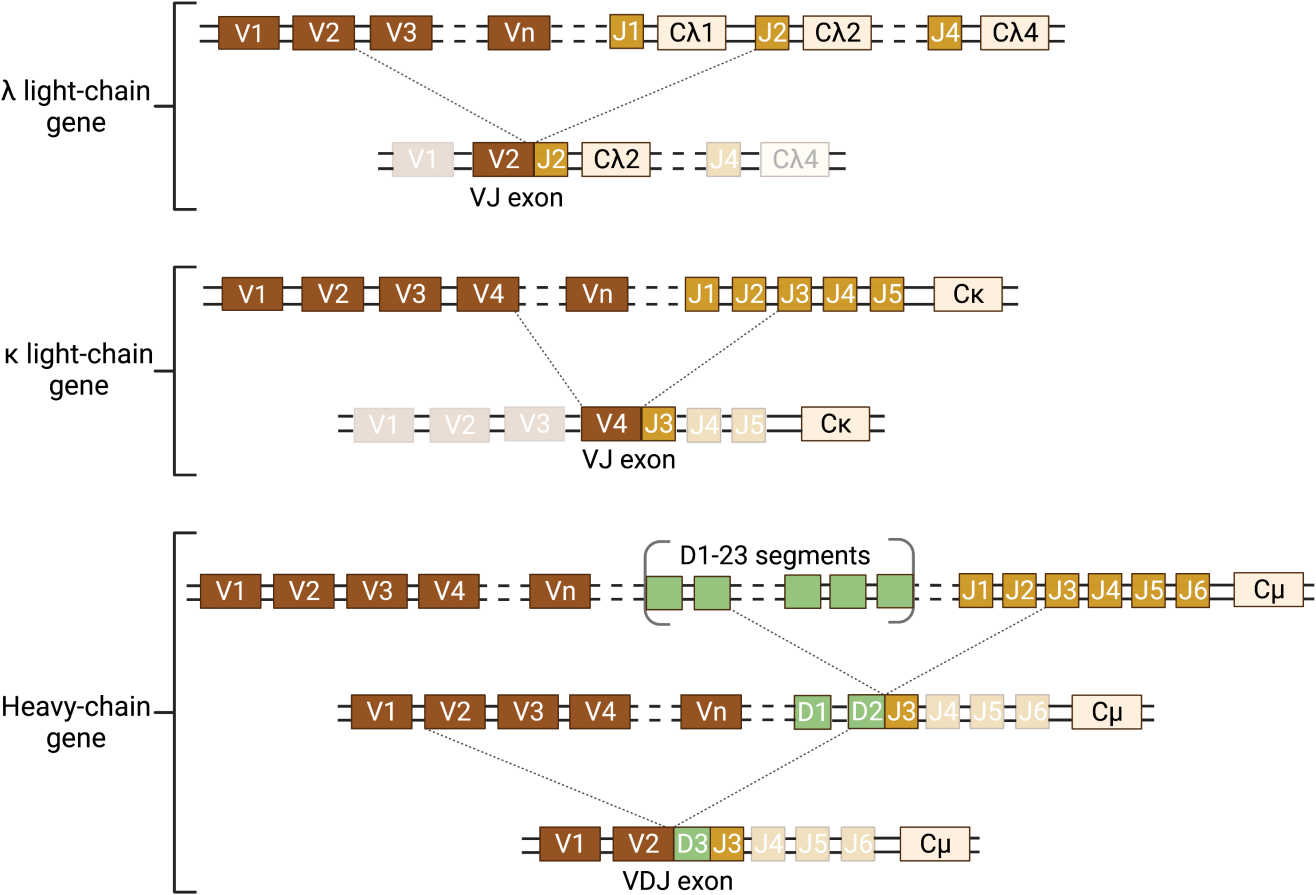
Schematic of somatic recombination of the Ig heavy and light chain genes. The Ig genes are inherited in a fragmented, non-functional state and require somatic recombination during B-lymphocyte development to produce Ig protein. There are two light chain genes (λ and κ) and one heavy chain gene and two alleles of each gene. Only one allele of the light chain and heavy chain gene can be functionally expressed in each B lymphocyte. Expression requires DNA recombination to select one variable (V) and one joining (J) segment for the light chain gene and one V, one diversity (D), and one J segment for the heavy chain gene. The recombined VJ (light chain) and VDJ (heavy chain) exons form the antigen binding pocket of the Ig. This random recombination of V(D)J segments allows for the incredible diversity in the antigen binding pocket of Igs. In humans, there are 34-38 V segments, five J segments and one constant (C) segment for the κ light chain; 29-33 V, 4-5 J, and 4-5 C segments for the λ light chain; and 38-46 V, 23 D, 6 J, and 9 C segments for the heavy chain. The schematic illustrates a hypothetical somatic recombination of one allele for each of the λ and κ light chains and the heavy chain. However, each B lymphocyte will only successfully recombine one light chain and one heavy chain allele. Recombination of the light chain genes requires one recombination of V to J; whereas the heavy chain requires two recombination events, first D to J and then V to DJ. Dotted lines represent the location of the double strand cut (top) and the recombined segments forming the coding exon (bottom joining of lines). Transcription is initiated at the 5′ end of the recombined exon and terminated at the 3′ end of the C exon. Figure was created with BioRender.com.

Antigen recognition by antibodies is highly specific and the immune system has the capacity to produce different Igs specific for different antigens, which is remarkable considering that only two genes, the heavy and light chain Ig genes, encode for antibodies. This diversity is possible due to a process called random or somatic V(D)J recombination, which only occurs in developing B lymphocytes and produces an incredible amount of diversity starting from just a single pair of alleles for both the heavy chain gene and the two light chain genes (i.e. λ and κ) in each individual genome (**Figure 2**).

**Figure 3:**
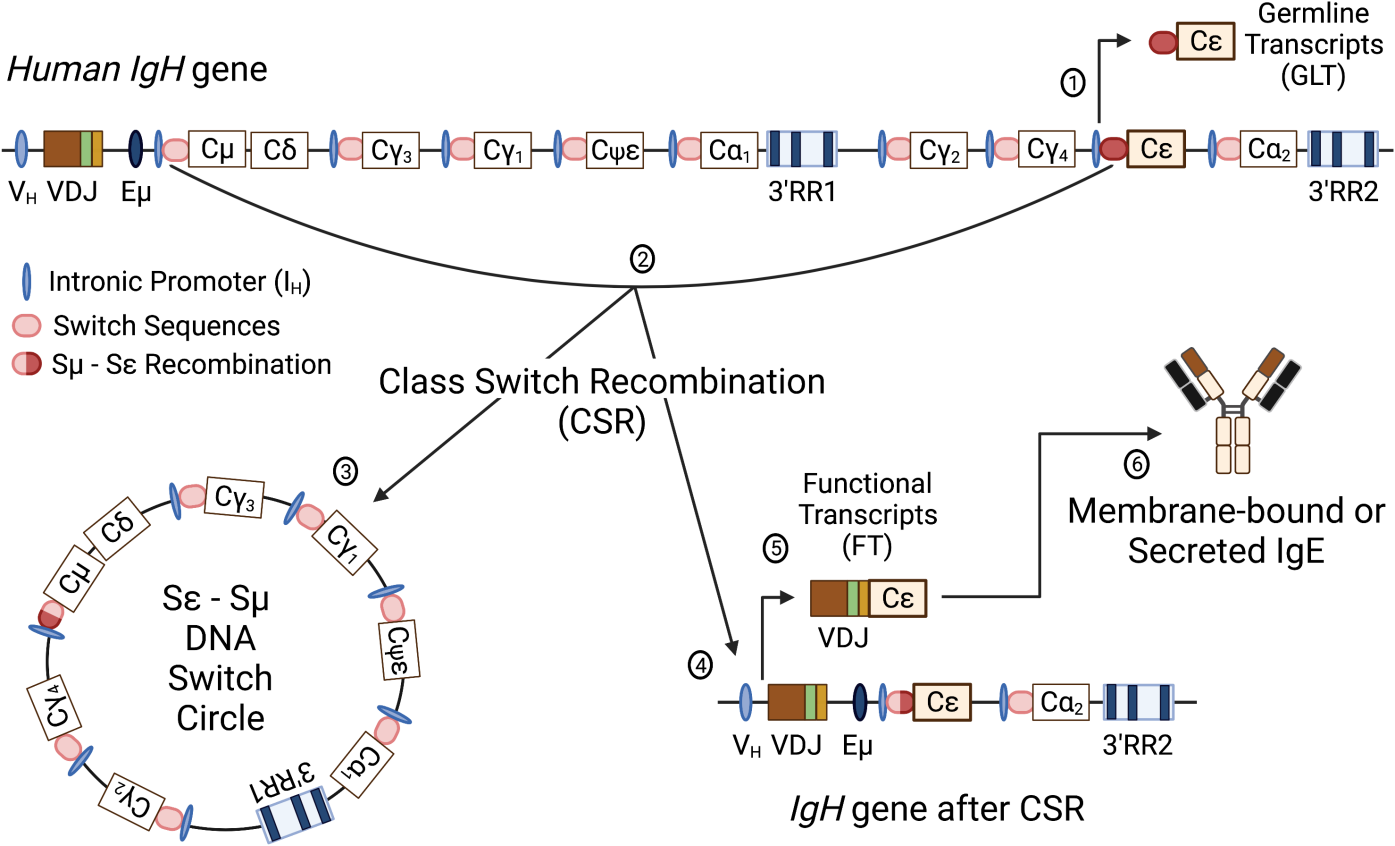
Schematic of the human *IgH* gene locus and class switch recombination. The human recombined *IgH* gene contains a variable heavy chain promoter (V_H_), variable region (recombined VDJ), intronic enhancer (Eμ), constant regions for all antibody isotypes (Cμ, Cδ, Cγ_1-4_, Cε, Cα_1,2_; Cψε is a nonfunctional pseudogene) and two 3’*IgH* regulatory regions (3’RR1 and 3’RR2). Immediately upstream of each C_H_, except δ, is an intronic promoter (I_H_) and a cytidine-rich switch sequence (S_H_). Following successful V(D)J recombination, the first antibody isotypes expressed are IgM and IgD. Class switch recombination (CSR) involves an irreversible DNA recombination of the *IgH* gene that allows a different constant region to be expressed without changing the antigen recognition. Switch to another isotype is dependent on specific cellular stimuli that targets a specific intronic promoter and induces transcription through the switch region of the targeted constant region. The schematic illustrates CSR to Cε to produce IgE. Transcription initiated through the Iε produces nonfunctional germline transcripts and opens the chromatin for targeting by activation-induced cytidine deaminase (AID) (1). AID targets the switch sequences upstream of Cµ and the constant region Cε, causing nonhomologous recombination between the switch regions (i.e. Sµ and Sε) (2). CSR results in removal of the intervening DNA between the recombined switch sequences (Sε-Sµ DNA Switch Circle) (3) and a recombined *IgH* gene with VDJ linked to Cε (4). Transcription of the *IgH* gene results in functional VDJ-Cε transcripts (5) that will be translated into the ε heavy chain protein to form IgE antibodies (6). Figure was created with BioRender.com.

Initial activation of B lymphocytes by a particular antigen results in production and secretion of IgM, which is pentameric. Since IgM affinity for antigen is generally low during a primary or initial immune response, a pentameric structure with 10 antigen binding pockets enhances the ability of IgM to interact with the antigen (120). As activation of the humoral response progresses, antigen-activated B lymphocytes undergo somatic hypermutation and class switch recombination. Somatic hypermutation (SHM) is a process that induces random point mutations in the antigen binding pocket with the objective of increasing Ig affinity for the initial antigen, which may or may not happen. Those B cells with mutations that do increase the affinity for antigen will be preferentially activated by the antigen due to their increased affinity, a process called affinity maturation. Class switch recombination (CSR) induces a DNA recombination event that changes the Ig isotype from the large pentameric IgM to a smaller monomeric (IgG, IgE, IgA) or dimeric (IgA) Ig isotype (**Figure 3**). These processes allow for antigen-specific, high affinity antibodies that are smaller and have different effector functions.

Because of the central role of Ig in humoral immunity, any impairment in Ig production can lead to increased infections and higher morbidity (1–3). Primary immunodeficiency diseases (PID) that are mediated by antibody deficiencies are clinically relevant (121). PIDs were considered rare diseases but are more common than originally thought with an estimated prevalence of approximately 1 in 1,200 persons in the US with approximately greater than 80% considered to be due to an antibody deficiency (122). The prevalence in the European Union appears to range from 0.8 to 18.8 in 100,000 (123). The most common PIDs are selective IgA deficiency and common variable immunodeficiency disease, which presents with low serum IgG, IgA, IgM and poor to absent specific antibody production (122–124). Selective deficiencies in IgM as well as the inability to make IgG antibodies against specific pathogens (i.e. specific antibody deficiency) have been identified and are clinically relevant PIDs. These diseases have been associated with increased susceptibility to recurrent infections as well as autoimmune and allergic diseases and malignancies due to disrupted immune regulation (2, 122, 125, 126). Although several gene mutations (either gain of function or loss of function) have been associated with PIDs, the genetic component for common variable immunodeficiency disease and other selective Ig deficiencies is unknown (127). A combination of genetic, environmental, and epigenetic factors likely contributes to these Ig deficiencies. We propose that exposures to AhR ligands and genetic differences within the 3′RR of the *IgH* gene are contributing factors. We will first review studies supporting altered Ig production by chemicals that have been shown to bind the AhR.

### AhR and Ig production

Dioxin markedly inhibits Ig expression and secretion in an AhR-dependent manner in both *in vivo* and *in vitro* rodent models (**Table 1**) (21, 31–33, 35, 128). Similarly, studies evaluating human primary lymphocytes have generally identified an inhibitory effect of AhR ligands including dioxin and benzo[a]pyrene on plasma cell differentiation and IgM secretion (**Table 1**) (28, 30, 38–42). However, sensitivity of IgM secretion to dioxin varied in B lymphocytes from different human donors. Though the majority tended toward inhibition of IgM secretion, some exhibited no effect and others an increase in IgM secretion (30, 39, 40). Additionally, the inhibitory effect of dioxin on IgM secretion was correlated with a specific B-lymphocyte subtype: CD5^+^ B cells expressing a high level of lymphocyte-specific protein tyrosine kinase (LCK) (17, 41, 42). CD5^+^ B cells represent a more innate-like B-cell population (129). Further mechanistic analysis identified an inhibition of Ig secretory processes rather than altered Ig gene expression in the inhibition of IgM secretion from human CD5^+^ cells exposed to dioxin (17). Additionally, dioxin was shown to increase LCK expression in an AhR-dependent manner, which appears to play a role in the Ig secretory process (41).

These results with high LCK-expressing CD5^+^ B cells contrast with mouse studies, which support decreased Ig secretion due to an AhR-mediated transcriptional inhibition of Ig genes (**Table 1**). However, other studies evaluating the effect of AhR ligands on human Ig production suggest other mechanistic pathways besides the inhibition of secretory processes seen in high LCK-expressing CD5^+^ B cells. This may indicate interplay between the AhR and different cellular signaling pathways at different stages of B-lymphocyte maturation and function. For instance, dioxin increased spontaneous IgE secretion in B lymphocytes isolated from patients with atopic dermatitis that was not due to effects on B-lymphocyte proliferation and appeared to be limited to post-switched IgE^+^ B-cells (47). A similar increase in IgE was demonstrated when human B lymphocytes or peripheral blood mononuclear cells were first stimulated to induce a CSR to IgE and then treated with dioxin or an extract of polyaromatic hydrocarbons from diesel exhaust particles, which include AhR ligands (58). Epidemiological studies also support differential Ig responses associated with exposure to dioxin or polychlorinated biphenyls (PCBs) (**Table 1**). Evaluation of Korean Veterans (Vietnam War) suspected of being exposed to the dioxin-contaminated herbicide, Agent Orange, demonstrated an increase in plasma IgE and a decrease in plasma IgG1 (52). Additionally, a correlation between increased dioxin plasma concentrations and decreased IgG levels was revealed in a population exposed to dioxin in Seveso, Italy (46). Furthermore, studies evaluating vaccine-responsiveness in children suggest a clinically relevant decrease in Ig titers (i.e. concentrations) due to perinatal exposure to dioxins or PCBs, many of which are AhR agonists (**Table 1**). However, there were differences in the response, which may relate to differences in toxicant exposure. Higher concentrations of PCBs associated with decreased Ig titers against tetanus and diphtheria that correlated with higher concentrations of PCBs in a Faroe Islands cohort (59, 60). Whereas evaluation of a Norway cohort identified an association between maternal dietary exposure to PCBs and dioxins with decreased specific Ig titers to the measles vaccine but not to the rubella, tetanus or influenzae type b vaccines (61). Additionally, perinatal exposure of a Slovakian cohort to PCB-153, which can antagonize the AhR (20, 69), correlated with a decrease in antigen-specific IgA and IgG titers (62).

It is difficult to impossible to directly evaluate the role of the AhR in Ig secretion in human primary B lymphocytes or in epidemiology studies. Instead, analysis is limited to evaluating AhR levels and activity. Stimulation with toll-like receptor ligands or CD40 ligand and IL-4 increased AhR expression and activity, suggesting an increased sensitivity of stimulated human primary B cells to AhR ligands (66–68). Additionally, primary human CD5^+^ B cells exhibited higher basal and stimulation-induced AhR levels and activity compared to the CD5^-^ B cells, which correlated with increased sensitivity of the CD5^+^ B cells to inhibition of IgM secretion by dioxin (42). Furthermore, a study using a human B lymphoma cell line directly demonstrated dioxin-induced inhibition of IgM secretion that was dependent on AhR expression (43). Although these studies support a role of the AhR in B-lymphocyte differentiation and Ig secretion, the mechanism and impact of the AhR on the production and secretion of other Ig isotypes and subtypes (i.e. IgG1-4, IgA1,2 and IgE) in human is unknown.

In mouse, a key regulator of CSR – activation-induced cytidine deaminase (AID) – was identified as a direct target of the AhR (51). In these studies, B-cell stimulation resulted in greater mRNA and protein levels of AID in AhR knockout mice as compared to AhR wildtype mice. This increase in AID correlated with an increased percentage of IgA^+^ and IgG3^+^ B cells. Additionally, chromatin immunoprecipitation analysis identified AhR binding to a silencer element in the first intron (region 2a) of the gene that encodes AID (i.e. *aicda*). These results suggest that the AhR, independent of exogenous ligand, is a physiological negative regulator of AID (51). Additionally, treatment with dioxin enhanced the inhibitory effect of AhR on *aicda* mRNA and AID protein (51). In contrast, two different groups demonstrated no effect of AhR ligands (dioxin and ITE [2-(1*H*-Indol-3-ylcarbonyl)-4-thiazolecarboxylic acid methyl ester]) on stimulation-induced *aicda* expression (36, 49). How these results will translate to human is uncertain. Our in-silico analysis of the human *AICDA* gene did not identify AhR binding motifs (data not shown). However, this does not preclude the possibility of the AhR binding to DNA through a non-canonical signaling pathway (**Figure 1**, right side of dashed center line).

As summarized above and in **Table 1**, studies demonstrate differential and varied effects of AhR ligands within and among Ig isotypes in rodent and human studies. Perhaps these differential effects are mediated by different molecular interactions between the AhR and other signaling pathways or proteins/transcription factors that are specific to B-lymphocyte subtype and/or external and internal modulators (i.e. antigen and environmental exposures, inflammation, hormones, disease conditions, etc.). Additionally, based on mouse studies, the AhR has been implicated as a determinant of B-cell differentiation with or without exogenous ligand (51, 130). These studies have focused on the role of the AhR in the activity of specific proteins (i.e. AP-1, Bcl6, Prdm1, Bach2, XBP-1, Blimp-1 and Pax5) involved in an all-or-none bistable switch to either activate or inhibit B-cell differentiation and Ig expression in individual B lymphocytes; AhR activation flips the bistable switch to inhibition (18, 31, 130–132). This inhibitory effect has been speculated to result in a more permissive cellular state for memory cell generation (51) or CSR, which may account for reports identifying an increase in certain Ig isotypes by AhR ligands (130) (**Table 1**). However, this bistable switch dictating cell fate may not translate directly to human B lymphocytes. Studies with primary B lymphocytes isolated from different human donors suggest a different mechanism or at least set of protein targets. Like mouse, dioxin increased Bcl-6 and inhibited Prdm1 and XBP-1 expression in human primary B-lymphocytes but unlike mouse, dioxin had no effect on Pax5 or Blimp-1 expression (133–136). Additionally, comparative analysis of dioxin-induced changes in gene expression in mouse, rat and human primary B lymphocytes using RNA-seq supported dioxin-induced inhibition of IgM secretion *via* species-specific pathways (137).

A differential effect of AhR activation on Ig isotypic profiles could lead to disease states such as decreased immune competence (i.e. decreased IgM and IgG) and increased hypersensitivity (i.e. non-specific increase in IgE) as supported by animal models and epidemiology studies (**Table 1**). However, different AhR ligands may also produce different effects. For example, dioxin is not readily metabolized resulting in a prolonged activation of the AhR; whereas dietary and endogenous AhR ligands are more readily metabolized, which has been shown to induce differential effects likely mediated by altered signaling (138). Indeed, in mouse models, the AhR has been shown to directly interact (i.e. protein-protein interactions) with other cytosolic proteins and signaling cascades (e.g. ubiquitin and c-src) and transcription factors (e.g. NF-κB/Rel proteins, AP-1, POU/Oct, NF-1, SP-1, KLF-6, and ER) resulting in alternative signaling from the canonical AhR/ARNT/DRE signaling pathway first discovered in the induction of metabolic enzymes (115, 139–147) (**Figure 1**). The convergence of differential AhR ligands and signaling pathways in B-lymphocyte function and dysregulation is largely unknown. This represents a clinically relevant and significant gap in our knowledge as it impedes our ability to critically assess the risks linked to environmental exposures. Furthermore, understanding the mechanistic links between the AhR and human Ig production would identify sensitive populations and could lead to a novel therapeutic target in controlling Ig-mediated disease states such as PIDs and hypersensitivity/autoimmune diseases. We propose that the human *IgH* gene has evolved to respond to internal and environmental cues and that the AhR is an environmental sensor that adapted, for good or bad, during evolution to relay environmental cues to the *IgH* gene.

## Transcriptional regulation of the *IgH* gene locus, the master gene for all antibody isotypes

Although expression of both a light chain gene and a heavy chain gene is necessary to produce antibodies, the heavy chain gene is responsible for expressing all of the functional Ig isotypes and subtypes. The current understanding regarding transcriptional regulation of the Ig heavy chain gene is largely based on mouse models. In the mouse, regulation of the *IgH* gene is governed through a complex interaction of several regulatory elements, whose activity is B-lymphocyte specific and depends on the cellular maturation state. The most 5’ regulatory element is the variable heavy chain (V_H_) promoter, which lies immediately upstream of the V region and contributes to B-lymphocyte specific activity of the *IgH* locus (148) (**Figure 3**). Regulatory elements with pivotal impact in *IgH* remodeling *via* DNA recombination are the 5′ μ enhancer (Eμ) and the 3′ *IgH* regulatory region (3′RR), an enhancer complex mapped downstream of the cluster of C region genes (**Figure 3**). Activation of the 5′ Eμ promotes VDJ recombination during the earliest steps of B-cell ontogeny and is temporally followed by the activity of the 3′RR, which controls differentiation processes such as CSR and SHM (149–159). AID mediates both SHM and CSR by converting cytosines into uracils and inducing DNA repair mechanisms. Unfaithful repair leads either to incorporation of point mutations in the VDJ region (altering the affinity of the antigen-binding pocket, i.e. SHM) or to DNA recombination at switch sequences (linking a different C-region to the VDJ region, i.e. CSR). Interleukin signaling and a network of transcriptional regulation promote CSR within the *IgH* gene to produce specific Ig isotypes (160, 161). As discussed above, both internal and external factors likely influence these processes, perhaps because of selective pressure to allow more complex organisms to adapt to their environment.

### Evolution of the *IgH* region and emergence of a regulatory node

Multicellular organisms need to defend themselves against infection by pathogens, but only vertebrates mount sophisticated defenses including specialized cells for an acquired humoral immune response (162). Specific to humoral immunity, the *IgH* gene is expressed in all Gnathostomata (jawed vertebrates) (162). Despite common mechanisms, somatic diversification, the number of antibody isotypes, and tertiary antibody structure vary in individual species, demonstrating substantial divergence and propensity of segmental rearrangements during evolution (163–169). However, the location and order of the heavy chain C-region genes does not vary as could be expected. In Tetrapods (terrestrial vertebrates) and bony fish, a single *IgH* gene locus evolved to contain multiple C-regions, which are mainly expressed by CSR (166, 170). Additionally, most species have homologs of the most 5′C-region genes, i.e. μ and δ, which are the only two C-regions that are expressed *via* alternative mRNA splicing rather than CSR. Cartilaginous fish on the other hand have multiple *IgH* genes in different chromosomal locations (166, 170).

The genome and gene duplications that occurred in early vertebrates (171) may have given rise to an interesting coincidence of multiple *IgH* and AhR genes in cartilaginous fish. Unlike the multiple *IgH* genes that are not conserved beyond cartilaginous fish, multiple *AhR* genes appear to have been maintained in many species; however, xenopus, mouse, gorilla, and human appear to be limited to having only one *AhR* gene (171). Additionally, in line with the development of adaptive immunity in Gnathostomes, the *AhR*, which is a far more ancient gene, also adapted to bind environmental chemicals and induce metabolic enzymes as an additional protective mechanism (171, 172). It has also been previously proposed that a regulatory role of the AhR in the immune system may have co-evolved with the development of adaptive immunity (171). In support of this, dioxin treatment of a zebrafish autoimmune model (i.e. Teleost bony fish) increased FoxP3 expression and decreased IL-17, indirectly supporting AhR involvement in developing peripheral tolerance to the generation of autoantibodies (173). Other studies with different bony fish species demonstrated a dependence on the AhR for an effective immune response against bacterial pathogens (174, 175). Ig and B cells were not directly evaluated in these studies.

Evaluation of the *IgH* region across Tetrapods (terrestrial vertebrates) demonstrates an increase in C-region genes as well as duplications within specific heavy chain classes (170, 176, 177). This expansion in C-regions gave rise to some pseudo, nonfunctional C-regions but also to an expanded repertoire of functional C-regions depending on the species and presumably the advantage of the adaptation (165). Interestingly, the order of the C-region homologs is fairly conserved, i.e. 5′ V-D-J variable segments followed by C-regions of μ, δ, γ, ε, α classes. Additionally, substantial species-specific variations in the number of subclasses are evident. For example, mice and humans carry four γ genes whereas horse has 7 γ genes (178–180). Rabbits carry 13 α genes compared to one in mice and two in humans (179–181). It is worth noting that the C-region remains prone to segmental variations of both loss and gain that results in divergence within the same species (182). Genetic drift or selection appears to have resulted in segmental variations that are not sporadic but are a characteristic that may be reflected in a specific allelic frequency in different populations (183). It appears that, at least in human, the *IgH* region can still evolve toward loss or gain of C-regions and regulatory regions (182–184).

**Figure 4:**
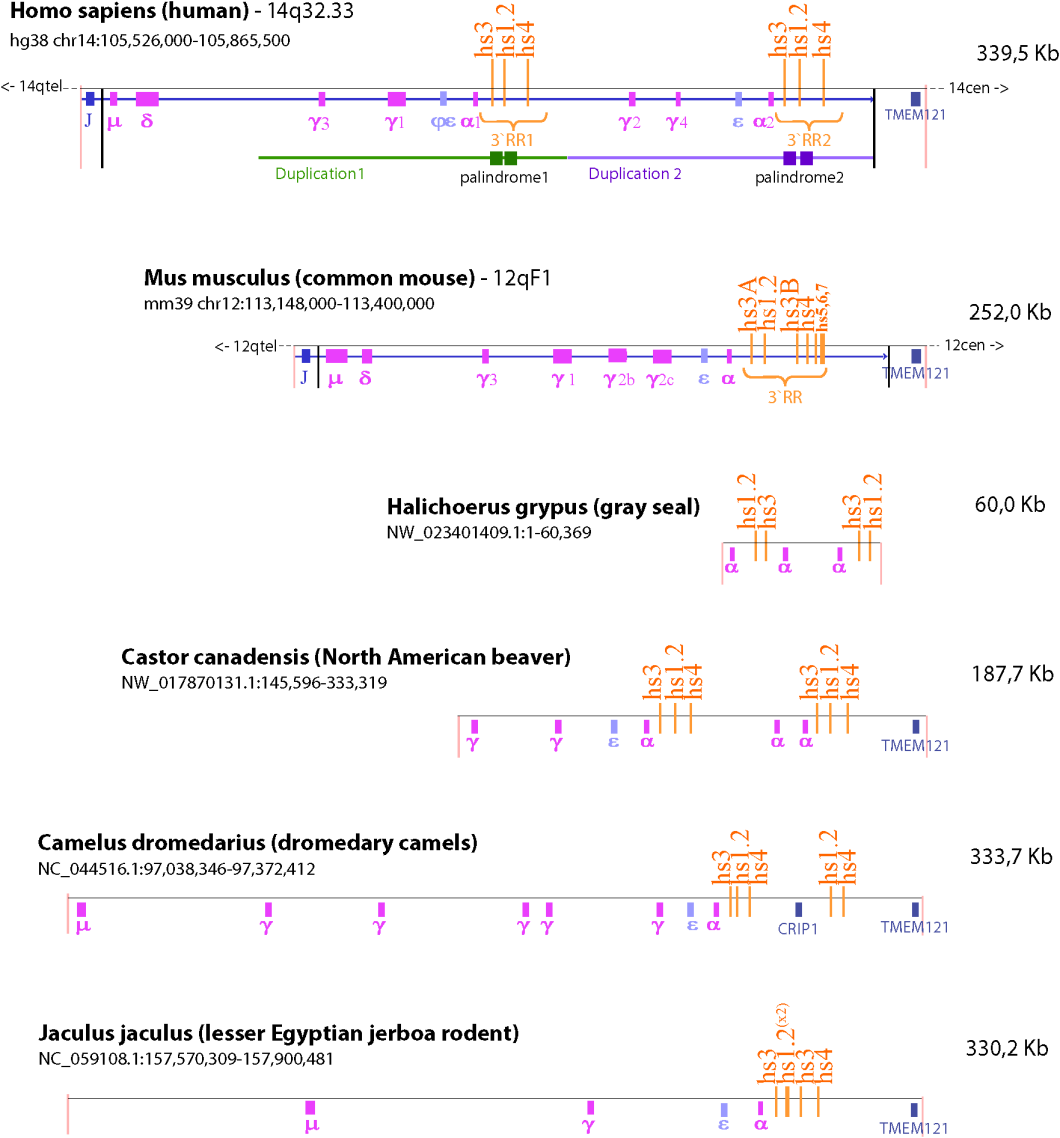
Species comparison of the *IgH* gene locus. The schematic illustrates the similarities and differences in the number of *IgH* constant regions and composition of the 3’RR among six different species (i.e. human, mouse, gray seal, North American beaver, dromedary camel, and lesser Egyptian jerboa rodent). The NCBI refseq number and location of the sequence depicted are identified under the species name. TMEM121 was used as a reference of synteny because it is the gene closest to the *IgH* region. Duplication of or within the 3’RR occurred in both human and mouse. However, the murine 3’RR has an internal duplication that produces two copies of the hs3 enhancer (hs3A and hs3B); whereas the human genome contains two 3’RRs (i.e. 3’RR1 and 3’RR2) due to a large duplication of constant region genes and the hs3, hs1.2 and hs4 enhancers. Analysis of species quite far in phylogenesis identified the presence of distinct and varied duplications in the *IgH* gene that include duplications of constant regions and the 3’RR enhancers. This wide range of arrangements within the *IgH* gene among different species suggests that many of these duplications happened independently in various branches of the evolutionary tree.

Our understanding of the structure and function of the *IgH* gene relies heavily on mouse models. However, there are significant structural differences between rodent and human *IgH* loci that is worth considering when translating results from the mouse model to humans. Evolutionarily this difference arose from a marked divergence in the *IgH* region between the Platyrrhini Parvorder (New World monkeys, e.g. squirrel monkeys, wooly monkeys, marmoset) and the Catarrhini Parvorder (apes, humans and Old World monkeys, e.g. rhesus monkeys) because of a large duplication of a portion of the *IgH* region. This divergence allowed for the emergence of additional antibody subclasses (i.e. IgG3, IgG4 and IgA2) in the Catarrhini Parvorder (185). Moreover, the same segmental structural variation involved the 3′RR region, giving rise to the 3′RR1 and 3′RR2 that are present in all Catarrhini (186). We found that species quite far in phylogenesis, like grey seal, dromedary camel, North American beaver, and lesser Egyptian jerboa also show the presence of a duplicated 3′RR region (**Figure 4**). Since these duplications are not uniform in size or breakpoints, they are likely a result of independent events during genome rearrangement that happened after divergence of the specific lineages for each species. These duplications resulted in redundance and retention of functional sequences in the 3′RR and therefore may provide additional mechanisms for controlling antibody production (187–190).

The current reliance on non-primate models and non-Hominoidea primates may not entirely translate to human B lymphocyte biology and antibody production because of the genetic divergence originating from the segmental duplication of the *IgH* locus. This is of concern considering that toxicological studies and preclinical therapeutic and safety evaluations utilize mainly rodent models, specifically mouse and rat, as well as marmoset or other monkey species that do not have this large duplication and therefore may not accurately reflect human antibody regulation and production (168, 185). Moreover, the human relevance of non-Hominoidea models may also be limited because of divergence among members of the Catarrhini Parvorder, e.g. the pseudogenization of some *IgH* gene segments or the large number of detected variants within the 3′RR that seem species-specific (186, 191). Similarly, in examining the role of the AhR in influencing immune function, not only species differences in the *IgH* gene but as mentioned above species differences in AhR ligand binding and specificity as well as the number of AhR genes are also factors to consider when translating non-human studies to human. For example, although there is only one AhR gene in the mouse as in human, there are differences in AhR ligand binding affinity and specificity between the two species (**Table 2**). In terms of primates, marmoset (New World Monkey) and green monkey (Old World Monkey, Chlorocebus Family) differ from humans in that they express two AhR genes (171). The number of AhR genes in other primates as well as the binding affinity/specificity for ligand in primates in general has yet to be determined.

**Figure 5:**
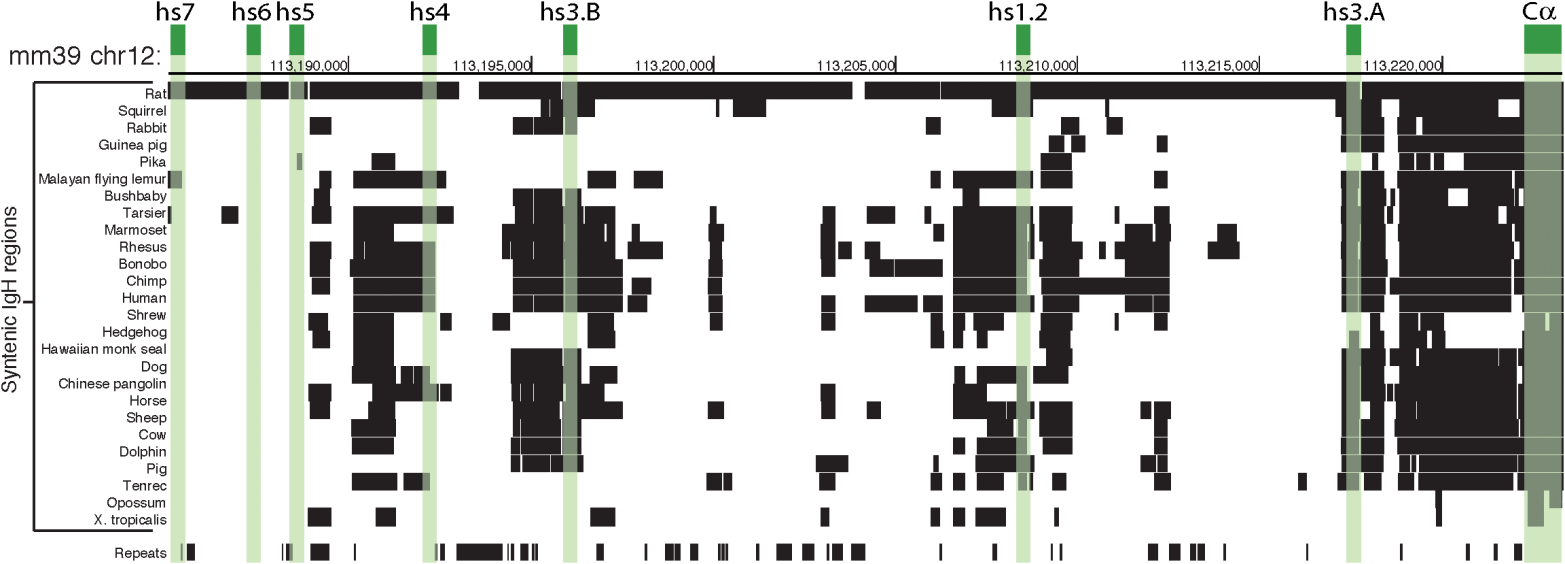
Mouse 3’RR and syntenic region in Vertebrata. The schematic is focused on a comparative analysis of the 3’RR and α constant region (Cα) within the *IgH* gene locus. Green boxes highlight the position of the 7 enhancers and Cα in the murine chromosome 12 (mm39 genome assembly). The rows below show alignments of mm39 to other genomes using a gap scoring system (see UCSC Net track for details); detected orthologous regions are indicated by black boxes. Three of the enhancers (i.e. hs5, hs6, and hs7) are specific to mouse and rat, while the other enhancers (i.e. hs3A, hs1.2, and hs3B) appear shared among more species, almost to the same level as Cα. The ‘Repeats’ row indicates segments of the murine *IgH* region that are detected as ‘repeats’ (e.g. SINEs, LINEs, satellites) by RepeatMasker software (https://www.repeatmasker.org) and could be ignored in a synteny comparisons because of their characteristics.

The presence in the human-lineage of the two 3′RR copies, perhaps *via* transposition of the primordial μ enhancer along with duplications, could be the preparatory steps that provided the necessary regulatory elements to mediate CSR and enable the diversification of functional roles and timing of different Ig isotypes [reviewed by (192)]. The 3′RR demonstrates both remarkable conservation and divergence among species (**Figure 5**). The number of enhancers within the 3′RR varies, e.g. the mouse and rat have 7 enhancers (hs3A, hs3B, hs1.2, hs4, hs5, hs6, hs7), while humans have 3 enhancers (hs3, hs1.2, hs4) (**Figures 4**, **5**). Orthologues of the human hs3, hs1.2, and hs4 enhancers were found in mammals belonging to non-primate species, including rat, mouse, cat, dog, panda, rabbit, and bat (**Figure 5**) (186). Additionally, a large area internal to the 3′RRs showed a conserved presence of an inverted duplication (i.e. palindromic region) surrounding the hs1.2 enhancer, which was found in all of the analyzed species, suggesting a conserved characteristic, though the actual sequence of the palindrome varies (186). The palindromic region could form a hairpin loop, suggesting that the orientation of the hs1.2 enhancer would be irrelevant for enhancer function (186). Indeed, the hs1.2 enhancer is in different orientations in different species and even between the two human 3′RRs (193). The latter finding may also suppress the enhancer conversion between the two copies and favor the divergence between the 3′RRs (194). Strikingly, a specific evaluation of the sequence conservation among distant species in the hs1.2 consensus site for transcription factor binding showed conservation in the core of the enhancer, but high variability in the GC-rich regions that harbor most of the transcription factor binding sites (data not shown). Hypothesizing the palindromic sequences to be the stem of a hairpin, the hs1.2 enhancer region would be in a loop protruding from the chromatin, thus easily accessible to transcription factors that can bind to single-stranded DNA. This protruding three-dimensional structure could also facilitate long-distance cis-interactions with other *IgH* gene regulatory elements (e.g. V_H_ promoter, Eμ, and intronic promoters associated with each constant region) to promote *IgH* expression and CSR (152, 195–199) as well as SHM within the *IgH* gene (197, 200, 201). The finding in mammalian genomes of syntenic regions of palindromes having sequences unmatching when compared among different species suggests that mutations on one branch of the palindrome were often compensated by mutations on the other branch, preserving the complementarity and consequently the stem of the hairpin (186).

**Figure 6:**
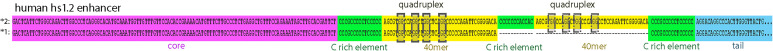
Sequence and notable regions of the hs1.2 enhancer. Allele *1 and *2 of the human hs1.2 enhancer are illustrated to highlight the presence of the internal 40 bp monomer (40mer), which has the potential to form quadruplex DNA (i.e. a stable secondary DNA structure). A “core” (magenta) and a “tail” (light blue) sequence are common to all the variants, while the “40mer” repeat (yellow box) may be present from one to four times in human.

**Figure 7:**
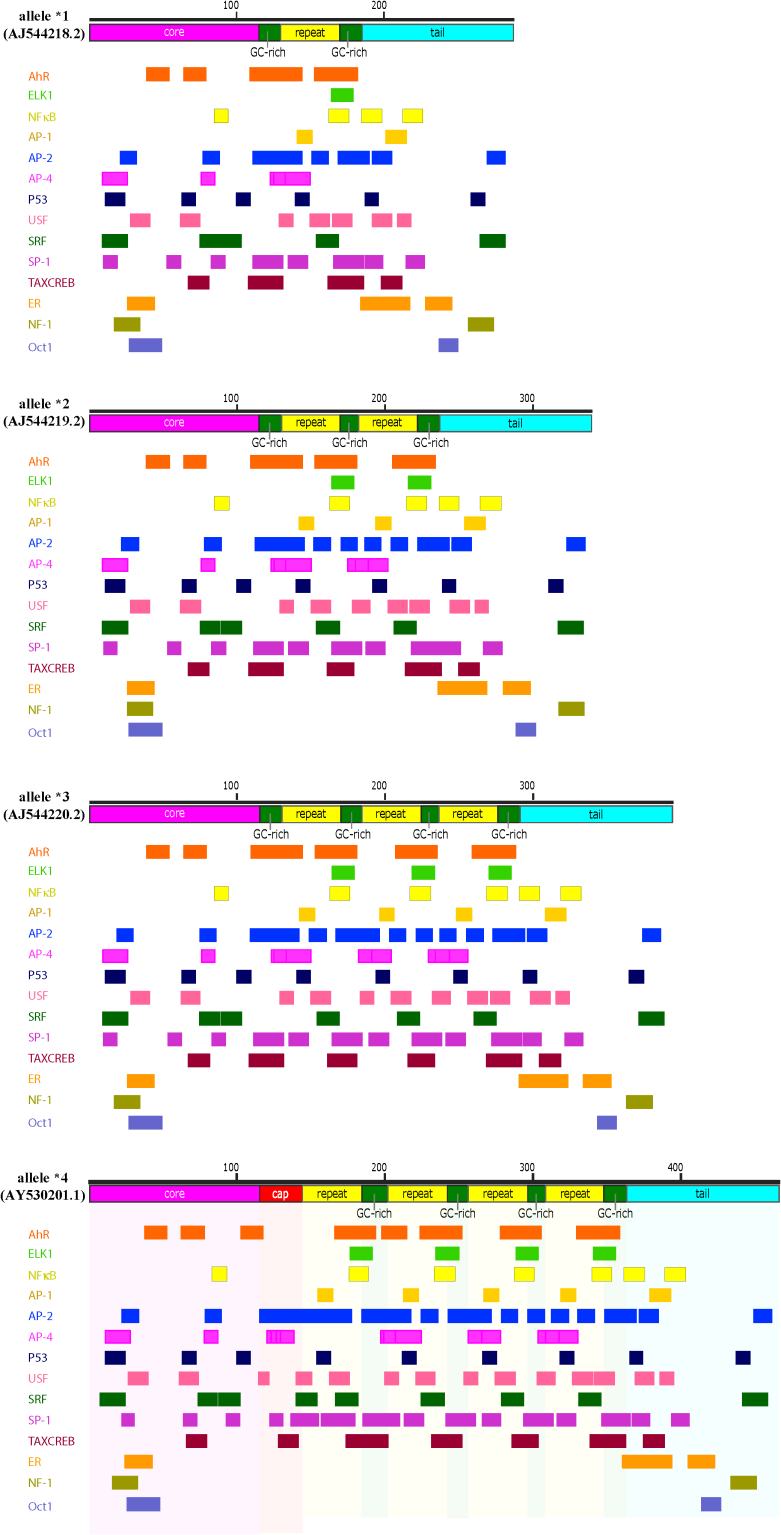
Transcription factor consensus binding sites within the human hs1.2 enhancer alleles. Depicted are the four known alleles of the human hs1.2 enhancer, which differ mainly in the copy number (“repeat”) of the internal 40 bp monomer. A “core” (magenta) and a “tail” (light blue) sequence are common to all the variants, while the yellow box (“repeat”) may be present from one to four times in human. However, at present, the highest copy number has been detected in the chimpanzee hs1.2 enhancer, which has twelve repeats. The green boxes are GC-rich regions. The red “cap” box is a GC-rich region longer than the ones indicated by the green boxes. The alleles were analyzed in silico for potential transcription factor binding sites using TFBIND (https://tfbind.hgc.jp/). The consensus binding sites for several transcription factors were identified and reported below each allele. Multiple partially overlapping sites for the same transcription factor were merged and depicted as a single larger box (See **Supplementary File S2** for unabridged depiction of consensus binding sites of the allele *4). Consensus sites mapping in the hs1.2 “repeat” generally change in copy number according to the number of monomers, thus potentially making the enhancer alleles differentially receptive to transcription factors. Moreover, each copy of the monomer harbors a region that could form a quadruplex DNA 3-D structure (see **Figure 6**).

The 3′RRs and their unique structural features are likely controlled by binding of several cis-acting transcription factors (186, 201, 202). Furthermore, there is evidence of the formation of a G-quadraplex structure within the hs1.2 enhancer that seems a maintained feature due to the persistent presence of GC-rich short sequences inside the hs1.2 enhancer (i.e. GC-rich region in **Figure 6**) (186, 197). The human hs1.2 enhancer has putative binding sites for several transcription factors (SP-1, AP-1, NF-1, NF-κB, AP-1.ETS, POU/Oct, AhR, ER), some of which have been confirmed to actually bind transcription factors. Additionally, mouse studies have identified binding of the ER and the AhR, both of which may directly transduce internal and environmental signals through the 3’RR regulatory node (146, 187, 193, 203–214) (**Figures 7**, **S2**). The binding dynamics and interplay of the various transcription factors that could bind within the native hs1.2 enhancer remains to be elucidated, particularly in human. Some transcription factors could bind the hs1.2 region when in a double-stranded DNA conformation, thus opening the chromatin and allowing for the formation of the hairpin loop or the G-quadruplex. At least for Sp-1 and AP-1, their high affinity binding to G-quadruplexes has been demonstrated (201, 215, 216). Alternatively, the three-dimensional structure could prevent binding of transcription factors, carrying out a structural regulation of the protein-DNA binding. These DNA structural changes may not be replicated in reporter studies due to the lack of chromatin and the inability to include the whole native form of the 3′RR due to its large size (~17 kb). Additionally, polymorphisms within a putative quadruplex locus have been associated with altered gene expression further supporting the role of these three-dimensional structures in transcriptionally regulating genes (217–219). Notably, the human hs1.2 enhancer exhibits genetic polymorphisms, not present in the mouse, that involve the quadruplex site and appear to influence the transcriptional activity of the 3′RR (186, 187, 197, 220, 221).

The hs1.2 enhancer polymorphism relies on a 40 bp monomer that in human can be repeated in tandem up to four times (**Figure 6**) (186, 187, 193, 220, 222–224). This finding corresponds to four possible human hs1.2 enhancer alleles presenting either one, two, three or four monomers linked together (allele *1, *2, *3 and *4, respectively, **Figure 7**) (186, 193). The 40 bp monomer repeat contains putative binding sites for several transcription factors including the AhR (**Figure 7**). In the human 3′RR1, all four alleles have been identified, whereas only alleles *3 and *4 seem to be present in the 3′RR2 (221). Moreover, the frequency of each 3′RR1 hs1.2 allele appears to be different among ethnic groups (221). European populations exhibited a higher frequency of alleles *1 and *2; African populations instead exhibited a higher frequency of alleles *1, *3, and *4 (221). Furthermore, the presence of allele *2 in 3′RR1 in primarily European populations has been associated with increased serum Ig, hypersensitivity, and autoimmune disorders, making this region an attractive predictive/therapeutic target (209, 214, 223, 225–235).

### Genetic variation in the 3’RR correlates with antibody levels and disease

The presence of specific 3′RR1 hs1.2 enhancer alleles have been associated with various disease states and antibody levels. For instance, celiac disease, dermatitis herpetiformis and psoriasis correlated with a higher frequency of hs1.2 allele *2 (227, 229). Compared to controls, a significant increase in IgM levels in patients with lupus erythematosus and systemic sclerosis was associated with the presence of the hs1.2 allele *2 (214, 228). In the above pathologies, more severe symptoms associated with homozygosity for allele *2, which was also the case in the severity of IgA nephropathy towards renal failure (225). However, it should be noted that the frequency of alleles *3 and *4 was very low in the patient population evaluated. Therefore, the analysis was primarily limited to evaluating alleles *1 and *2. In other studies, analysis of single nucleotide polymorphisms (SNPs) in the 5kb region that connects the human hs1.2 and hs3 enhancers identified a correlation between psoriasis and the presence of specific 3′RR haplotypes. The SNPs correspond to potential methylation sites that could influence conformational changes and may be a target of environmental factors (235). Interestingly, the SNPs involved specific 3′RR haplotypes with a very low presence in the African population as evidenced by GnomAD loci mapping (235), corresponding to a low rate of both allele *2 and psoriasis in West African populations (221, 236). A recent study also found that specific 3′RR haplotypes within the 5kb region connecting the hs1.2 and hs3 enhancers preferentially associated with allele *1 or with allele *2 (237).

A human population study examined the linkage of the 3′RR haplotypes with allotypes of the γ1 and γ3 constant regions in different continents (238). Allotypes are polymorphisms within the Ig heavy and light chains in the same species (239). As seen in previous studies (221), the highest frequency of hs1.2 allele *2 is in Europe with the lowest in Africa (238). Interestingly, a linkage between 3′RR1 haplotypes and γ1 and γ3 constant region allotypes was demonstrated in populations outside of Africa but not within Africa, suggesting that this large region of DNA underwent positive natural selection as humans dispersed out of Africa (238, 240). Analysis of cell lines from the 1000 Genomes Project identified higher expression of γ1 and γ3 constant regions in cells homozygous for the hs1.2 allele *1 vs. the other hs1.2 alleles (238). However, the expression of functional transcripts and antibody levels were not determined.

Evidence of a correlation between antibody levels and hs1.2 alleles was identified in a longitudinal study of healthy children before and after 5 years of age, which appears to be divergent from the increase in γ1 and γ3 expression with hs1.2 allele *1 mentioned above. Before 5 years of age, children with high blood concentration of IgM, IgG and IgA had a frequency of hs1.2 allele *2 around 80% with an increase in frequency of approximately 30% above the control population of that geographical area. Surprisingly, the same children two years later did not have this correlation between allele *2 and high blood concentrations of IgM, IgG, and IgA and no such correlation was present after 7 years of age; a large cohort of healthy students (aged 19 to 33 years) also exhibited no correlation between allele *2 and high Ig titers (234). Serone and co-workers (234) theorize a selective and limited age-dependent advantage of allele *2 in populations that migrated out of Africa and were exposed to a change in the environment and infectious pathogens. This may have resulted in more protection during childhood but with increased life expectancy a potential dysregulation of this age-dependent regulation could lead to increased risk of autoimmune disease (234). Due to the different hs1.2 alleles primarily varying in the number of transcription factor binding sites (**Figure 7**), it is tempting to speculate that environmental factors such as AhR ligands can lead to this dysregulation and increased risk of altered antibody levels and disease.

### Role of the 3’RR in transcriptional regulation of the *IgH* gene

Transcriptional regulation of the *IgH* gene has been largely defined in rodents. The mouse 3′RR consists of a set of enhancers, either rodent-specific (hs5, hs6, hs7) or conserved in vertebrates (hs1.2, hs3, hs4) (**Figure 5**) and has been functionally evaluated by many *in vitro* and *in vivo* approaches (198, 210, 241–244). For the mouse 3′RR, the hs1.2, the two copies of hs3, and hs4 are individually weak enhancers, but when all four are linked together they become strong co-activators (154, 241, 245, 246). Deletion of these four enhancers in mice results in a dramatic decrease in CSR and in secretion of all antibody isotypes (149–154, 156–159). Studies have also demonstrated a long-range intrachromosomic interaction between the 3′RR and the V_H_ and intronic promoters (152, 198, 211, 247). The V_H_ promoter is essential to producing functional *IgH* transcripts, i.e. transcripts that can be translated into Ig heavy chain proteins. The intronic promoters located just upstream of each *IgH* constant region produce nonfunctional or germline transcripts that include the switch sequences and do not lead to the production of functional proteins (**Figure 3**). Germline transcription at the intronic promoters is thought to open the chromatin to allow for and direct CSR to a specific *IgH* constant region and expression of a specific antibody isotype (248, 249). Regulation of intronic promoter activity by the 3′RR appears to involve either co-activation or competition depending on the activating stimuli, which may have evolved to respond to T-independent vs. T-dependent immune responses (250). Germline transcripts have been shown to form R-loops at their corresponding switch sequences resulting in a G-rich DNA single strand that is accessible to AID, which the 3′RR plays a role in recruiting (151, 251–253). Additionally, specific enhancers within the 3′RR may have greater influence on the expression of specific constant regions (154, 254). Taken together these studies indicate an essential role of the mouse 3′RR in *IgH* expression, CSR and therefore final antibody production by plasma cells (255).

The mouse 3′RR is also sensitive to modulation by environmental factors (**Table 3**). AhR ligands have been shown to markedly inhibit 3′RR activity, which mirrored their inhibitory effect on *IgH* expression and antibody secretion (19, 33, 57, 146). Besides AhR ligands, other chemicals altered 3′RR activity that directly correlated with their effects on *IgH* expression and antibody secretion. The reactive oxygen species hydrogen peroxide exhibited a biphasic effect, with low concentrations inducing and higher concentrations inhibiting 3′RR activity and antibody secretion (256). Gold nanoparticles and the β-adrenergic receptor agonist terbutaline induced 3′RR activity and antibody secretion (57, 257). These environmental factors likely influence distinct signaling cascades that alter binding of transcription factors within the 3′RR resulting in changes in *IgH* expression. Additionally, the nature of the cellular stimulation initiated by a response to antigen (i.e. TLR, co-stimulation through CD40 and/or CD86 or cytokine receptors) is expected to be a factor on the transcription factor milieu and the effects of environmental factors. Multiple transcription factors such as the AhR, NFκB/Rel, POU/Oct, Pax5 have been identified as positive and/or negative regulators of the mouse 3′RR, which may be dependent on the maturation stage of the B cell and the specific enhancer. Co-activation of β-adrenergic receptors (environmental factor) and of CD86 (antigen stimulation) induced binding of OCA-B (Oct-1-associated coactivator)/Oct-2 dimers to octomer motifs within the mouse hs1.2 and hs4 enhancers (206). Additionally, AhR activation in the presence of LPS stimulation altered NFκB/Rel protein binding within the hs1.2 and hs4 enhancers by reducing RelB binding and increasing RelA binding, which corresponded to altered enhancer activity (146). Other studies evaluated the effect of AhR activation on Pax5 protein levels. Pax5 is a negative regulator of mouse 3′RR activity and antibody production but B-cell activation and differentiation into plasma cells results in decreased Pax5 expression, thus lifting the repressive effects of Pax5 on 3′RR activity and antibody production (204, 207, 258–262). AhR activation in combination with cellular stimulation reversed the stimulation-mediated decrease in Pax5 expression, which correlated with the inhibitory effect on *IgH* expression by AhR ligands (18, 31).

**Table 3:** Internal and external modulators of mouse and human 3’RR and its enhancers.

Internal and External modulators	Mouse			Human		
	3’RR	hs1.2 enhancer	hs4 enhancer	3’RR	hs1.2 enhancer	hs4 enhancer
AhR ligands**^1^**	↓^*^	↓^*^	↑^*^	ND	↑^*^	ND
Gold nanoparticles	↑	ND	ND	ND	ND	ND
Hydrogen peroxide^2^	[low] ↑ [high] ↓	ND	ND	ND	ND	ND
Terbutaline (β-adrenergic receptor)	↑	ND	ND	ND	ND	ND
Estrogen	↑ ↓^^^	ND	ND	ND	ND	ND
B-cell stimulation	↑	↑	↑	↑	↑	ND

^1^For the mouse 3’RR, the AhR ligands analyzed included dioxin, indolo[3,2-b]carbazole, omeprazole, and primaquine. Only dioxin was evaluated for the mouse and human hs1.2 enhancer and the mouse hs4 enhancer. ^2^Low concentration increased 3’RR activity and high concentration decreased 3’RR. *, AhR-dependent based on AhR KO or AhR antagonist. ^mutation of ER binding site decreased CSR but estrogen treatment also decreased CSR. ND, not determined.

Furthermore, studies have identified binding of ER to various areas of the mouse *IgH* gene including Eμ, switch sequences and the hs1.2 enhancer (212, 263). Mutation of the ER binding sites within the Eμ or hs1.2 enhancer decreased CSR to IgA in a mouse B-cell line model (264). However, LPS stimulation with exogenous estrogen reduced IgG3, IgG1 and IgA secretion in purified B cells from female mouse spleens, but over an age span of 6 weeks to over 5 months, female mice had higher serum titers of most antibody isotypes compared to male mice (265). A regulatory role of ER in the *IgH* gene may be particularly relevant to the gender differences seen in incidence of autoimmune disease and effectiveness in fighting infections.

These studies evaluating the transcriptional regulation of the *IgH* gene have primarily utilized mouse models. The significant genetic differences between the mouse and human *IgH* genes will likely result in differences in regulation and responses to external stimuli (**Table 4**). As described earlier, the two most striking differences are that 1) the human 3′RR is duplicated along with several constant regions and 2) the 3′RRs contain three instead of seven enhancer elements (i.e. hs3, hs1.2, hs4) (**Figure 4** and **S1**) (80, 186, 187, 220, 222). Like the mouse 3′RR, the human 3′RRs are thought to physically interact with intronic promoters (266). Presumably each 3′RR regulates CSR and gene expression of a specific set of C regions. However, analysis of CSR in humans with a homozygous deletion of the 3′RR1 suggested that the 3′RR2 could compensate for this loss (248, 267). Furthermore, the mouse 3′RR was shown to be capable of bidirectional activation of intronic promotors using a mouse model with an insertion downstream of the 3′RR of a β-globin gene regulated by an intronic promoter (250). Taken together, these observations suggest that the human 3′RR1 is capable of regulating transcription of upstream and downstream constant regions and perhaps the 3′RR2 only comes into play when the 3′RR1 is deleted by CSR to constant regions downstream of 3′RR1.

**Table 4:** Comparison of mouse versus human 3’RR characteristics and effect of AhR activation.

3’RR Characteristics and Function	Mouse	Human
3′RR mediates CSR and SHM	Yes	ND
Duplicated 3′RR	No	Yes
Polymorphic hs1.2 enhancer	No	Yes
Disease associated with hs1.2 enhancer polymorphism	No	Yes
Effect of AhR activation on hs1.2 enhancer	Inhibit	Activate Allele *1>*2>*3>*4
Effect of AhR activation on 3′RR	Inhibit	ND

Reporter plasmids have been used to evaluate the transcriptional activity of the human 3′RR enhancers in isolation or linked together. Like the mouse 3′RR enhancers (hs3A, hs1.2, hs3B, hs4), these studies suggest a strong cooperative activation when all of the human 3′RR enhancers (hs3, hs1.2, hs4) are linked together (266). However, the majority of these studies have only evaluated the basal activity of the individual or linked 3′RR enhancers and none of these studies have evaluated the native 3′RR (i.e. enhancers with intervening sequences) mostly due to the large size of the native human 3′RR (~17 kb) (**Figure S1**) (187, 188, 208, 220, 222, 223, 266) (**Table 3**). This limitation of reporter plasmids is particularly important when considering the evolutionarily conserved palindromic sequences flanking the hs1.2 enhancer, which, as discussed earlier, likely play a role in the three-dimensional structure of the 3′RR and promoting long-range interactions with other *IgH* gene regulatory elements (152, 186, 195–199, 201). However, in contrast to the *in vivo* mouse models, there are limited human cellular models to study the role of the 3′RRs on molecular processes (i.e. *IgH* expression, antibody secretion, CSR and SHM) involved in antibody production. An additional consideration is the genetic polymorphisms within the human hs1.2 enhancer, which, as discussed in the previous section, has been associated with Ig expression levels and several diseases (186, 193, 220, 222–224). Therefore, the genetic profile of the human hs1.2 enhancer may be a key influencer on the sensitivity of the 3′RR and Ig expression, as well as risk for developing antibody-mediated disease from exposure to environmental stressors.

## Environmental factors and transcriptional regulation by the human hs1.2 enhancer

Limited studies have evaluated the impact of genetic differences within the human hs1.2 enhancer on transcriptional activity or the role of specific transcription factors in mediating hs1.2 enhancer activity. *In vitro* analysis of human hs1.2 enhancer activity using reporter plasmids demonstrated that the hs1.2 alleles harboring a higher copy number of the 40 bp monomer have increased transcriptional activity (i.e. allele *1 < allele *2 < allele *3 < allele *4, see **Figure 7**) (223, 225). The monomer(s) is flanked by SP-1 sites and contains possible binding sites for the transcription factors NF-κB/Rel, AP-1, and NF-1 (187, 193, 220, 222, 223). As mentioned previously, we identified a potential AhR binding site, i.e. DRE, within the repeated monomer sequence of the human hs1.2 enhancer. This DRE motif is similar to the functional DRE we previously evaluated in the mouse hs1.2 enhancer (19, 34, 146). Furthermore, Jones and coworkers (265) identified a potential ER binding site within the monomer of the human hs1.2 enhancer, which overlaps with the suggested AP-1 binding site (223). As discussed earlier, the AhR binds a plethora of ligands from environmental, dietary, and pharmaceutical sources. The ER is also known to be promiscuous in ligand binding, which has generated considerable public concern regarding the potential of chemicals that bind and activate the ER (e.g. bisphenol A) to disrupt endocrine function (268, 269). In the context of antibody production, the AhR and ER could transduce a variety of signals through the hs1.2 enhancer to affect 3′RR activity and *IgH* regulation. What may have originally evolved as an internal and environmental sensor for adaptation and host protection, may now be a target for dysregulation by environmental and industrial chemicals and pollutants.

Consistent with a functional impact of transcription factor binding within the 40 bp monomer, deletion of the monomer in allele *1 or deletion of one of the repeated monomers in allele *2 of the hs1.2 enhancer resulted in decreased transcriptional activity (19). Limited studies have evaluated the effect of cellular stimulation or chemical exposures on the activity of the human hs1.2 enhancer alleles. Unlike the inhibitory effect of dioxin on the mouse hs1.2 enhancer, dioxin induced a significant increase in overall human hs1.2 transcriptional activity that was dependent on the AhR and the allelic state of the hs1.2 enhancer (**Tables 3**, **4**); however, the AhR does not appear to bind the DRE-like site within the monomer (19, 213). Mutational analysis suggested a partial role of the NF-1 binding site and not the DRE-like site in the AhR-mediated induction of hs1.2 activity (213). It is likely that the AhR mediates its effects through protein-protein interactions (e.g. ER, NF-κB, AP-1, POU/Oct, NF-1, SP-1, and KLF-6) rather than directly binding to the DNA (see **Figure 1**, right side of the dashed center line) (115, 139–147). These results are contrary to the demonstrated binding of the AhR to the DRE within the mouse hs1.2 enhancer and the inhibitory effect of dioxin on enhancer activation following cellular stimulation, which further supports species differences in the regulation and activity of the 3′RR (19) (**Tables 3**, **4**). Additionally, analysis of the hs1.2 sequence suggests that there is no site-specific pressure towards the conservation of the DRE consensus (i.e. GCGTG), but the characteristic sequence of the hs1.2 enhancer (modularity and repeated GC-rich regions) easily give rise to a DRE-like consensus. Thus, DRE consensus sites appear to vary in the hs1.2 from different species in both position and number and whether it is even present (data not shown). Our analysis suggests a similar effect on ER binding sites. Additionally, in contrast to Jones and co-workers (265) who reported a relatively conserved ER binding element in the 40 bp monomer of the hs1.2 enhancer when considering mouse, Callicebus moloch (New World Monkey), gorilla, chimpanzee, and human alleles *1 and *2, our in-silico analysis using TFBIND (https://tfbind.hgc.jp/) only identified ER consensus sites outside of the monomer repeat (**Figure 7**). It is worth noting that actual binding of the ER has only been evaluated in mice (212, 263, 265). These results support species differences in transcription factor binding and activity of the hs1.2 enhancer, whose regulation may have evolved through slightly divergent pathways to adapt to environmental signals. How these species differences relate to an overall effect on antibody production is yet to be determined.

Mutational analysis studies of specific binding sites within or outside of the sequence repeats identified positive and negative regulators of human hs1.2 reporter activity; however, the different reporter studies demonstrated contradictory effects on hs1.2 activity likely due to variations in the flanking regions and number of sequence repeats incorporated into the reporter, differences in cellular models, and the inability of a gene reporter to accurately reflect the interactions of different transcription factors within and among the different enhancer regions of the 3′RR (187, 208, 213). Additionally, our in-silico evaluation of the human hs1.2 alleles for consensus transcription factor binding sites identified multiple potential binding sites for the AhR, NF-κB, AP-1, NF-1, and ER, as well as other transcription factors (**Figures 7**, **S2**). The transcriptional effect of these sites has not been fully evaluated and our analysis did not identify the previously suggested sites for NF-1 and ER within the monomer repeat (213, 223, 265). Further studies are needed to fully characterize the transcription factors regulating human hs1.2 activity, including the impact of allelic variants and the response to internal and environmental factors, with the goal of elucidating how these factors impact *IgH* expression, SHM, and CSR.

Snyder and coworkers (213) suggested that the polymorphic region of the hs1.2 enhancer plays a fine-tuning role in the regulation of the hs1.2 enhancer because of the strong activating role of the AP-1.ETS site vs. the inhibitory role of the POU/Oct site, which are both outside of the polymorphic region (i.e., 40 bp monomer repeat). The fact that the polymorphic region can expand the number of transcription factor binding sites (e.g. AhR, NF-κB, AP-1) may produce more of a regulatory role on overall hs1.2 activity and lead it to be more sensitive to environmental cues. This would seem to be a favorable feature considering that the humoral immune response should be adaptive and responsive to external insults that are able to evade the body’s first line of defense. Additionally, antibody production should also be responsive to the signals produced by the microbiota because of the role antibodies play in maintaining a healthy balance of commensal microorganisms in the mucosal tissues. Furthermore, it is not surprising that B lymphocytes would be responsive to internal signals from the endocrine system as seen with adrenergic receptor agonists and estrogen (57, 206, 212, 263, 265). In line with this premise, a recent study of COVID-19 patients associated the presence of hs1.2 allele *2 to decreased disease severity (based on the need of oxygen supplementation and fraction of inspired oxygen) and a decreased likelihood of developing pneumonia but only in women, leading the authors to hypothesize that this protective effect is estrogen-dependent and mediated by the ER binding sites within the repeated monomer (237). Since our in-silico analysis only identified ER binding sites outside of the monomer, another possibility is an enhanced interaction between the ER and the increased number of transcription factors binding within the two monomers of allele *2. (**Figure 7**). In regard to external factors that influence B-cell function, the AhR may play an important environmental sensor role because of the diverse array of ligands produced from the diet and microbiota and even light exposure (72, 84, 96, 105, 108, 113–115). Rannug (270) proposed a diurnal oscillation in the concentration of AhR ligands, which is particularly interesting when considering that the AhR is part of a family of circadian rhythm proteins (271). The breakdown of tryptophan in the skin by sunlight exposure to the AhR ligand FICZ may be an important factor in skin homeostasis through a balance between immune suppression (high FICZ concentrations) and inflammation (low FICZ concentrations) (270). This is further supported by the improvement of psoriasis with sun exposure and the effectiveness of the AhR ligand tapinarof in resolving mild-to-severe plague psoriasis in Phase 3 trials (272). In conclusion, varying intrinsic and extrinsic signals could influence the overall transcription factor binding profiles and transcriptional control of the 3′RR and therefore antibody production, SHM, and CSR. As discussed above, evidence from mouse models supports the sensitivity of the 3′RR and antibody production to environmental chemicals (**Table 3**). However, the significant differences in the *IgH* gene between rodents and humans likely translates to functional differences as supported by the studies evaluating hs1.2 enhancer activity. Given the importance of antibodies in defense and disease, this represents a significant knowledge gap in human B-cell function. Based on the evidence to date, we propose that the genetic polymorphisms (i.e. hs1.2 alleles and 3′RR haplotypes) in human evolved to adapt to internal stimuli and changing environmental conditions, and that xenobiotics, such as chemicals in pollution and forest fires, and disease conditions significantly disrupt 3′RR activity by high jacking its environmental sensing function and tipping the balance towards a pathological response. Understanding genetic differences and the signaling pathways that converge at the 3′RR will provide valuable insight into individual sensitivities to environmental factors and antibody-mediated disease conditions.

## Author contributions

CS and DF contributed to conception and design of the review. CS and DF wrote the first draft of the manuscript. PD performed the in silico analysis. CS and PA contributed figures and figure legends. CS finalized the manuscript for submission. All authors contributed to the article and approved the submitted version.

## Supplementary material

Supplementary Figure 1 Chromosomal location of the segmental features of the human *IgH* gene. The top image represents the ideogram of human chromosome 14. Dark boxes depict the approximate location of bands, as seen on the Giemsa-stained chromosome. The constriction site (brown triangles) indicates the centromeric region, thus the p arm is on the right and the longer q arm on the left of the ideogram. The red box highlights the 14q32.33 band harboring the *IgH* region that is annotated in detail in the lower part of the figure. The gene annotation of the *IgH* region comes from the V39 release of GENCODE on the hg38 assembly of the human genome. The faded background colors highlight the different size of the genomic areas hosting V, D, J or C regions. Enhancers of the whole *IgH* region are also positioned, as reported in GeneHancer database, but we integrated these data with the position of the well-known enhancers belonging to the human 3’RRs.

File S2 Unabridged consensus transcription factor binding sites within the *4 allele of the hs1.2 enhancer. Allele *4 of the human hs1.2 enhancer is depicted as a detailed overview of the schema in . A general schematic of allele *4 is represented on page 1. A “core” (magenta) and a “tail” (light blue) sequence are common to all the hs1.2 allelic variants. The core sequence is also highly conserved in vertebrates. The “repeat” (yellow box) may be present from one to four times in human (see **Figure 7**) (alleles with more copies were found in primates). The allele *4 was analyzed in silico for potential transcription factor binding sites using TFBIND (tfbind.hgc.jp) and the results were depicted using SnapGene Viewer (https://www.snapgene.com). One transcription factor is reported on each page, starting from page 2. Codes refer to the sequence consensus motif in each transcription factor binding site, as listed in the “Gene Set Enrichment Analysis” database (GSEA - https://www.gsea-msigdb.org/gsea/msigdb/genesets.jsp).

## References

[1] Florescu DF Kalil AC Qiu F Schmidt CM Sandkovsky U. What is the impact of hypogammaglobulinemia on the rate of infections and survival in solid organ transplantation? A meta-analysis. Am J Transplant (2013) 13(10):2601–10. doi: 10.1111/ajt.12401 23919557

[2] Sanchez LA Maggadottir SM Pantell MS Lugar P Rundles CC Sullivan KE. Two sides of the same coin: Pediatric-onset and adult-onset common variable immune deficiency. J Clin Immunol (2017) 37(6):592–602. doi: 10.1007/s10875-017-0415-5 28755066

[3] Carbone J. The immunology of posttransplant CMV infection: Potential effect of CMV immunoglobulins on distinct components of the immune response to CMV. Transplantation (2016) 100 Suppl 3:S11–8. doi: 10.1097/TP.0000000000001095 PMC4764014 26900990

[4] Luebke RW Parks C Luster MI. Suppression of immune function and susceptibility to infections in humans: association of immune function with clinical disease. J Immunotoxicol (2004) 1(1):15–24. doi: 10.1080/15476910490438342 18958637

[5] Edwards JC Cambridge G. B-cell targeting in rheumatoid arthritis and other autoimmune diseases. Nat Rev Immunol (2006) 6(5):394–403. doi: 10.1038/nri1838 16622478

[6] Pers JO Daridon C Bendaoud B Devauchelle V Berthou C Saraux A. B-cell depletion and repopulation in autoimmune diseases. Clin Rev Allergy Immunol (2008) 34(1):50–5. doi: 10.1007/s12016-007-8015-4 18270858

[7] Ludwig RJ Vanhoorelbeke K Leypoldt F Kaya Z Bieber K McLachlan SM. Mechanisms of autoantibody-induced pathology. Front Immunol (2017) 8:603. doi: 10.3389/fimmu.2017.00603 28620373 PMC5449453

[8] Vidarsson G Dekkers G Rispens T. IgG subclasses and allotypes: from structure to effector functions. Front Immunol (2014) 5:520. doi: 10.3389/fimmu.2014.00520 25368619 PMC4202688

[9] Gadermaier E Levin M Flicker S Ohlin M. The human IgE repertoire. Int Arch Allergy Immunol (2014) 163(2):77–91. doi: 10.1159/000355947 24296690 PMC4497803

[10] Blutt SE Conner ME. The gastrointestinal frontier: IgA and viruses. Front Immunol (2013) 4:402. doi: 10.3389/fimmu.2013.00402 24348474 PMC3842584

[11] Horton RE Vidarsson G. Antibodies and their receptors: different potential roles in mucosal defense. Front Immunol (2013) 4:200. doi: 10.3389/fimmu.2013.00200 23882268 PMC3712224

[12] DeWitt JC Germolec DR Luebke RW Johnson VJ. Associating changes in the immune system with clinical diseases for interpretation in risk assessment. Curr Protoc Toxicol (2016) 67:18.1.1–18.1.22. doi: 10.1002/0471140856.tx1801s67 PMC4780336 26828330

[13] Winans B Humble MC Lawrence BP. Environmental toxicants and the developing immune system: a missing link in the global battle against infectious disease? Reprod Toxicol (2011) 31(3):327–36. doi: 10.1016/j.reprotox.2010.09.004 PMC3033466 20851760

[14] Franchini AM Lawrence BP. Environmental exposures are hidden modifiers of anti-viral immunity. Curr Opin Toxicol (2018) 10:54–9. doi: 10.1016/j.cotox.2018.01.004 PMC6051538 30035244

[15] Boverhof DR Ladics G Luebke B Botham J Corsini E Evans E. Approaches and considerations for the assessment of immunotoxicity for environmental chemicals: a workshop summary. Regul Toxicol Pharmacol (2014) 68(1):96–107. doi: 10.1016/j.yrtph.2013.11.012 24280359

[16] White SS Birnbaum LS. An overview of the effects of dioxins and dioxin-like compounds on vertebrates, as documented in human and ecological epidemiology. J Environ Sci Health C Environ Carcinog Ecotoxicol Rev (2009) 27(4):197–211. doi: 10.1080/10590500903310047 19953395 PMC2788749

[17] Zhou J Henriquez J Crawford R Kaminski N. Suppression of the IgM response by aryl hydrocarbon receptor activation in human primary b cells involves impairment of immunoglobulin secretory processes. Toxicol Sci (2018) 163(1):319–29. doi: 10.1093/toxsci/kfy036 PMC6659029 29462421

[18] Yoo BS Boverhof DR Shnaider D Crawford RB Zacharewski TR Kaminski NE. 2,3,7,8-tetrachlorodibenzo-p-dioxin (TCDD) alters the regulation of Pax5 in lipopolysaccharide-activated b cells. Toxicol Sci (2004) 77(2):272–9. doi: 10.1093/toxsci/kfh013 14600275

[19] Fernando TM Ochs SD Liu J Chambers-Turner RC Sulentic CEW. 2,3,7,8-Tetrachlorodibenzo-p-Dioxin induces transcriptional activity of the human polymorphic hs1,2 enhancer of the 3'Igh regulatory region. J Immunol (2012) 188(7):3294–306. doi: 10.4049/jimmunol.1101111 PMC3311717 22357631

[20] Suh J Kang JS Yang KH Kaminski NE. Antagonism of aryl hydrocarbon receptor-dependent induction of CYP1A1 and inhibition of IgM expression by di-ortho-substituted polychlorinated biphenyls. Toxicol Appl Pharmacol (2003) 187(1):11–21. doi: 10.1016/S0041-008X(02)00040-6 12628580

[21] Vorderstrasse BA Steppan LB Silverstone AE Kerkvliet NI. Aryl hydrocarbon receptor-deficient mice generate normal immune responses to model antigens and are resistant to TCDD-induced immune suppression. Toxicol Appl Pharmacol (2001) 171(3):157–64. doi: 10.1006/taap.2000.9122 11243915

[22] Harper N Connor K Steinberg M Safe S. An enzyme-linked immunosorbent assay (ELISA) specific for antibodies to TNP-LPS detects alterations in serum immunoglobulins and isotype switching in C57BL/6 and DBA/2 mice exposed to 2,3,7,8-tetrachlorodibenzo-p-dioxin and related compounds. Toxicology (1994) 92(1-3):155–67. doi: 10.1016/0300-483X(94)90174-0 7940557

[23] Tucker AN Vore SJ Luster MI. Suppression of b cell differentiation by 2,3,7,8-tetrachlorodibenzo-p-dioxin. Mol Pharmacol (1986) 29(4):372–7. 3486342

[24] Dooley RK Holsapple MP. Elucidation of cellular targets responsible for tetrachlorodibenzo-p-dioxin (TCDD)-induced suppression of antibody responses: I. role B lymphocyte. Immunopharmacol (1988) 16(3):167–80. doi: 10.1016/0162-3109(88)90005-7 3267010

[25] Luster MI Germolec DR Clark G Wiegand G Rosenthal GJ. Selective effects of 2,3,7,8-tetrachlorodibenzo-p-dioxin and corticosteroid on *in vitro* lymphocyte maturation. J Immunol (1988) 140(3):928–35. doi: 10.4049/jimmunol.140.3.928 3257509

[26] Holsapple MP Dooley RK McNerney PJ McCay JA. Direct suppression of antibody responses by chlorinated dibenzodioxins in cultured spleen cells from (C57BL/6 x C3H)F1 and DBA/2 mice. Immunopharmacology (1986) 12(3):175–86. doi: 10.1016/0162-3109(86)90001-9 3818259

[27] Lawrence BP Vorderstrasse BA. Activation of the aryl hydrocarbon receptor diminishes the memory response to homotypic influenza virus infection but does not impair host resistance. Toxicol Sci (2004) 79(2):304–14. doi: 10.1093/toxsci/kfh094 14976337

[28] Allan LL Sherr DH. Disruption of human plasma cell differentiation by an environmental polycyclic aromatic hydrocarbon: a mechanistic immunotoxicological study. Environ Health (2010) 9:15. doi: 10.1186/1476-069X-9-15 20334656 PMC2851679

[29] Morris DL Karras JG Holsapple MP. Direct effects of 2,3,7,8-tetrachlorodibenzo-p-dioxin (TCDD) on responses to lipopolysaccharide (LPS) by isolated murine b-cells. Immunopharmacology (1993) 26(2):105–12. doi: 10.1016/0162-3109(93)90002-8 8282534

[30] Dornbos P Warren M Crawford RB Kaminski NE Threadgill DW LaPres JJ. Characterizing Serpinb2 as a modulator of TCDD-induced suppression of the b cell. Chem Res Toxicol (2018) 31(11):1248–59. doi: 10.1021/acs.chemrestox.8b00225 PMC7234800 30339366

[31] Schneider D Manzan MA Crawford RB Chen W Kaminski NE. 2,3,7,8-tetrachlorodibenzo-p-dioxin-mediated impairment of b cell differentiation involves dysregulation of paired box 5 (Pax5) isoform, Pax5a. J Pharmacol Exp Ther (2008) 326(2):463–74. doi: 10.1124/jpet.108.139857 PMC2562000 18483191

[32] Sulentic CEW Holsapple MP Kaminski NE. Aryl hydrocarbon receptor-dependent suppression by 2,3,7, 8-tetrachlorodibenzo-p-dioxin of IgM secretion in activated b cells. Mol Pharmacol (1998) 53(4):623–9. doi: 10.1124/mol.53.4.623 9547351

[33] Wourms MJ Sulentic CE. The aryl hydrocarbon receptor regulates an essential transcriptional element in the immunoglobulin heavy chain gene. Cell Immunol (2015) 295(1):60–6. doi: 10.1016/j.cellimm.2015.02.012 PMC4427249 25749007

[34] Sulentic CEW Holsapple MP Kaminski NE. Putative link between transcriptional regulation of IgM expression by 2,3,7,8-tetrachlorodibenzo-p-dioxin and the aryl hydrocarbon receptor/dioxin-responsive enhancer signaling pathway. J Pharmacol Exp Ther (2000) 295(2):705–16. 11046109

[35] Phadnis-Moghe AS Chen W Li J Crawford RB Bach A D'Ingillo S. Immunological characterization of the aryl hydrocarbon receptor (AHR) knockout rat in the presence and absence of 2,3,7,8-tetrachlorodibenzo-p-dioxin (TCDD). Toxicology (2016) 368-369:172–82. doi: 10.1016/j.tox.2016.08.019 27590929

[36] Yoshida T Katsuya K Oka T Koizumi S Wakita D Kitamura H. Effects of AhR ligands on the production of immunoglobulins in purified mouse b cells. BioMed Res (2012) 33(2):67–74. doi: 10.2220/biomedres.33.67 22572380

[37] Ishikawa S. Children's immunology, what can we learn from animal studies (3): Impaired mucosal immunity in the gut by 2,3,7,8-tetraclorodibenzo-p-dioxin (TCDD): a possible role for allergic sensitization. J toxicological Sci (2009) 34 Suppl 2:SP349–61. doi: 10.2131/jts.34.SP349 19571490

[38] Wood SC Jeong HG Morris DL Holsapple MP. Direct effects of 2,3,7,8-tetrachlorodibenzo-p-dioxin (TCDD) on human tonsillar lymphocytes. Toxicology (1993) 81(2):131–43. doi: 10.1016/0300-483X(93)90005-D 8378939

[39] Lu H Crawford RB Suarez-Martinez JE Kaplan BL Kaminski NE. Induction of the aryl hydrocarbon receptor-responsive genes and modulation of the immunoglobulin m response by 2,3,7,8-tetrachlorodibenzo-p-dioxin in primary human b cells. Toxicol Sci (2010) 118(1):86–97. doi: 10.1093/toxsci/kfq234 20702590 PMC2955211

[40] Dornbos P Crawford RB Kaminski NE Hession SL LaPres JJ. The influence of human interindividual variability on the low-dose region of dose-response curve induced by 2,3,7,8-Tetrachlorodibenzo-p-Dioxin in primary b cells. Toxicol Sci (2016) 153(2):352–60. doi: 10.1093/toxsci/kfw128 PMC5036619 27473338

[41] Zhou J Zhang Q Henriquez JE Crawford RB Kaminski NE. Lymphocyte-specific protein tyrosine kinase (LCK) is involved in the aryl hydrocarbon receptor-mediated impairment of immunoglobulin secretion in human primary b cells. Toxicol Sci (2018) 165(2):322–34. doi: 10.1093/toxsci/kfy133 PMC6659013 29860352

[42] Blevins LK Zhou J Crawford RB Kaminski NE. Identification of a sensitive human immunological target of aryl hydrocarbon receptor activation: CD5(+) innate-like b cells. Front Immunol (2021) 12:635748. doi: 10.3389/fimmu.2021.635748 33936048 PMC8082145

[43] Kovalova N Manzan M Crawford R Kaminski N. Role of aryl hydrocarbon receptor polymorphisms on TCDD-mediated CYP1B1 induction and IgM suppression by human b cells. Toxicol Appl Pharmacol (2016) 309:15–23. doi: 10.1016/j.taap.2016.08.011 27535091 PMC5035641

[44] Lu YC Wu YC. Clinical findings and immunological abnormalities in yu-Cheng patients. Environ Health Perspect (1985) 59:17–29. doi: 10.2307/3429869 3921359 PMC1568085

[45] Nakanishi Y Shigematsu N Kurita Y Matsuba K Kanegae H Ishimaru S. Respiratory involvement and immune status in yusho patients. Environ Health Perspect (1985) 59:31–6. doi: 10.1289/ehp.59-1568074 PMC1568074 3921360

[46] Baccarelli A Mocarelli P Patterson DG Jr. Bonzini M Pesatori AC Caporaso N. Immunologic effects of dioxin: new results from seveso and comparison with other studies. Environ Health Perspect (2002) 110(12):1169–73. doi: 10.1289/ehp.021101169 PMC1241102 12460794

[47] Kimata H. 2,3,7,8-tetrachlorodibenzo-p-dioxin selectively enhances spontaneous IgE production in b cells from atopic patients. Int J Hyg Environ Health (2003) 206(6):601–4. doi: 10.1078/1438-4639-00248 14626908

[48] Kummari E Rushing E Nicaise A McDonald A Kaplan BLF. TCDD attenuates EAE through induction of FasL on b cells and inhibition of IgG production. Toxicology (2021) 448:152646. doi: 10.1016/j.tox.2020.152646 33253778 PMC7785643

[49] Nicaise AJ McDonald A Sears ER Sturgis T Kaplan BLF. TCDD inhibition of IgG1 production in experimental autoimmune encephalomyelitis (EAE) and *In vitro* . Antibodies (Basel) (2022) 11(1):4–19. doi: 10.3390/antib11010004 PMC8788515 35076460

[50] Chmill S Kadow S Winter M Weighardt H Esser C. 2,3,7,8-tetrachlorodibenzo-p-dioxin impairs stable establishment of oral tolerance in mice. Toxicol Sci (2010) 118(1):98–107. doi: 10.1093/toxsci/kfq232 20729464

[51] Vaidyanathan B Chaudhry A Yewdell WT Angeletti D Yen WF Wheatley AK. The aryl hydrocarbon receptor controls cell-fate decisions in b cells. J Exp Med (2017) 214(1):197–208. doi: 10.1084/jem.20160789 28011866 PMC5206498

[52] Kim HA Kim EM Park YC Yu JY Hong SK Jeon SH. Immunotoxicological effects of agent orange exposure to the Vietnam war Korean veterans. Ind Health (2003) 41(3):158–66. doi: 10.2486/indhealth.41.158 12916745

[53] Kinoshita H Abe J Akadegawa K Yurino H Uchida T Ikeda S. Breakdown of mucosal immunity in gut by 2,3,7,8-tetraclorodibenzo-p-dioxin (TCDD). Environ Health Prev Med (2006) 11(5):256–63. doi: 10.1007/BF02898015 PMC2723348 21432354

[54] Foxx CL Nagy MR King AE Albin D DeKrey GK. TCDD exposure alters fecal IgA concentrations in male and female mice. BMC Pharmacol Toxicol (2022) 23(1):25. doi: 10.1186/s40360-022-00563-9 35449084 PMC9026712

[55] Zhang L Nichols RG Correll J Murray IA Tanaka N Smith PB. Persistent organic pollutants modify gut microbiota-host metabolic homeostasis in mice through aryl hydrocarbon receptor activation. Environ Health Perspect (2015) 123(7):679–88. doi: 10.1289/ehp.1409055 PMC4492271 25768209

[56] Benson JM Shepherd DM. Aryl hydrocarbon receptor activation by TCDD reduces inflammation associated with crohn's disease. Toxicol Sci (2011) 120(1):68–78. doi: 10.1093/toxsci/kfq360 21131560 PMC3044199

[57] Henseler RA Romer EJ Sulentic CEW. Diverse chemicals including aryl hydrocarbon receptor ligands modulate transcriptional activity of the 3'immunoglobulin heavy chain regulatory region. Toxicology (2009) 261(1-2):9–18. doi: 10.1016/j.tox.2009.03.015 19447539 PMC2692577

[58] Takenaka H Zhang K Diaz-Sanchez D Tsien A Saxon A. Enhanced human IgE production results from exposure to the aromatic hydrocarbons from diesel exhaust: direct effects on b-cell IgE production. J Allergy Clin Immunol (1995) 95:103–15. doi: 10.1016/S0091-6749(95)70158-3 7529782

[59] Heilmann C Grandjean P Weihe P Nielsen F Budtz-Jorgensen E. Reduced antibody responses to vaccinations in children exposed to polychlorinated biphenyls. PloS Med (2006) 3(8):e311. doi: 10.1371/journal.pmed.0030311 16942395 PMC1551916

[60] Heilmann C Budtz-Jorgensen E Nielsen F Heinzow B Weihe P Grandjean P. Serum concentrations of antibodies against vaccine toxoids in children exposed perinatally to immunotoxicants. Environ Health Perspect (2010) 118(10):1434–8. doi: 10.1289/ehp.1001975 PMC2957925 20562056

[61] Stolevik SB Nygaard UC Namork E Haugen M Meltzer HM Alexander J. Prenatal exposure to polychlorinated biphenyls and dioxins from the maternal diet may be associated with immunosuppressive effects that persist into early childhood. Food Chem Toxicol (2013) 51:165–72. doi: 10.1016/j.fct.2012.09.027 23036451

[62] Jusko TA De Roos AJ Lee SY Thevenet-Morrison K Schwartz SM Verner MA. A birth cohort study of maternal and infant serum PCB-153 and DDE concentrations and responses to infant tuberculosis vaccination. Environ Health Perspect (2016) 124(6):813–21. doi: 10.1289/ehp.1510101 PMC4892928 26649893

[63] Thatcher TH Williams MA Pollock SJ McCarthy CE Lacy SH Phipps RP. Endogenous ligands of the aryl hydrocarbon receptor regulate lung dendritic cell function. Immunology (2016) 147(1):41–54. doi: 10.1111/imm.12540 26555456 PMC4693882

[64] Crawford RB Holsapple MP Kaminski NE. Leukocyte activation induces aryl hydrocarbon receptor up-regulation, DNA binding, and increased Cyp1a1 expression in the absence of exogenous ligand. Mol Pharmacol (1997) 52(6):921–7. doi: 10.1124/mol.52.6.921 9415701

[65] Marcus RS Holsapple MP Kaminski NE. Lipopolysaccharide activation of murine splenocytes and splenic b cells increased the expression of aryl hydrocarbon receptor and aryl hydrocarbon receptor nuclear translocator. J Pharmacol Exp Ther (1998) 287(3):1113–8. 9864300

[66] Sherr DH Monti S. The role of the aryl hydrocarbon receptor in normal and malignant b cell development. Semin Immunopathol (2013) 35(6):705–16. doi: 10.1007/s00281-013-0390-8 PMC3824572 23942720

[67] Allan LL Sherr DH. Constitutive activation and environmental chemical induction of the aryl hydrocarbon receptor/transcription factor in activated human b lymphocytes. Mol Pharmacol (2005) 67(5):1740–50. doi: 10.1124/mol.104.009100 15681594

[68] Tanaka G Kanaji S Hirano A Arima K Shinagawa A Goda C. Induction and activation of the aryl hydrocarbon receptor by IL-4 in b cells. Int Immunol (2005) 17(6):797–805. doi: 10.1093/intimm/dxh260 15899923

[69] Ovesen JL Schnekenburger M Puga A. Aryl hydrocarbon receptor ligands of widely different toxic equivalency factors induce similar histone marks in target gene chromatin. Toxicol Sci (2011) 121(1):123–31. doi: 10.1093/toxsci/kfr032 PMC3080189 21292640

[70] Hubbard TD Murray IA Perdew GH. Indole and tryptophan metabolism: Endogenous and dietary routes to ah receptor activation. Drug Metab Dispos (2015) 43(10):1522–35. doi: 10.1124/dmd.115.064246 PMC4576673 26041783

[71] Denison MS Nagy SR. Activation of the aryl hydrocarbon receptor by structurally diverse exogenous and endogenous chemicals. Annu Rev Pharmacol Toxicol (2003) 43:309–34. doi: 10.1146/annurev.pharmtox.43.100901.135828 12540743

[72] Hubbard TD Murray IA Bisson WH Lahoti TS Gowda K Amin SG. Adaptation of the human aryl hydrocarbon receptor to sense microbiota-derived indoles. Sci Rep (2015) 5:12689. doi: 10.1038/srep12689 26235394 PMC4522678

[73] Murray IA Patterson AD Perdew GH. Aryl hydrocarbon receptor ligands in cancer: friend and foe. Nat reviews.Cancer (2014) 14(12):801–14. doi: 10.1038/nrc3846 PMC4401080 25568920

[74] Lawrence BP Vorderstrasse BA. New insights into the aryl hydrocarbon receptor as a modulator of host responses to infection. Semin Immunopathol (2013) 35(6):615–26. doi: 10.1007/s00281-013-0395-3 PMC3808126 23963494

[75] Lawrence BP Sherr DH. You AhR what you eat? Nat Immunol (2012) 13(2):117–9. doi: 10.1038/ni.2213 22261961

[76] Murray IA Krishnegowda G DiNatale BC Flaveny C Chiaro C Lin JM. Development of a selective modulator of aryl hydrocarbon (Ah) receptor activity that exhibits anti-inflammatory properties. Chem Res Toxicol (2010) 23(5):955–66. doi: 10.1021/tx100045h PMC2871980 20423157

[77] Marafini I Di Fusco D Dinallo V Franze E Stolfi C Sica G. NPD-0414-2 and NPD-0414-24, two chemical entities designed as aryl hydrocarbon receptor (AhR) ligands, inhibit gut inflammatory signals. Front Pharmacol (2019) 10:380. doi: 10.3389/fphar.2019.00380 31031628 PMC6473199

[78] Esser C. Trajectory shifts in interdisciplinary research of the aryl hydrocarbon receptor-a personal perspective on thymus and skin. Int J Mol Sci (2021) 22(4):1844–1858. doi: 10.3390/ijms22041844 PMC7918350 33673338

[79] Lebwohl MG Stein Gold L Strober B Papp KA Armstrong AW Bagel J. Phase 3 trials of tapinarof cream for plaque psoriasis. N Engl J Med (2021) 385(24):2219–29. doi: 10.1056/NEJMoa2103629 34879448

[80] Sepulveda MA Garrett FE Price-Whelan A Birshtein BK. Comparative analysis of human and mouse 3' igh regulatory regions identifies distinctive structural features. Mol Immunol (2005) 42(5):605–15. doi: 10.1016/j.molimm.2004.09.006 15607820

[81] Vazquez-Rivera E Rojas B Parrott JC Shen AL Xing Y Carney PR. The aryl hydrocarbon receptor as a model PAS sensor. Toxicol Rep (2022) 9:1–11. doi: 10.1016/j.toxrep.2021.11.017 34950569 PMC8671103

[82] Okey AB. An aryl hydrocarbon receptor odyssey to the shores of toxicology: the deichmann lecture, international congress of toxicology-XI. Toxicol Sci (2007) 98(1):5–38. doi: 10.1093/toxsci/kfm096 17569696

[83] Abel J Haarmann-Stemmann T. An introduction to the molecular basics of aryl hydrocarbon receptor biology. Biol Chem (2010) 391(11):1235–48. doi: 10.1515/bc.2010.128 20868221

[84] Rothhammer V Quintana FJ. The aryl hydrocarbon receptor: an environmental sensor integrating immune responses in health and disease. Nat Rev Immunol (2019) 19(3):184–97. doi: 10.1038/s41577-019-0125-8 30718831

[85] Roman AC Carvajal-Gonzalez JM Merino JM Mulero-Navarro S Fernandez-Salguero PM. The aryl hydrocarbon receptor in the crossroad of signalling networks with therapeutic value. Pharmacol Ther (2018) 185:50–63. doi: 10.1016/j.pharmthera.2017.12.003 29258844

[86] Wang B Zhou Z Li L. Gut microbiota regulation of AHR signaling in liver disease. Biomolecules (2022) 12(9):1244–1259. doi: 10.3390/biom12091244 PMC9496174 36139083

[87] McMillan BJ Bradfield CA. The aryl hydrocarbon receptor sans xenobiotics: endogenous function in genetic model systems. Mol Pharmacol (2007) 72(3):487–98. doi: 10.1124/mol.107.037259 17535977

[88] Nguyen LP Bradfield CA. The search for endogenous activators of the aryl hydrocarbon receptor. Chem Res Toxicol (2007) 21(1):102–16. doi: 10.1021/tx7001965 PMC2572005 18076143

[89] Vogel CF Sciullo E Wong P Kuzmicky P Kado N Matsumura F. Induction of proinflammatory cytokines and c-reactive protein in human macrophage cell line U937 exposed to air pollution particulates. Environ Health Perspect (2005) 113(11):1536–41. doi: 10.1289/ehp.8094 PMC1310915 16263508

[90] Cheon H Woo YS Lee JY Kim HS Kim HJ Cho S. Signaling pathway for 2,3,7,8-tetrachlorodibenzo-p-dioxin-induced TNF-alpha production in differentiated THP-1 human macrophages. Exp Mol Med (2007) 39(4):524–34. doi: 10.1038/emm.2007.58 17934341

[91] Sekine H Mimura J Oshima M Okawa H Kanno J Igarashi K. Hypersensitivity of aryl hydrocarbon receptor-deficient mice to lipopolysaccharide-induced septic shock. Mol Cell Biol (2009) 29(24):6391–400. doi: 10.1128/MCB.00337-09 PMC2786870 19822660

[92] Kimura A Naka T Nakahama T Chinen I Masuda K Nohara K. Aryl hydrocarbon receptor in combination with Stat1 regulates LPS-induced inflammatory responses. J Exp Med (2009) 206(9):2027–35. doi: 10.1084/jem.20090560 PMC2737163 19703987

[93] Bankoti J Rase B Simones T Shepherd DM. Functional and phenotypic effects of AhR activation in inflammatory dendritic cells. Toxicol Appl Pharmacol (2010) 246(1-2):18–28. doi: 10.1016/j.taap.2010.03.013 20350561 PMC2885531

[94] Fan Y Boivin GP Knudsen ES Nebert DW Xia Y Puga A. The aryl hydrocarbon receptor functions as a tumor suppressor of liver carcinogenesis. Cancer Res (2010) 70(1):212–20. doi: 10.1158/0008-5472.CAN-09-3090 PMC2939500 19996281

[95] de Souza AR Zago M Eidelman DH Hamid Q Baglole CJ. Aryl hydrocarbon receptor (AhR) attenuation of subchronic cigarette smoke-induced pulmonary neutrophilia is associated with retention of nuclear RelB and suppression of intercellular adhesion molecule-1 (ICAM-1). Toxicological Sci an Off J Soc Toxicol (2014) 140(1):204–23. doi: 10.1093/toxsci/kfu068 24752502

[96] Bessede A Gargaro M Pallotta MT Matino D Servillo G Brunacci C. Aryl hydrocarbon receptor control of a disease tolerance defence pathway. Nature (2014) 511(7508):184–90. doi: 10.1038/nature13323 PMC4098076 24930766

[97] Lee YH Lin CH Hsu PC Sun YY Huang YJ Zhuo JH. Aryl hydrocarbon receptor mediates both proinflammatory and anti-inflammatory effects in lipopolysaccharide-activated microglia. Glia (2015) 63(7):1138–54. doi: 10.1002/glia.22805 25690886

[98] Sulentic CEW Snyder AD Salisbury RL. The aryl hydrocarbon receptor and immunity. In: McQueen CA, editor. Comprehensive toxicology, 3rd ed, vol. 11 . Oxford: Elsevier Ltd (2018). p. 238–71.

[99] Butler RA Kelley ML Powell WH Hahn ME Van Beneden RJ. An aryl hydrocarbon receptor (AHR) homologue from the soft-shell clam, mya arenaria: evidence that invertebrate AHR homologues lack 2,3,7,8-tetrachlorodibenzo-p-dioxin and beta-naphthoflavone binding. Gene (2001) 278(1-2):223–34. doi: 10.1016/S0378-1119(01)00724-7 11707340

[100] Powell-Coffman JA Bradfield CA Wood WB. Caenorhabditis elegans orthologs of the aryl hydrocarbon receptor and its heterodimerization partner the aryl hydrocarbon receptor nuclear translocator. Proc Natl Acad Sci USA (1998) 95(6):2844–9. doi: 10.1073/pnas.95.6.2844 PMC19657 9501178

[101] Hahn ME Karchner SI Shapiro MA Perera SA. Molecular evolution of two vertebrate aryl hydrocarbon (dioxin) receptors (AHR1 and AHR2) and the PAS family. Proc Natl Acad Sci U.S.A. (1997) 94(25):13743–8. doi: 10.1073/pnas.94.25.13743 PMC28377 9391097

[102] Hahn ME Karchner SI Evans BR Franks DG Merson RR Lapseritis JM. Unexpected diversity of aryl hydrocarbon receptors in non-mammalian vertebrates: insights from comparative genomics. J Exp Zool A Comp Exp Biol (2006) 305(9):693–706. doi: 10.1002/jez.a.323 16902966

[103] Nagy SR Liu G Lam KS Denison MS. Identification of novel ah receptor agonists using a high-throughput green fluorescent protein-based recombinant cell bioassay. Biochemistry (2002) 41(3):861–8. doi: 10.1021/bi011373v 11790108

[104] Laub LB Jones BD Powell WH. Responsiveness of a xenopus laevis cell line to the aryl hydrocarbon receptor ligands 6-formylindolo[3,2-b]carbazole (FICZ) and 2,3,7,8-tetrachlorodibenzo-p-dioxin (TCDD). Chem Biol Interact (2010) 183(1):202–11. doi: 10.1016/j.cbi.2009.09.017 PMC2795045 19799885

[105] Faber SC Soshilov AA Giani Tagliabue S Bonati L Denison MS. Comparative *In vitro* and in silico analysis of the selectivity of indirubin as a human ah receptor agonist. Int J Mol Sci (2018) 19(9):2692–2709. doi: 10.3390/ijms19092692 PMC6165432 30201897

[106] Ema M Ohe N Suzuki M Mimura J Sogawa K Ikawa S. Dioxin binding activities of polymorphic forms of mouse and human arylhydrocarbon receptors. J Biol Chem (1994) 269(44):27337–43. doi: 10.1016/S0021-9258(18)46990-6 7961644

[107] Ramadoss P Perdew GH. Use of 2-azido-3-[125I]iodo-7,8-dibromodibenzo-p-dioxin as a probe to determine the relative ligand affinity of human versus mouse aryl hydrocarbon receptor in cultured cells. Mol Pharmacol (2004) 66(1):129–36. doi: 10.1124/mol.66.1.129 15213304

[108] Doan TQ Connolly L Igout A Muller M Scippo ML. *In vitro* differential responses of rat and human aryl hydrocarbon receptor to two distinct ligands and to different polyphenols. Environ pollut (2020) 265(Pt B):114966. doi: 10.1016/j.envpol.2020.114966 32563119

[109] Flaveny C Reen RK Kusnadi A Perdew GH. The mouse and human ah receptor differ in recognition of LXXLL motifs. Arch Biochem Biophys (2008) 471(2):215–23. doi: 10.1016/j.abb.2008.01.014 PMC2293825 18242161

[110] Flaveny CA Murray IA Perdew GH. Differential gene regulation by the human and mouse aryl hydrocarbon receptor. Toxicol Sci (2010) 114(2):217–25. doi: 10.1093/toxsci/kfp308 PMC2840214 20044593

[111] Forgacs AL Dere E Angrish MM Zacharewski TR. Comparative analysis of temporal and dose-dependent TCDD-elicited gene expression in human, mouse, and rat primary hepatocytes. Toxicol Sci (2013) 133(1):54–66. doi: 10.1093/toxsci/kft028 23418086 PMC3627554

[112] Moriguchi T Motohashi H Hosoya T Nakajima O Takahashi S Ohsako S. Distinct response to dioxin in an arylhydrocarbon receptor (AHR)-humanized mouse. Proc Natl Acad Sci U.S.A. (2003) 100(10):5652–7. doi: 10.1073/pnas.1037886100 PMC156256 12730383

[113] Adachi J Mori Y Matsui S Takigami H Fujino J Kitagawa H. Indirubin and indigo are potent aryl hydrocarbon receptor ligands present in human urine. J Biol Chem (2001) 276(34):31475–8. doi: 10.1074/jbc.C100238200 11425848

[114] Rannug A Rannug U. The tryptophan derivative 6-formylindolo[3,2-b]carbazole, FICZ, a dynamic mediator of endogenous aryl hydrocarbon receptor signaling, balances cell growth and differentiation. Crit Rev Toxicol (2018) 48(7):555–74. doi: 10.1080/10408444.2018.1493086 30226107

[115] Denison MS Soshilov AA He G DeGroot DE Zhao B. Exactly the same but different: promiscuity and diversity in the molecular mechanisms of action of the aryl hydrocarbon (dioxin) receptor. Toxicol Sci (2011) 124(1):1–22. doi: 10.1093/toxsci/kfr218 21908767 PMC3196658

[116] Cohen LB Troemel ER. Microbial pathogenesis and host defense in the nematode c. elegans. Curr Opin Microbiol (2015) 23:94–101. doi: 10.1016/j.mib.2014.11.009 25461579 PMC4324121

[117] Tecle E Chhan CB Franklin L Underwood RS Hanna-Rose W Troemel ER. The purine nucleoside phosphorylase pnp-1 regulates epithelial cell resistance to infection in c. elegans. PloS Pathog (2021) 17(4):e1009350. doi: 10.1371/journal.ppat.1009350 33878133 PMC8087013

[118] Rannug A. How the AHR became important in intestinal homeostasis-a diurnal FICZ/AHR/CYP1A1 feedback controls both immunity and immunopathology. Int J Mol Sci (2020) 21(16):5681–5699. doi: 10.3390/ijms21165681 PMC7461111 32784381

[119] Gutierrez-Vazquez C Quintana FJ. Regulation of the immune response by the aryl hydrocarbon receptor. Immunity (2018) 48(1):19–33. doi: 10.1016/j.immuni.2017.12.012 29343438 PMC5777317

[120] Schroeder HW Jr. Cavacini L. Structure and function of immunoglobulins. J Allergy Clin Immunol (2010) 125(2 Suppl 2):S41–52. doi: 10.1016/j.jaci.2009.09.046 PMC3670108 20176268

[121] Buckley RH Orange JS. Primary immunodeficiency diseases. In: Burks AW, editor. Middleton’s allergy: Principles and practice, 9th ed. Elsevier, Amsterdam (2020). p. 1123–1152.e1.

[122] Boyle JM Buckley RH. Population prevalence of diagnosed primary immunodeficiency diseases in the united states. J Clin Immunol (2007) 27(5):497–502. doi: 10.1007/s10875-007-9103-1 17577648

[123] Lougaris V Pession A Baronio M Soresina A Rondelli R Gazzurelli L. The Italian registry for primary immunodeficiencies (Italian primary immunodeficiency network; IPINet): Twenty years of experience (1999-2019). J Clin Immunol (2020) 40(7):1026–37. doi: 10.1007/s10875-020-00844-0 PMC7505879 32803625

[124] Gathmann B Grimbacher B Beaute J Dudoit Y Mahlaoui N Fischer A. The European internet-based patient and research database for primary immunodeficiencies: results 2006-2008. Clin Exp Immunol (2009) 157 Suppl 1:3–11. doi: 10.1111/j.1365-2249.2009.03954.x 19630863 PMC2715433

[125] Torgerson TR. Immune dysregulation in primary immunodeficiency disorders. Immunol Allergy Clin North Am (2008) 28(2):315–27. doi: 10.1016/j.iac.2008.02.002 18424335

[126] Mayor PC Eng KH Singel KL Abrams SI Odunsi K Moysich KB. Cancer in primary immunodeficiency diseases: Cancer incidence in the united states immune deficiency network registry. J Allergy Clin Immunol (2018) 141(3):1028–35. doi: 10.1016/j.jaci.2017.05.024 PMC5723251 28606585

[127] Picard C Bobby Gaspar H Al-Herz W Bousfiha A Casanova JL Chatila T. International union of immunological societies: 2017 primary immunodeficiency diseases committee report on inborn errors of immunity. J Clin Immunol (2018) 38(1):96–128. doi: 10.1007/s10875-017-0464-9 29226302 PMC5742601

[128] Sulentic CEW Zhang W Na YJ Kaminski NE. 2,3,7,8-tetrachlorodibenzo-p-dioxin, an exogenous modulator of the 3'alpha immunoglobulin heavy chain enhancer in the CH12.LX mouse cell line. J Pharmacol Exp Ther (2004) 309(1):71–8. doi: 10.1124/jpet.103.059493 14718603

[129] Grasseau A Boudigou M Le Pottier L Chriti N Cornec D Pers JO. Innate b cells: the archetype of protective immune cells. Clin Rev Allergy Immunol (2020) 58(1):92–106. doi: 10.1007/s12016-019-08748-7 31183788

[130] Zhang Q Kline DE Bhattacharya S Crawford RB Conolly RB Thomas RS. All-or-none suppression of b cell terminal differentiation by environmental contaminant 2,3,7,8-tetrachlorodibenzo-p-dioxin. Toxicol Appl Pharmacol (2013) 268(1):17–26. doi: 10.1016/j.taap.2013.01.015 23357550 PMC3594464

[131] Schneider D Manzan MA Yoo BS Crawford RB Kaminski N. Involvement of blimp-1 and AP-1 dysregulation in the 2,3,7,8-tetrachlorodibenzo-p-dioxin-mediated suppression of the IgM response by b cells. Toxicol Sci (2009) 108(2):377–88. doi: 10.1093/toxsci/kfp028 PMC2664693 19237549

[132] De Abrew KN Kaminski NE Thomas RS. An integrated genomic analysis of aryl hydrocarbon receptor-mediated inhibition of b-cell differentiation. Toxicol Sci (2010) 118(2):454–69. doi: 10.1093/toxsci/kfq265 PMC3003543 20819909

[133] Phadnis-Moghe AS Crawford RB Kaminski NE. Suppression of human b cell activation by 2,3,7,8-tetrachlorodibenzo-p-dioxin involves altered regulation of b cell lymphoma-6. Toxicol Sci (2015) 144(1):39–50. doi: 10.1093/toxsci/kfu257 25543051 PMC4349138

[134] Phadnis-Moghe AS Li J Crawford RB Kaminski NE. SHP-1 is directly activated by the aryl hydrocarbon receptor and regulates BCL-6 in the presence of 2,3,7,8-tetrachlorodibenzo-p-dioxin (TCDD). Toxicol Appl Pharmacol (2016) 310:41–50. doi: 10.1016/j.taap.2016.08.014 27546522 PMC5064869

[135] Blevins LK Zhou J Crawford R Kaminski NE. TCDD-mediated suppression of naive human b cell IgM secretion involves aryl hydrocarbon receptor-mediated reduction in STAT3 serine 727 phosphorylation and is restored by interferon-gamma. Cell Signal (2020) 65:109447. doi: 10.1016/j.cellsig.2019.109447 31678681 PMC6903688

[136] Lu H Crawford RB Kaplan BL Kaminski NE. 2,3,7,8-tetrachlorodibenzo-p-dioxin-mediated disruption of the CD40 ligand-induced activation of primary human b cells. Toxicol Appl Pharmacol (2011) 255(3):251–60. doi: 10.1016/j.taap.2011.06.026 PMC3189629 21807014

[137] Kovalova N Nault R Crawford R Zacharewski TR Kaminski NE. Comparative analysis of TCDD-induced AhR-mediated gene expression in human, mouse and rat primary b cells. Toxicol Appl Pharmacol (2017) 316:95–106. doi: 10.1016/j.taap.2016.11.009 27913140 PMC5292778

[138] Ehrlich AK Pennington JM Bisson WH Kolluri SK Kerkvliet NI. TCDD, FICZ, and other high affinity AhR ligands dose-dependently determine the fate of CD4+ T cell differentiation. Toxicol Sci (2018) 161(2):310–20. doi: 10.1093/toxsci/kfx215 PMC5837604 29040756

[139] Kobayashi A Sogawa K Fujii-Kuriyama Y. Cooperative interaction between AhR.Arnt and Sp1 for the drug-inducible expression of CYP1A1 gene. J Biol Chem (1996) 271(21):12310–6. doi: 10.1074/jbc.271.21.12310 8647831

[140] Suh J Jeon YJ Kim HM Kang JS Kaminski NE Yang KH. Aryl hydrocarbon receptor-dependent inhibition of AP-1 activity by 2,3,7,8-tetrachlorodibenzo-p-dioxin in activated b cells. Toxicol Appl Pharmacol (2002) 181(2):116–23. doi: 10.1006/taap.2002.9403 12051995

[141] Kel A Reymann S Matys V Nettesheim P Wingender E Borlak J. A novel computational approach for the prediction of networked transcription factors of aryl hydrocarbon-receptor-regulated genes. Mol Pharmacol (2004) 66(6):1557–72. doi: 10.1124/mol.104.001677 15342792

[142] Khan S Barhoumi R Burghardt R Liu S Kim K Safe S. Molecular mechanism of inhibitory aryl hydrocarbon receptor-estrogen receptor/Sp1 cross talk in breast cancer cells. Mol Endocrinol (2006) 20(9):2199–214. doi: 10.1210/me.2006-0100 16675542

[143] Tian Y. Ah receptor and NF-kappaB interplay on the stage of epigenome. Biochem Pharmacol (2009) 77(4):670–80. doi: 10.1016/j.bcp.2008.10.023 19014911

[144] Fujii-Kuriyama Y Kawajiri K. Molecular mechanisms of the physiological functions of the aryl hydrocarbon (dioxin) receptor, a multifunctional regulator that senses and responds to environmental stimuli. Proc Jpn Acad Ser B Phys Biol Sci (2010) 86(1):40–53. doi: 10.2183/pjab.86.40 PMC3417568 20075607

[145] Vogel CF Khan EM Leung PS Gershwin ME Chang WL Wu D. Cross-talk between aryl hydrocarbon receptor and the inflammatory response: a role for nuclear factor-kappaB. J Biol Chem (2013) 289(3):1866–75. doi: 10.1074/jbc.m113.505578 PMC3894361 24302727

[146] Salisbury RL Sulentic CE. The AhR and NF-kappaB/Rel proteins mediate the inhibitory effect of 2,3,7,8-Tetrachlorodibenzo-p-Dioxin on the 3' immunoglobulin heavy chain regulatory region. Toxicol Sci (2015) 148(2):443–59. doi: 10.1093/toxsci/kfv193 PMC5009439 26377645

[147] Wright EJ De Castro KP Joshi AD Elferink CJ. Canonical and non-canonical aryl hydrocarbon receptor signaling pathways. Curr Opin Toxicol (2017) 2:87–92. doi: 10.1016/j.cotox.2017.01.001 32296737 PMC7158745

[148] Roy AL Sen R Roeder RG. Enhancer-promoter communication and transcriptional regulation of igh. Trends Immunol (2011) 32(11):532–9. doi: 10.1016/j.it.2011.06.012 PMC3200469 21855411

[149] Lieberson R Ong J Shi X Eckhardt LA. Immunoglobulin gene transcription ceases upon deletion of a distant enhancer. EMBO J (1995) 14(24):6229–38. doi: 10.1002/j.1460-2075.1995.tb00313.x PMC394747 8557042

[150] Pinaud E Khamlichi AA Le Morvan C Drouet M Nalesso V Le Bert M. Localization of the 3' IgH locus elements that effect long-distance regulation of class switch recombination. Immunity (2001) 15(2):187–99. doi: 10.1016/S1074-7613(01)00181-9 11520455

[151] Vincent-Fabert C Fiancette R Pinaud E Truffinet V Cogne N Cogne M. Genomic deletion of the whole IgH 3' regulatory region (hs3a, hs1,2, hs3b, and hs4) dramatically affects class switch recombination and ig secretion to all isotypes. Blood (2010) 116(11):1895–8. doi: 10.1182/blood-2010-01-264689 20538806

[152] Ju Z Volpi SA Hassan R Martinez N Giannini SL Gold T. Evidence for physical interaction between the immunoglobulin heavy chain variable region and the 3' regulatory region. J Biol Chem (2007) 282(48):35169–78. doi: 10.1074/jbc.M705719200 17921139

[153] Dunnick WA Collins JT Shi J Westfield G Fontaine C Hakimpour P. Switch recombination and somatic hypermutation are controlled by the heavy chain 3' enhancer region. J Exp Med (2009) 206(12):2613–23. doi: 10.1084/jem.20091280 PMC2806627 19887393

[154] Dunnick WA Shi J Zerbato JM Fontaine CA Collins JT. Enhancement of antibody class-switch recombination by the cumulative activity of four separate elements. J Immunol (2011) 187(9):4733–43. doi: 10.4049/jimmunol.1101808 PMC3197901 21949022

[155] Rouaud P Vincent-Fabert C Saintamand A Fiancette R Marquet M Robert I. The IgH 3' regulatory region controls somatic hypermutation in germinal center b cells. J Exp Med (2013) 210(8):1501–7. doi: 10.1084/jem.20130072 PMC3727322 23825188

[156] Dunnick WA Shi J Fontaine C Collins JT. Transgenes of the mouse immunoglobulin heavy chain locus, lacking distal elements in the 3' regulatory region, are impaired for class switch recombination. PloS One (2013) 8(2):e55842. doi: 10.1371/journal.pone.0055842 23409061 PMC3568100

[157] Saintamand A Rouaud P Saad F Rios G Cogne M Denizot Y. Elucidation of IgH 3' region regulatory role during class switch recombination *via* germline deletion. Nat Commun (2015) 6:7084. doi: 10.1038/ncomms8084 25959683

[158] Saintamand A Rouaud P Garot A Saad F Carrion C Oblet C. The IgH 3' regulatory region governs mu chain transcription in mature b lymphocytes and the b cell fate. Oncotarget (2015) 6(7):4845–52. doi: 10.18632/oncotarget.3010 PMC4467119 25742787

[159] Kim A Han L Santiago GE Verdun RE Yu K. Class-switch recombination in the absence of the IgH 3' regulatory region. J Immunol (2016) 197(7):2930–5. doi: 10.4049/jimmunol.1600530 PMC5026946 27559052

[160] Chi X Li Y Qiu X. V(D)J recombination, somatic hypermutation and class switch recombination of immunoglobulins: mechanism and regulation. Immunology (2020) 160(3):233–47. doi: 10.1111/imm.13176 PMC7341547 32031242

[161] Fleming A Castro-Dopico T Clatworthy MR. B cell class switching in intestinal immunity in health and disease. Scand J Immunol (2022) 95(2):e13139. doi: 10.1111/sji.13139 34978077 PMC9285483

[162] Hirano M Das S Guo P Cooper MD. The evolution of adaptive immunity in vertebrates. Adv Immunol (2011) 109:125–57. doi: 10.1016/B978-0-12-387664-5.00004-2 21569914

[163] Pancer Z Saha NR Kasamatsu J Suzuki T Amemiya CT Kasahara M. Variable lymphocyte receptors in hagfish. Proc Natl Acad Sci U.S.A. (2005) 102(26):9224–9. doi: 10.1073/pnas.0503792102 PMC1166628 15964979

[164] Cooper MD Alder MN. The evolution of adaptive immune systems. Cell (2006) 124(4):815–22. doi: 10.1016/j.cell.2006.02.001 16497590

[165] Senger K Hackney J Payandeh J Zarrin AA. Antibody isotype switching in vertebrates. Results Probl Cell Differ (2015) 57:295–324. doi: 10.1007/978-3-319-20819-0_13 26537387

[166] Flajnik MF. A cold-blooded view of adaptive immunity. Nat Rev Immunol (2018) 18(7):438–53. doi: 10.1038/s41577-018-0003-9 PMC6084782 29556016

[167] Kurosawa K Ohta K. Genetic diversification by somatic gene conversion. Genes (Basel) (2011) 2(1):48–58. doi: 10.3390/genes2010048 24710138 PMC3924843

[168] Woof JM Kerr MA. IgA function–variations on a theme. Immunology (2004) 113(2):175–7. doi: 10.1111/j.1365-2567.2004.01958.x PMC1782559 15379977

[169] Magadan-Mompo S Sanchez-Espinel C Gambon-Deza F. IgH loci of American alligator and saltwater crocodile shed light on IgA evolution. Immunogenetics (2013) 65(7):531–41. doi: 10.1007/s00251-013-0692-y 23558556

[170] Das S Hirano M Tako R McCallister C Nikolaidis N. Evolutionary genomics of immunoglobulin-encoding loci in vertebrates. Curr Genomics (2012) 13(2):95–102. doi: 10.2174/138920212799860652 23024601 PMC3308330

[171] Hahn ME Karchner SI Merson RR. Diversity as opportunity: Insights from 600 million years of AHR evolution. Curr Opin Toxicol (2017) 2:58–71. doi: 10.1016/j.cotox.2017.02.003 28286876 PMC5343764

[172] Hahn ME. Aryl hydrocarbon receptors: diversity and evolution. Chem Biol Interact (2002) 141(1-2):131–60. doi: 10.1016/S0009-2797(02)00070-4 12213389

[173] Quintana FJ Iglesias AH Farez MF Caccamo M Burns EJ Kassam N. Adaptive autoimmunity and Foxp3-based immunoregulation in zebrafish. PloS One (2010) 5(3):e9478. doi: 10.1371/journal.pone.0009478 20221429 PMC2832694

[174] Huang L Qi W Zuo Y Alias SA Xu W. The immune response of a warm water fish orange-spotted grouper (Epinephelus coioides) infected with a typical cold water bacterial pathogen aeromonas salmonicida is AhR dependent. Dev Comp Immunol (2020) 113:103779. doi: 10.1016/j.dci.2020.103779 32735958

[175] He R Zhao L Xu X Zheng W Zhang J Zhang J. Aryl hydrocarbon receptor is required for immune response in epinephelus coioides and danio rerio infected by pseudomonas plecoglossicida. Fish Shellfish Immunol (2020) 97:564–70. doi: 10.1016/j.fsi.2019.12.084 31891808

[176] Oreste U Ametrano A Coscia MR. On origin and evolution of the antibody molecule. Biol (Basel) (2021) 10(2):140–157. doi: 10.3390/biology10020140 PMC7916673 33578914

[177] Kaetzel CS Russell MW. Chapter 18 - phylogeny and comparative physiology of mucosal immunoglobulins. In: Mestecky J Strober W Russell MW Kelsall BL Cheroutre H Lambrecht BN, editors. Mucosal immunology, 4th ed. Boston: Academic Press (2015). p. 325–47.

[178] Wagner B Miller DC Lear TL Antczak DF. The complete map of the ig heavy chain constant gene region reveals evidence for seven IgG isotypes and for IgD in the horse. J Immunol (2004) 173(5):3230–42. doi: 10.4049/jimmunol.173.5.3230 15322185

[179] Shimizu A Takahashi N Yaoita Y Honjo T. Organization of the constant-region gene family of the mouse immunoglobulin heavy chain. Cell (1982) 28(3):499–506. doi: 10.1016/0092-8674(82)90204-5 6804095

[180] Flanagan JG Rabbitts TH. Arrangement of human immunoglobulin heavy chain constant region genes implies evolutionary duplication of a segment containing gamma, epsilon and alpha genes. Nature (1982) 300(5894):709–13. doi: 10.1038/300709a0 6817141

[181] Spieker-Polet H Yam PC Knight KL. Differential expression of 13 IgA-heavy chain genes in rabbit lymphoid tissues. J Immunol (1993) 150(12):5457–65. doi: 10.4049/jimmunol.150.12.5457 8515070

[182] Brusco A Saviozzi S Cinque F Bottaro A DeMarchi M. A recurrent breakpoint in the most common deletion of the ig heavy chain locus (del A1-GP-G2-G4-E). J Immunol (1999) 163(8):4392–8. doi: 10.4049/jimmunol.163.8.4392 10510380

[183] Brusco A Cariota U Bottaro A Boccazzi C Plebani A Ugazio AG. Variability of the immunoglobulin heavy chain constant region locus: a population study. Hum Genet (1995) 95(3):319–26. doi: 10.1007/BF00225201 7868126

[184] Hammarstrom L Carbonara AO DeMarchi M Lefranc G Lefranc MP Smith CI. Generation of the antibody repertoire in individuals with multiple immunoglobulin heavy chain constant region gene deletions. Scand J Immunol (1987) 25(2):189–94. doi: 10.1111/j.1365-3083.1987.tb01063.x 3823791

[185] Garzon-Ospina D Buitrago SP. Igh locus structure and evolution in platyrrhines: new insights from a genomic perspective. Immunogenetics (2020) 72(3):165–79. doi: 10.1007/s00251-019-01151-8 31838542

[186] D'Addabbo P Scascitelli M Giambra V Rocchi M Frezza D. Position and sequence conservation in amniota of polymorphic enhancer HS1.2 within the palindrome of IgH 3'Regulatory region. BMC Evol Biol (2011) 11:71. doi: 10.1186/1471-2148-11-71 21406099 PMC3068965

[187] Chen C Birshtein BK. Virtually identical enhancers containing a segment of homology to murine 3'IgH-E(hs1,2) lie downstream of human ig c alpha 1 and c alpha 2 genes. J Immunol (1997) 159(3):1310–8. doi: 10.4049/jimmunol.159.3.1310 9233627

[188] Pan Q Petit-Frere C Stavnezer J Hammarstrom L. Regulation of the promoter for human immunoglobulin gamma3 germ-line transcription and its interaction with the 3'alpha enhancer. Eur J Immunol (2000) 30(4):1019–29. doi: 10.1002/(SICI)1521-4141(200004)30:4<1019::AID-IMMU1019>3.0.CO;2-W 10760789

[189] Puget N Leduc C Oruc Z Moutahir M Le Bert M Khamlichi AA. Complete cis exclusion upon duplication of the emu enhancer at the immunoglobulin heavy chain locus. Mol Cell Biol (2015) 35(13):2231–41. doi: 10.1128/MCB.00294-15 PMC4456450 25896912

[190] Santos JM Braikia FZ Oudinet C Haddad D Conte C Dauba A. Duplication of a germline promoter downstream of the IgH 3' regulatory region impairs class switch recombination. Sci Rep (2018) 8(1):9164. doi: 10.1038/s41598-018-27448-4 29907762 PMC6003904

[191] Garzón-Ospina D Buitrago SP. Immunoglobulin heavy constant gamma gene evolution is modulated by both the divergent and birth-and-death evolutionary models. bioRxiv (2021). doi: 10.1101/2021.08.12.456010 36114442

[192] Magor BG Ross DA Pilstrom L Warr GW. Transcriptional enhancers and the evolution of the IgH locus. Immunol Today (1999) 20(1):13–7. doi: 10.1016/S0167-5699(98)01380-2 10081224

[193] Giambra V Fruscalzo A Giufre M Martinez-Labarga C Favaro M Rocchi M. Evolution of human IgH3'EC duplicated structures: both enhancers HS1,2 are polymorphic with variation of transcription factor's consensus sites. Gene (2005) 346:105–14. doi: 10.1016/j.gene.2004.10.009 15716094

[194] Duvvuri B Wu GE. Gene conversion-like events in the diversification of human rearranged IGHV3-23*01 gene sequences. Front Immunol (2012) 3:158. doi: 10.3389/fimmu.2012.00158 22715339 PMC3375636

[195] Chauveau C Cogne M. Palindromic structure of the IgH 3'locus control region. Nat Genet (1996) 14(1):15–6. doi: 10.1038/ng0996-15 8782813

[196] Garrett FE Emelyanov AV Sepulveda MA Flanagan P Volpi S Li F. Chromatin architecture near a potential 3' end of the igh locus involves modular regulation of histone modifications during b-cell development and *in vivo* occupancy at CTCF sites. Mol Cell Biol (2005) 25(4):1511–25. doi: 10.1128/MCB.25.4.1511-1525.2005 PMC548023 15684400

[197] Sette M D'Addabbo P Kelly G Cicconi A Micheli E Cacchione S. Evidence for a quadruplex structure in the polymorphic hs1.2 enhancer of the immunoglobulin heavy chain 3' regulatory regions and its conservation in mammals. Biopolymers (2016) 105(11):768–78. doi: 10.1002/bip.22891 PMC5516150 27287611

[198] Ju Z Chatterjee S Birshtein BK. Interaction between the immunoglobulin heavy chain 3' regulatory region and the IgH transcription unit during b cell differentiation. Mol Immunol (2011) 49(1-2):297–303. doi: 10.1016/j.molimm.2011.08.024 21945019 PMC3238056

[199] Birshtein BK. The role of CTCF binding sites in the 3' immunoglobulin heavy chain regulatory region. Front Genet (2012) 3:251. doi: 10.3389/fgene.2012.00251 23162572 PMC3499808

[200] Saintamand A Vincent-Fabert C Garot A Rouaud P Oruc Z Magnone V. Deciphering the importance of the palindromic architecture of the immunoglobulin heavy-chain 3' regulatory region. Nat Commun (2016) 7:10730. doi: 10.1038/ncomms10730 26883548 PMC4757795

[201] Spiegel J Cuesta SM Adhikari S Hansel-Hertsch R Tannahill D Balasubramanian S. G-Quadruplexes are transcription factor binding hubs in human chromatin. Genome Biol (2021) 22(1):117. doi: 10.1186/s13059-021-02324-z 33892767 PMC8063395

[202] Sepulveda MA Emelyanov AV Birshtein BK. NF-kappa b and Oct-2 synergize to activate the human 3' igh hs4 enhancer in b cells. J Immunol (2004) 172(2):1054–64. doi: 10.4049/jimmunol.172.2.1054 14707079

[203] Grant PA Thompson CB Pettersson S. IgM receptor-mediated transactivation of the IgH 3' enhancer couples a novel elf-1-AP-1 protein complex to the developmental control of enhancer function. EMBO J (1995) 14(18):4501–13. doi: 10.1002/j.1460-2075.1995.tb00129.x PMC394542 7556093

[204] Michaelson JS Singh M Snapper CM Sha WC Baltimore D Birshtein BK. Regulation of 3' IgH enhancers by a common set of factors, including kappa b-binding proteins. J Immunol (1996) 156(8):2828–39. doi: 10.4049/jimmunol.156.8.2828 8609402

[205] Linderson Y Cross D Neurath MF Pettersson S. NFE, a new transcriptional activator that facilitates p50 and c-rel-dependent IgH 3' enhancer activity. Eur J Immunol (1997) 27(2):468–75. doi: 10.1002/eji.1830270218 9045919

[206] Podojil JR Kin NW Sanders VM. CD86 and beta2-adrenergic receptor signaling pathways, respectively, increase Oct-2 and OCA-b expression and binding to the 3'-IgH enhancer in b cells. J Biol Chem (2004) 279(22):23394–404. doi: 10.1074/jbc.M313096200 15024018

[207] Linderson Y Eberhard D Malin S Johansson A Busslinger M Pettersson S. Corecruitment of the Grg4 repressor by PU.1 is critical for Pax5-mediated repression of b-cell-specific genes. EMBO Rep (2004) 5(3):291–6. doi: 10.1038/sj.embor.7400089 PMC1299001 14993928

[208] Kim EC Edmonston CR Wu X Schaffer A Casali P. The HoxC4 homeodomain protein mediates activation of the immunoglobulin heavy chain 3' hs1,2 enhancer in human b cells. relevance to class switch DNA recombination. J Biol Chem (2004) 279(40):42258–69. doi: 10.1074/jbc.M407496200 PMC4631311 15252056

[209] Frezza D Giambra V Mattioli C Piccoli K Massoud R Siracusano A. Allelic frequencies of 3' ig heavy chain locus enhancer HS1,2-a associated with ig levels in patients with schizophrenia. Int J Immunopathol Pharmacol (2009) 22(1):115–23. doi: 10.1177/039463200902200113 PMC2721332 19309558

[210] Chatterjee S Ju Z Hassan R Volpi SA Emelyanov AV Birshtein BK. Dynamic changes in binding of immunoglobulin heavy chain 3' regulatory region to protein factors during class switching. J Biol Chem (2011) 286(33):29303–12. doi: 10.1074/jbc.M111.243543 PMC3190736 21685395

[211] Birshtein BK. Epigenetic regulation of individual modules of the immunoglobulin heavy chain locus 3' regulatory region. Front Immunol (2014) 5:163. doi: 10.3389/fimmu.2014.00163 24795714 PMC4000994

[212] Jones BG Penkert RR Xu B Fan Y Neale G Gearhart PJ. Binding of estrogen receptors to switch sites and regulatory elements in the immunoglobulin heavy chain locus of activated b cells suggests a direct influence of estrogen on antibody expression. Mol Immunol (2016) 77:97–102. doi: 10.1016/j.molimm.2016.07.015 27494228 PMC5010968

[213] Snyder AD Ochs SD Johnson BE Sulentic CEW. Aryl hydrocarbon receptor-induced activation of the human IGH hs1.2 enhancer: Mutational analysis of putative regulatory binding motifs. Mol Immunol (2020) 120:164–78. doi: 10.1016/j.molimm.2020.02.002 PMC7103136 32146146

[214] Frezza D Tolusso B Giambra V Gremese E Marchini M Nowik M. Polymorphisms of the IgH enhancer HS1.2 and risk of systemic lupus erythematosus. Ann Rheum Dis (2012) 71(8):1309–15. doi: 10.1136/ard.2010.147025 22294636

[215] Raiber EA Kranaster R Lam E Nikan M Balasubramanian S. A non-canonical DNA structure is a binding motif for the transcription factor SP1 *in vitro* . Nucleic Acids Res (2012) 40(4):1499–508. doi: 10.1093/nar/gkr882 PMC3287196 22021377

[216] Lago S Nadai M Cernilogar FM Kazerani M Dominiguez Moreno H Schotta G. Promoter G-quadruplexes and transcription factors cooperate to shape the cell type-specific transcriptome. Nat Commun (2021) 12(1):3885. doi: 10.1038/s41467-021-24198-2 34162892 PMC8222265

[217] Natoli G. Control of NF-kappaB-dependent transcriptional responses by chromatin organization. Cold Spring Harb Perspect Biol (2009) 1(4):a000224. doi: 10.1101/cshperspect.a000224 20066094 PMC2773620

[218] Baral A Kumar P Halder R Mani P Yadav VK Singh A. Quadruplex-single nucleotide polymorphisms (Quad-SNP) influence gene expression difference among individuals. Nucleic Acids Res (2012) 40(9):3800–11. doi: 10.1093/nar/gkr1258 PMC3351168 22238381

[219] Shen J Varshney D Simeone A Zhang X Adhikari S Tannahill D. Promoter G-quadruplex folding precedes transcription and is controlled by chromatin. Genome Biol (2021) 22(1):143. doi: 10.1186/s13059-021-02346-7 33962653 PMC8103603

[220] Pinaud E Aupetit C Chauveau C Cogne M. Identification of a homolog of the c alpha 3'/hs3 enhancer and of an allelic variant of the 3'IgH/hs1,2 enhancer downstream of the human immunoglobulin alpha 1 gene. Eur J Immunol (1997) 27(11):2981–5. doi: 10.1002/eji.1830271134 9394827

[221] Giambra V Martinez-Labarga C Giufre M Modiano D Simpore J Gisladottir BK. Immunoglobulin enhancer HS1,2 polymorphism: a new powerful anthropogenetic marker. Ann Hum Genet (2006) 70(Pt 6):946–50. doi: 10.1111/j.1469-1809.2006.00273.x 17044868

[222] Mills FC Harindranath N Mitchell M Max EE. Enhancer complexes located downstream of both human immunoglobulin calpha genes. J Exp Med (1997) 186(6):845–58. doi: 10.1084/jem.186.6.845 PMC2199054 9294139

[223] Denizot Y Pinaud E Aupetit C Le Morvan C Magnoux E Aldigier JC. Polymorphism of the human alpha1 immunoglobulin gene 3' enhancer hs1,2 and its relation to gene expression. Immunology (2001) 103(1):35–40. doi: 10.1046/j.1365-2567.2001.01217.x 11380690 PMC1783220

[224] Guglielmi L Truffinet V Magnoux E Cogne M Denizot Y. The polymorphism of the locus control region lying downstream the human IgH locus is restricted to hs1,2 but not to hs3 and hs4 enhancers. Immunol Lett (2004) 94(1-2):77–81. doi: 10.1016/j.imlet.2004.04.003 15234538

[225] Aupetit C Drouet M Pinaud E Denizot Y Aldigier JC Bridoux F. Alleles of the alpha1 immunoglobulin gene 3' enhancer control evolution of IgA nephropathy toward renal failure. Kidney Int (2000) 58(3):966–71. doi: 10.1046/j.1523-1755.2000.00253.x 10972660

[226] Drouet M Aupetit C Denizot Y Bois M Bridoux F Aldigier JC. Analysis of three genetic markers in IgA nephropathy patients from a single region. Clin Nephrol (2002) 57(4):253–60. doi: 10.5414/CNP57253 12005241

[227] Frezza D Giambra V Cianci R Fruscalzo A Giufre M Cammarota G. Increased frequency of the immunoglobulin enhancer HS1,2 allele 2 in coeliac disease. Scand J Gastroenterol (2004) 39(11):1083–7. doi: 10.1080/00365520410007999 15545166

[228] Frezza D Giambra V Tolusso B De Santis M Bosello S Vettori S. Polymorphism of immunoglobulin enhancer element HS1,2A: allele *2 associates with systemic sclerosis. comparison with HLA-DR and DQ allele frequency. Ann Rheum Dis (2007) 66(9):1210–5. doi: 10.1136/ard.2006.066597 PMC1955163 17392350

[229] Cianci R Giambra V Mattioli C Esposito M Cammarota G Scibilia G. Increased frequency of ig heavy-chain HS1,2-a enhancer *2 allele in dermatitis herpetiformis, plaque psoriasis, and psoriatic arthritis. J Invest Dermatol (2008) 128(8):1920–4. doi: 10.1038/jid.2008.40 18323783

[230] Tolusso B Frezza D Mattioli C Fedele AL Bosello S Faustini F. Allele *2 of the HS1,2A enhancer of the ig regulatory region associates with rheumatoid arthritis. Ann Rheum Dis (2009) 68(3):416–9. doi: 10.1136/ard.2008.095414 PMC2633630 18952640

[231] Giambra V Cianci R Lolli S Mattioli C Tampella G Cattalini M. Allele *1 of HS1.2 enhancer associates with selective IgA deficiency and IgM concentration. J Immunol (2009) 183(12):8280–5. doi: 10.4049/jimmunol.0902426 20007591

[232] Vincent-Fabert C Fiancette R Cogne M Pinaud E Denizot Y. The IgH 3' regulatory region and its implication in lymphomagenesis. Eur J Immunol (2010) 40(12):3306–11. doi: 10.1002/eji.201040778 21080376

[233] Canestri S Totaro MC Serone E Tolusso B Frezza D Gremese E. Association between the response to b cell depletion therapy and the allele*2 of the HS1,2A enhancer in seropositive rheumatoid arthritis patients. Reumatismo (2012) 64(6):368–73. doi: 10.4081/reumatismo.2012.368 23285480

[234] Serone E Daleno C Principi N Porretti L Iacoacci V Gargioli C. The change in ig regulation from children to adults disconnects the correlation with the 3'RR hs1.2 polymorphism. BMC Immunol (2014) 15:45. doi: 10.1186/s12865-014-0045-0 25391515 PMC4234878

[235] D'Addabbo P Serone E Esposito M Vaccari G Gargioli C Frezza D. Association between psoriasis and haplotypes of the IgH 3' regulatory region 1. Gene (2018) 669:47–51. doi: 10.1016/j.gene.2018.05.090 29802990

[236] Alexis AF Blackcloud P. Psoriasis in skin of color: epidemiology, genetics, clinical presentation, and treatment nuances. J Clin Aesthet Dermatol (2014) 7(11):16–24. PMC4255694 25489378

[237] Colucci M Frezza D Gambassi G De Vito F Iaquinta A Massaro MG. Functional associations between polymorphic regions of the human 3'IgH locus and COVID-19 disease. Gene (2022) 838:146698. doi: 10.1016/j.gene.2022.146698 35772651 PMC9241982

[238] Frezza D Martinez-Labarga C Giambra V Serone E Scano G Rickards O. Concerted variation of the 3' regulatory region of ig heavy chain and gm haplotypes across human continental populations. Am J Phys Anthropol (2020) 171(4):671–82. doi: 10.1002/ajpa.24011 31957883

[239] Jefferis R Lefranc MP. Human immunoglobulin allotypes: possible implications for immunogenicity. MAbs (2009) 1(4):332–8. doi: 10.4161/mabs.1.4.9122 PMC2726606 20073133

[240] Lopez S van Dorp L Hellenthal G. Human dispersal out of Africa: A lasting debate. Evol Bioinform Online (2015) 11(Suppl 2):57–68. doi: 10.4137/EBO.S33489 PMC4844272 27127403

[241] Shi X Eckhardt LA. Deletional analyses reveal an essential role for the hs3b/hs4 IgH 3' enhancer pair in an ig-secreting but not an earlier-stage b cell line. Int Immunol (2001) 13(8):1003–12. doi: 10.1093/intimm/13.8.1003 11470770

[242] Volpi SA Verma-Gaur J Hassan R Ju Z Roa S Chatterjee S. Germline deletion of igh 3' regulatory region elements hs 5, 6, 7 (hs5-7) affects b cell-specific regulation, rearrangement, and insulation of the igh locus. J Immunol (2012) 188(6):2556–66. doi: 10.4049/jimmunol.1102763 PMC3430471 22345664

[243] Le Noir S Boyer F Lecardeur S Brousse M Oruc Z Cook-Moreau J. Functional anatomy of the immunoglobulin heavy chain 3 super-enhancer needs not only core enhancer elements but also their unique DNA context. Nucleic Acids Res (2017) 45(10):5829–37. doi: 10.1093/nar/gkx203 PMC5449612 28369649

[244] Delgado-Benito V Rosen DB Wang Q Gazumyan A Pai JA Oliveira TY. The chromatin reader ZMYND8 regulates igh enhancers to promote immunoglobulin class switch recombination. Mol Cell (2018) 72 (4):636–649. doi: 10.1016/j.molcel.2018.08.042 PMC6242708 30293785

[245] Chauveau C Pinaud E Cogne M. Synergies between regulatory elements of the immunoglobulin heavy chain locus and its palindromic 3' locus control region. Eur J Immunol (1998) 28(10):3048–56. doi: 10.1002/(SICI)1521-4141(199810)28:10<3048::AID-IMMU3048>3.0.CO;2-V 9808173

[246] Stevens S Ong J Kim U Eckhardt LA Roeder RG. Role of OCA-b in 3'-IgH enhancer function. J Immunol (2000) 164(10):5306–12. doi: 10.4049/jimmunol.164.10.5306 10799892

[247] Laurencikiene J Deveikaite V Severinson E. HS1,2 enhancer regulation of germline epsilon and gamma2b promoters in murine b lymphocytes: evidence for specific promoter-enhancer interactions. J Immunol (2001) 167(6):3257–65. doi: 10.4049/jimmunol.167.6.3257 11544313

[248] Pan Q Hammarstrom L. Molecular basis of IgG subclass deficiency. Immunol Rev (2000) 178:99–110. doi: 10.1034/j.1600-065X.2000.17815.x 11213812

[249] Stavnezer J Schrader CE. IgH chain class switch recombination: mechanism and regulation. J Immunol (2014) 193(11):5370–8. doi: 10.4049/jimmunol.1401849 PMC4447316 25411432

[250] Santos JM Braikia FZ Oudinet C Dauba A Khamlichi AA. Two modes of cis-activation of switch transcription by the IgH superenhancer. Proc Natl Acad Sci U.S.A. (2019) 116(29):14708–13. doi: 10.1073/pnas.1902250116 PMC6642387 31266889

[251] Yu K Chedin F Hsieh CL Wilson TE Lieber MR. R-loops at immunoglobulin class switch regions in the chromosomes of stimulated b cells. Nat Immunol (2003) 4(5):442–51. doi: 10.1038/ni919 12679812

[252] Wuerffel R Wang L Grigera F Manis J Selsing E Perlot T. S-s synapsis during class switch recombination is promoted by distantly located transcriptional elements and activation-induced deaminase. Immunity (2007) 27(5):711–22. doi: 10.1016/j.immuni.2007.09.007 PMC4979535 17980632

[253] Chandra V Bortnick A Murre C. AID targeting: old mysteries and new challenges. Trends Immunol (2015) 36(9):527–35. doi: 10.1016/j.it.2015.07.003 PMC4567449 26254147

[254] Vincent-Fabert C Truffinet V Fiancette R Cogne N Cogne M Denizot Y. Ig synthesis and class switching do not require the presence of the hs4 enhancer in the 3' IgH regulatory region. J Immunol (2009) 182(11):6926–32. doi: 10.4049/jimmunol.0900214 19454689

[255] Bruzeau C Moreau J Le Noir S Pinaud E. Panorama of stepwise involvement of the IgH 3' regulatory region in murine b cells. Adv Immunol (2021) 149:95–114. doi: 10.1016/bs.ai.2021.03.004 33993921

[256] Romer EJ Sulentic CEW. Hydrogen peroxide modulates immunoglobulin expression by targeting the 3'Igh regulatory region through an NFkappaB-dependent mechanism. Free Radic Res (2011) 45(7):796–809. doi: 10.3109/10715762.2011.581280 21599461 PMC4545260

[257] Sharma M Salisbury RL Maurer EI Hussain SM Sulentic CE. Gold nanoparticles induce transcriptional activity of NF-kappaB in a b-lymphocyte cell line. Nanoscale (2013) 5(9):3747–56. doi: 10.1039/c3nr30071d PMC10156170 23503581

[258] Neurath MF Strober W Wakatsuki Y. The murine ig 3' alpha enhancer is a target site with repressor function for the b cell lineage-specific transcription factor BSAP (NF-HB, s alpha-BP). J Immunol (1994) 153(2):730–42. doi: 10.4049/jimmunol.153.2.730 8021508

[259] Neurath MF Max EE Strober W. Pax5 (BSAP) regulates the murine immunoglobulin 3' alpha enhancer by suppressing binding of NF-alpha p, a protein that controls heavy chain transcription. Proc Natl Acad Sci (1995) 92(12):5336–40. doi: 10.1073/pnas.92.12.5336 PMC41689 7777508

[260] Cobaleda C Schebesta A Delogu A Busslinger M. Pax5: the guardian of b cell identity and function. Nat Immunol (2007) 8(5):463–70. doi: 10.1038/ni1454 17440452

[261] Barberis A Widenhorn K Vitelli L Busslinger M. A novel b-cell lineage-specific transcription factor present at early but not late stages of differentiation. Genes Dev (1990) 4(5):849–59. doi: 10.1101/gad.4.5.849 2116362

[262] Holmes ML Pridans C Nutt SL. The regulation of the b-cell gene expression programme by Pax5. Immunol Cell Biol (2008) 86(1):47–53. doi: 10.1038/sj.icb.7100134 17998914

[263] Jones BG Penkert RR Surman SL Sealy RE Pelletier S Xu B. Matters of life and death: How estrogen and estrogen receptor binding to the immunoglobulin heavy chain locus may influence outcomes of infection, allergy, and autoimmune disease. Cell Immunol (2019) 346:103996. doi: 10.1016/j.cellimm.2019.103996 31703914 PMC7368653

[264] Sealy RE Jones BG Surman SL Penkert RR Pelletier S Neale G. Will attention by vaccine developers to the host's nuclear hormone levels and immunocompetence improve vaccine success? Vaccines (Basel) (2019) 7(1):26. doi: 10.3390/vaccines7010026 30818795 PMC6466149

[265] Jones BG Sealy RE Penkert RR Surman SL Maul RW Neale G. Complex sex-biased antibody responses: estrogen receptors bind estrogen response elements centered within immunoglobulin heavy chain gene enhancers. Int Immunol (2019) 31(3):141–56. doi: 10.1093/intimm/dxy074 PMC6400052 30407507

[266] Hu Y Pan Q Pardali E Mills FC Bernstein RM Max EE. Regulation of germline promoters by the two human ig heavy chain 3' alpha enhancers. J Immunol (2000) 164(12):6380–6. doi: 10.4049/jimmunol.164.12.6380 10843693

[267] Rabbani H Pan Q Kondo N Smith CI Hammarstrom L. Duplications and deletions of the human IGHC locus: evolutionary implications. Immunogenetics (1996) 45(2):136–41. doi: 10.1007/s002510050181 8952963

[268] NTP. NTP research report on the consortium linking academic and regulatory insights on bisphenol a toxicity (CLARITY-BPA): A compendium of published findings: Research report 18. Research Triangle Park (NC) (2021). doi: 10.22427/NTP-RR-18 34910417

[269] Vandenberg LN Hunt PA Gore AC. Endocrine disruptors and the future of toxicology testing - lessons from CLARITY-BPA. Nat Rev Endocrinol (2019) 15(6):366–74. doi: 10.1038/s41574-019-0173-y 30842650

[270] Rannug A. 6-Formylindolo[3,2-b]carbazole, a potent ligand for the aryl hydrocarbon receptor produced both endogenously and by microorganisms, can either promote or restrain inflammatory responses. Front Toxicol (2022) 4:775010. doi: 10.3389/ftox.2022.775010 35295226 PMC8915874

[271] Tischkau SA. Mechanisms of circadian clock interactions with aryl hydrocarbon receptor signalling. Eur J Neurosci (2020) 51(1):379–95. doi: 10.1111/ejn.14361 PMC9530885 30706546

[272] Bissonnette R Stein Gold L Rubenstein DS Tallman AM Armstrong A. Tapinarof in the treatment of psoriasis: A review of the unique mechanism of action of a novel therapeutic aryl hydrocarbon receptor-modulating agent. J Am Acad Dermatol (2021) 84(4):1059–67. doi: 10.1016/j.jaad.2020.10.085 33157177

