# Erratum: Evolutive emergence and divergence of an Ig regulatory node: an environmental sensor getting cues from the aryl hydrocarbon receptor?

**DOI:** 10.3389/fimmu.2023.1356336

**Published:** 2024-01-05

**Authors:** Frontiers Production Office

**Affiliations:** Frontiers Media SA, Lausanne, Switzerland

**Keywords:** B lymphocytes, AhR (aryl hydrocarbon receptor), dioxin, antibody expression, evolution and regulation of the immunoglobulin heavy chain (IgH), IgH 3′ regulatory region (3′RR), species differences, human polymorphisms

Due to a production error, asterisks were used instead of bullet points under the Human column in Table 1.

The publisher apologizes for this mistake.

The original version of this article has been updated.

