# Supplementary figures and images for: Evolutive emergence and divergence of an Ig regulatory node: An environmental sensor getting cues from the aryl hydrocarbon receptor?
Source: Front Immunol. 2023 Feb 3;14:996119. doi: 10.3389/fimmu.2023.996119 (PMC9936319; doi:10.3389/fimmu.2023.996119)

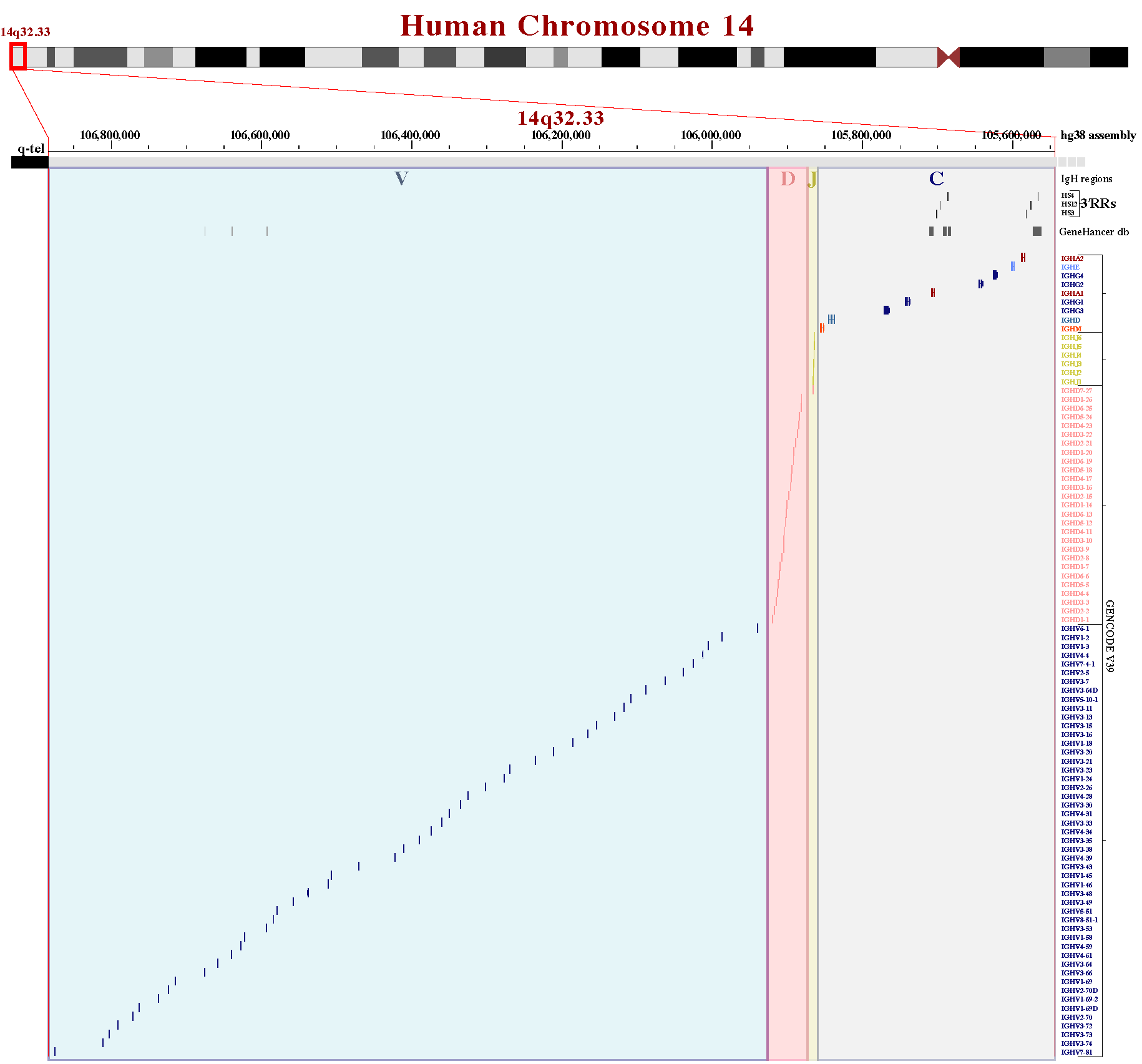

Supplement: Supplementary Figure 1 — Chromosomal location of the segmental features of the human IgH gene. The top image represents the ideogram of human chromosome 14. Dark boxes depict the approximate location of bands, as seen on the Giemsa-stained chromosome. The constriction site (brown triangles) indicates the centromeric region, thus the p arm is on the right and the longer q arm on the left of the ideogram. The red box highlights the 14q32.33 band harboring the IgH region that is annotated in detail in the lower part of the figure. The gene annotation of the IgH region comes from the V39 release of GENCODE on the hg38 assembly of the human genome. The faded background colors highlight the different size of the genomic areas hosting V, D, J or C regions. Enhancers of the whole IgH region are also positioned, as reported in GeneHancer database, but we integrated these data with the position of the well-known enhancers belonging to the human 3’RRs. [file Image_1.tiff]

human hs1.2 enhancer allele \*4

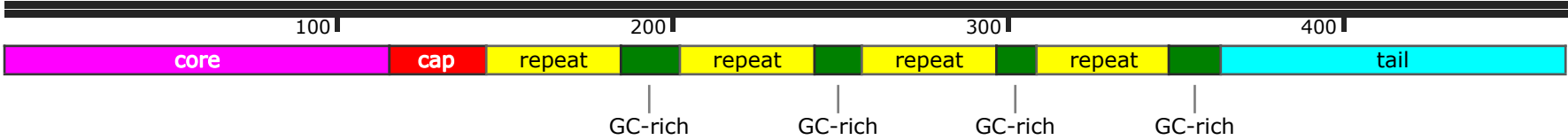

**AY530201.1**  
465 bp

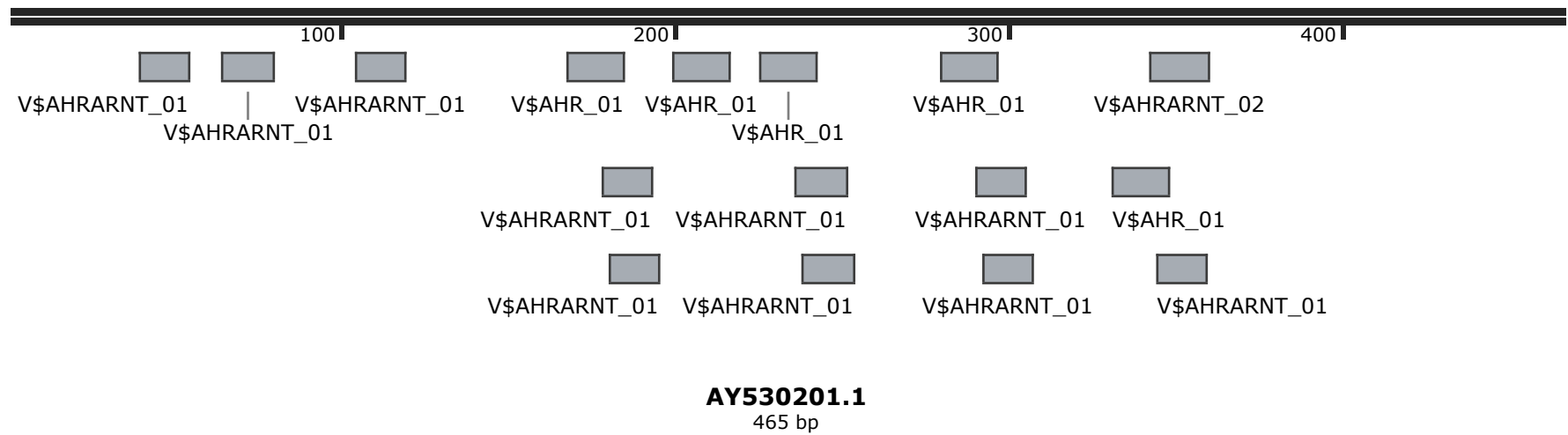

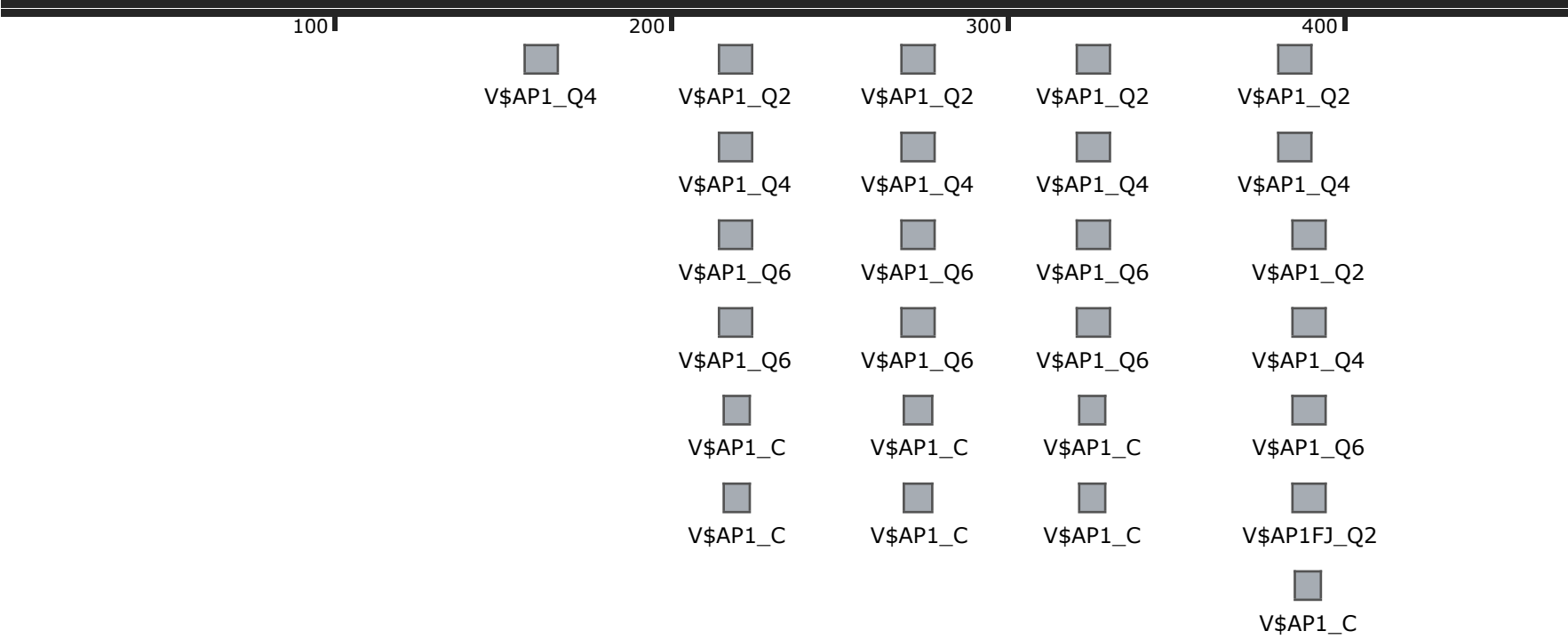

**AY530201.1**  
465 bp

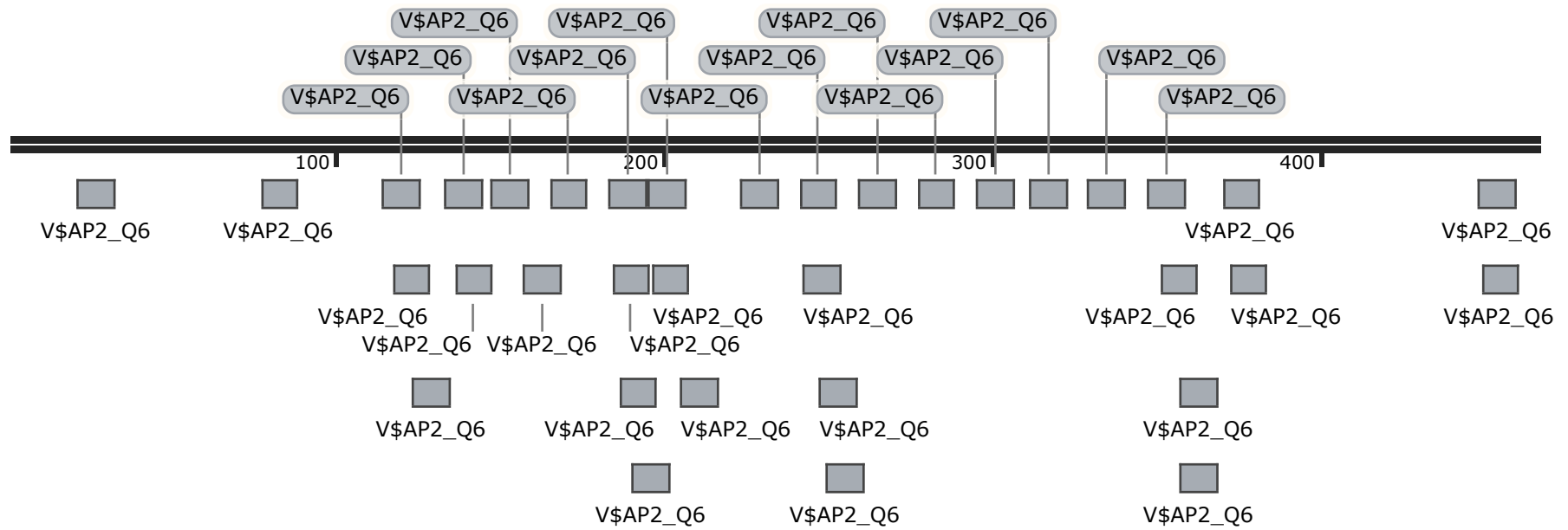

**AY530201.1**  
465 bp

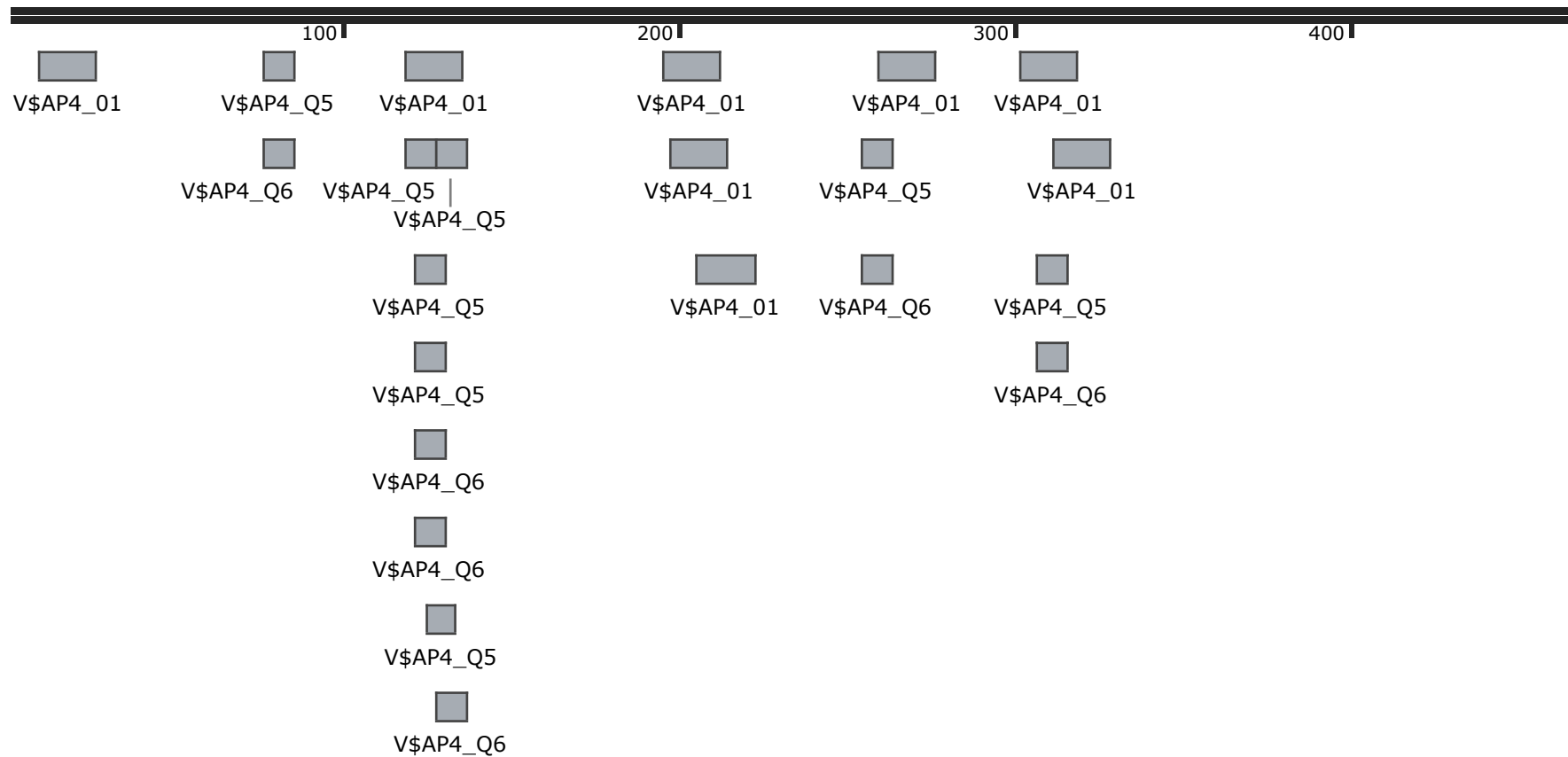

**AY530201.1**  
465 bp

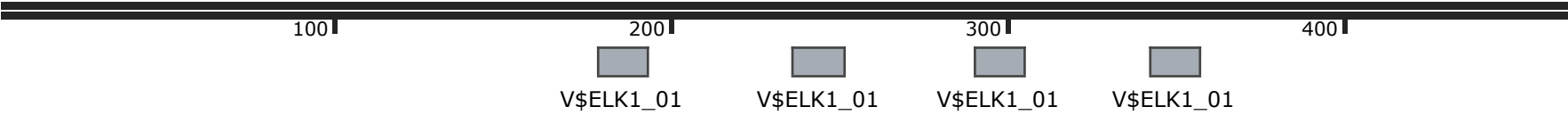

**AY530201.1**  
465 bp

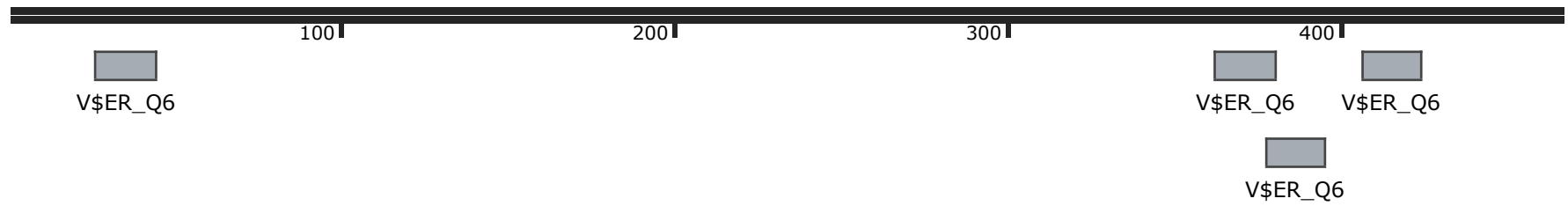

**AY530201.1**  
465 bp

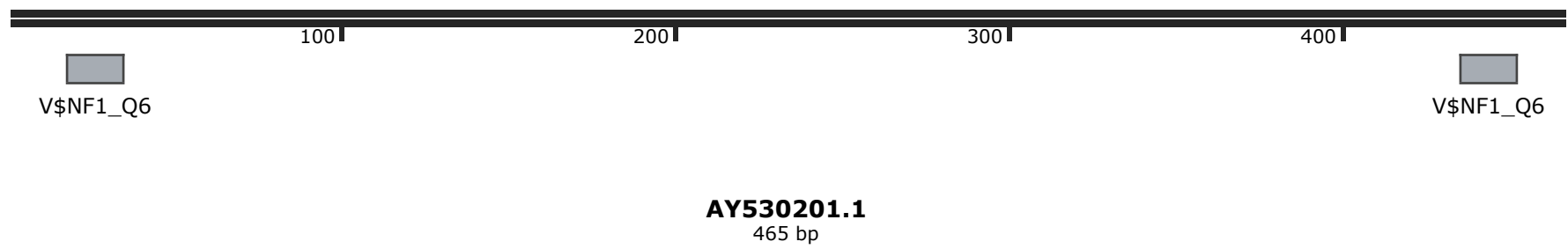

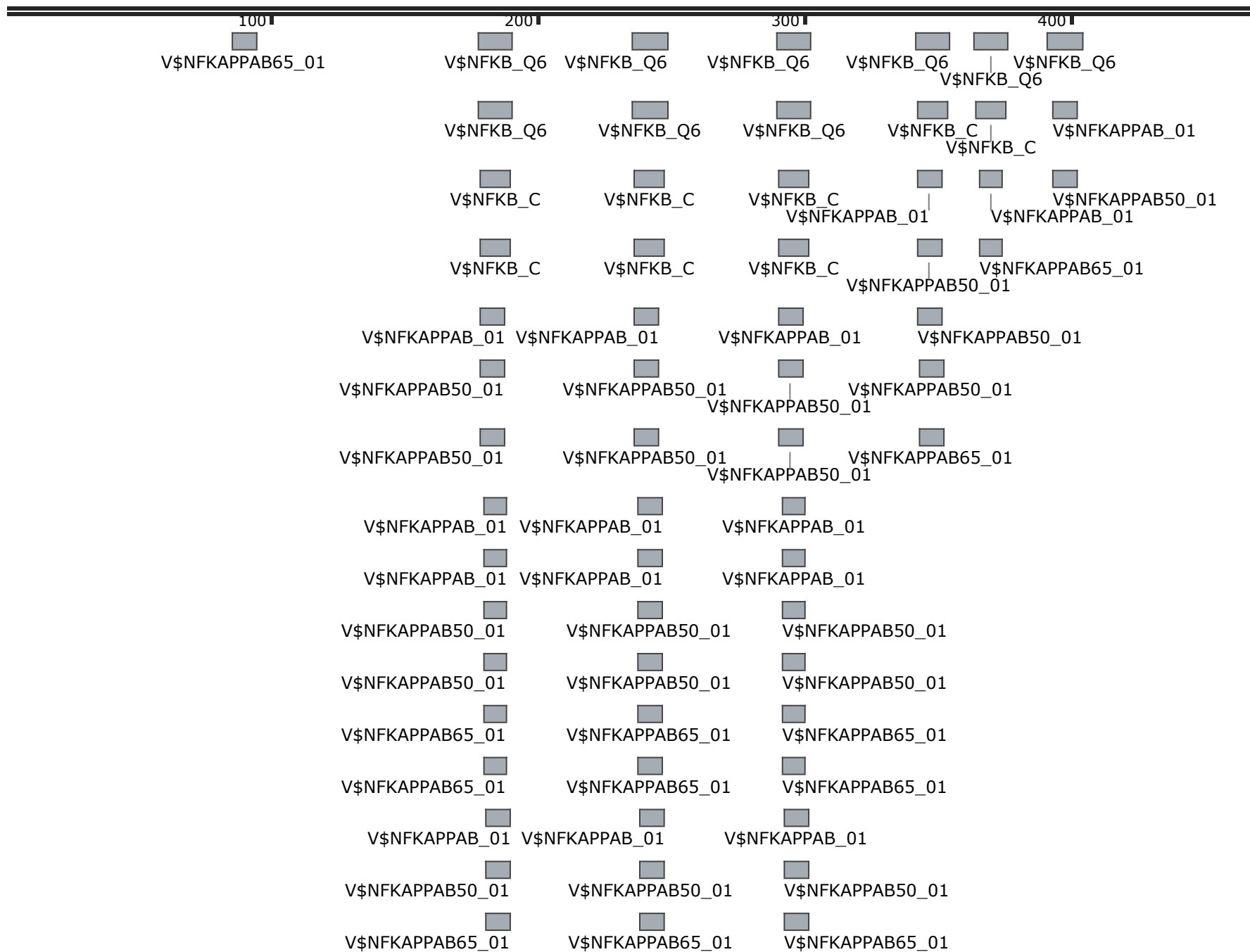

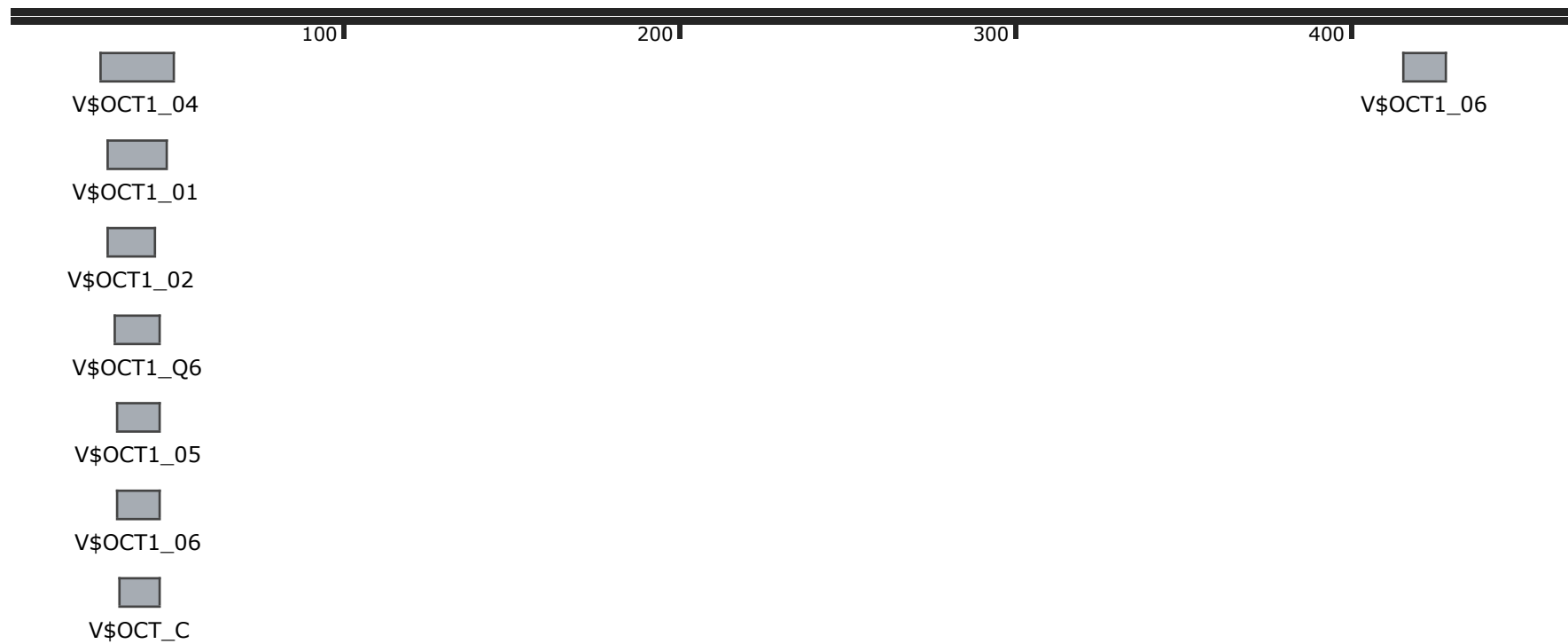

**AY530201.1**  
465 bp

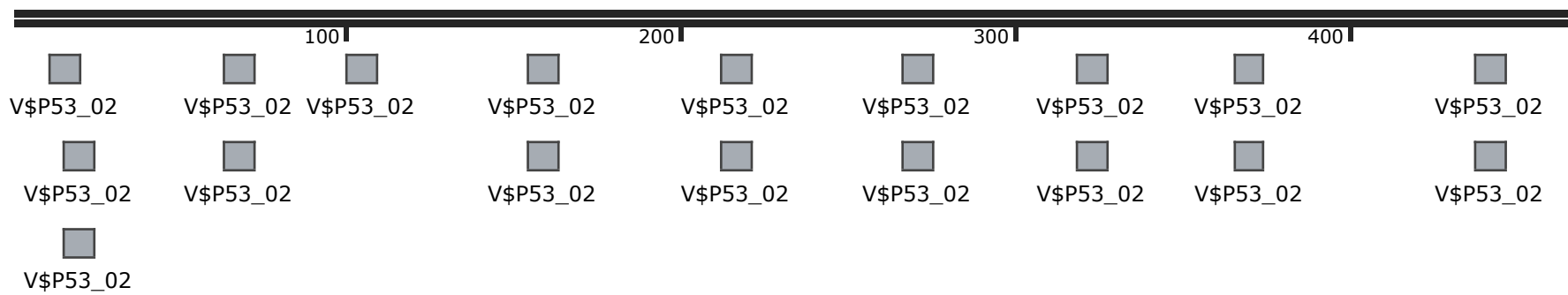

**AY530201.1**  
465 bp

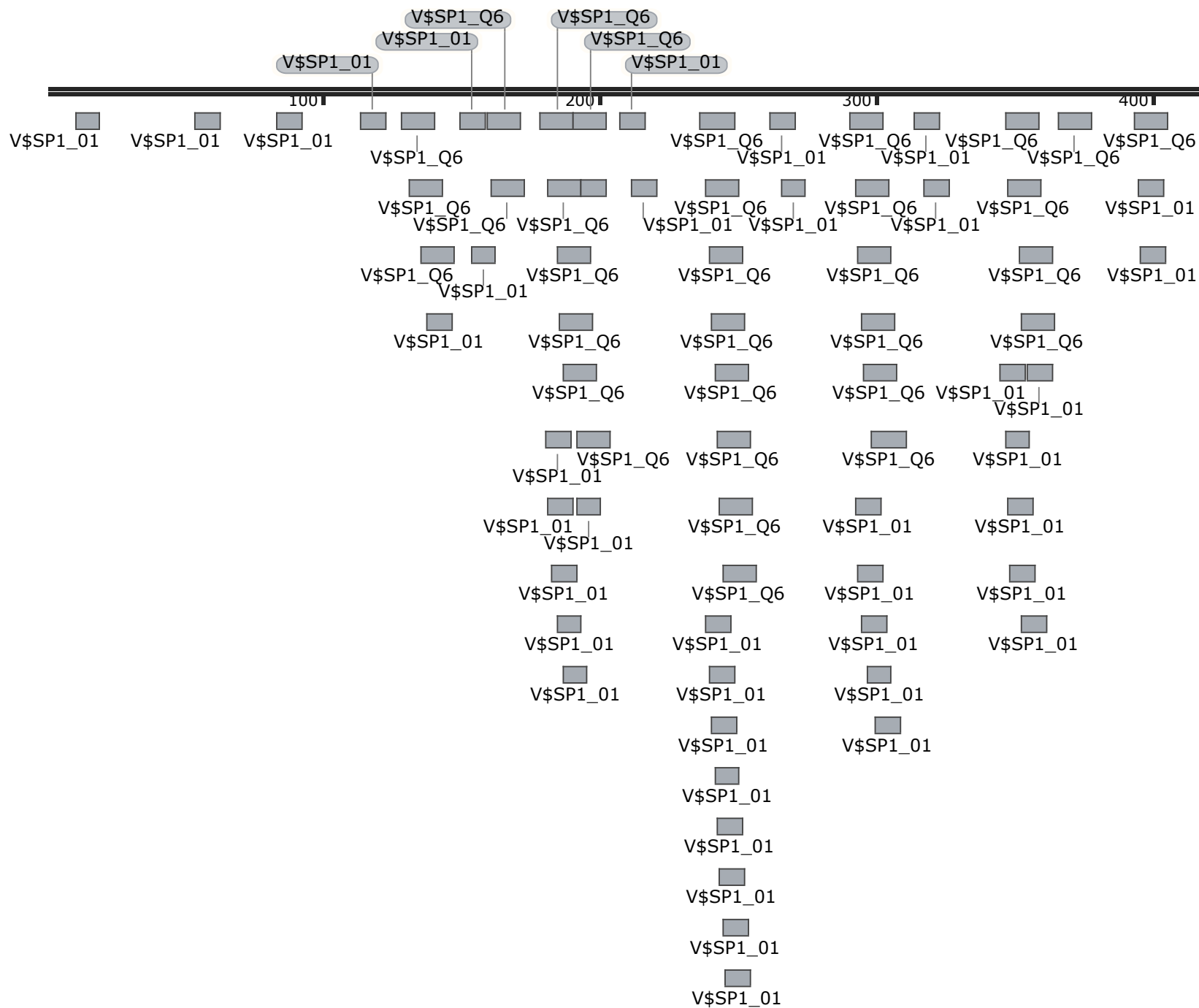

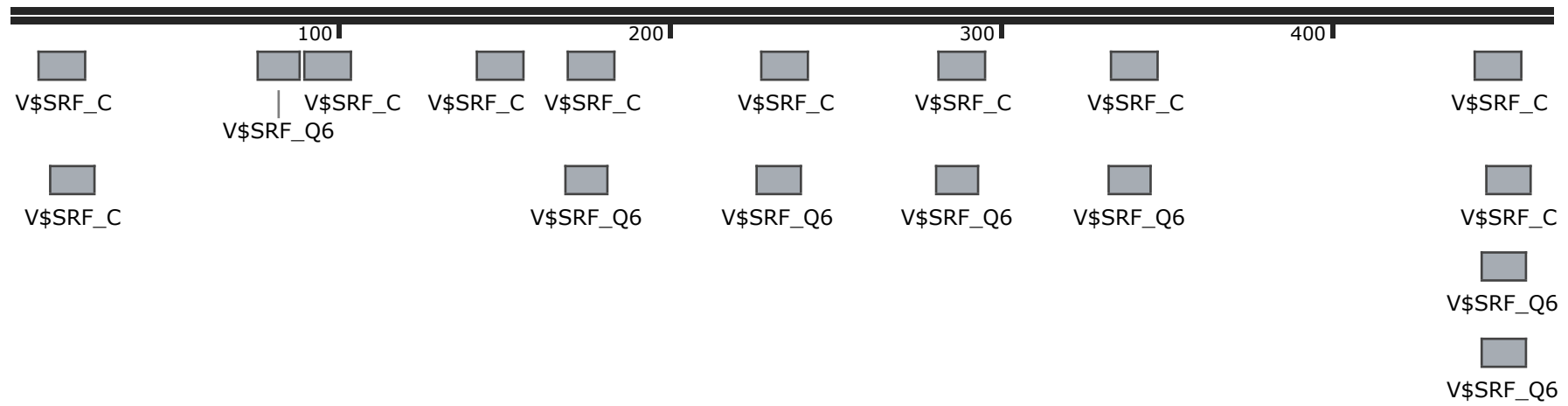

**AY530201.1**  
465 bp

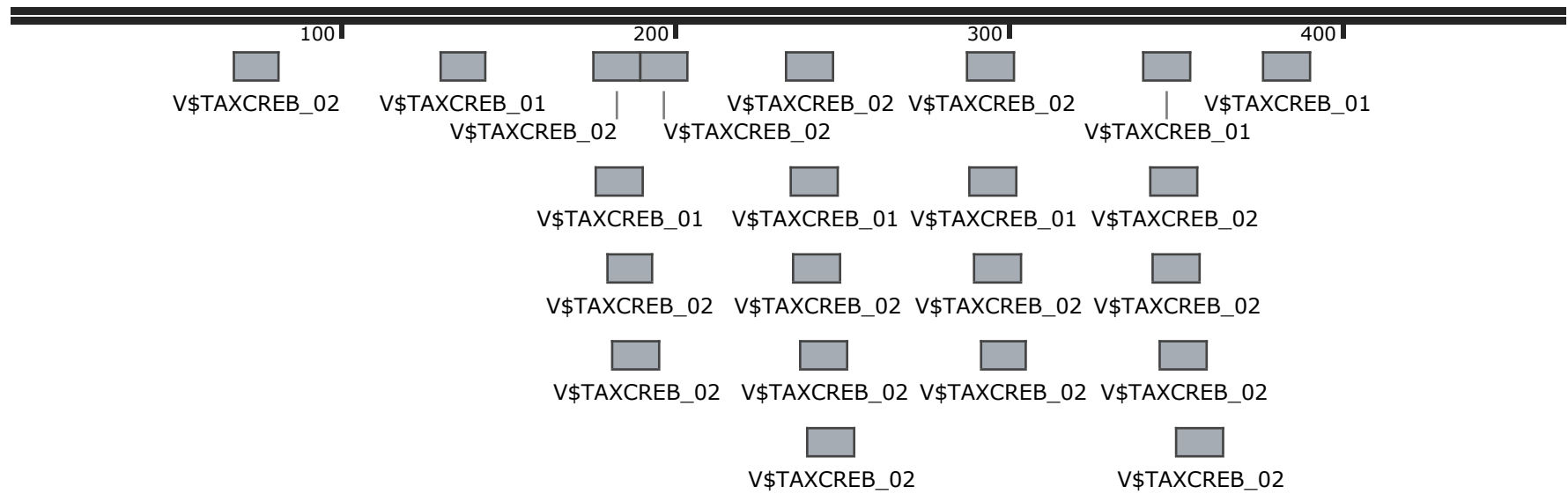

**AY530201.1**  
465 bp

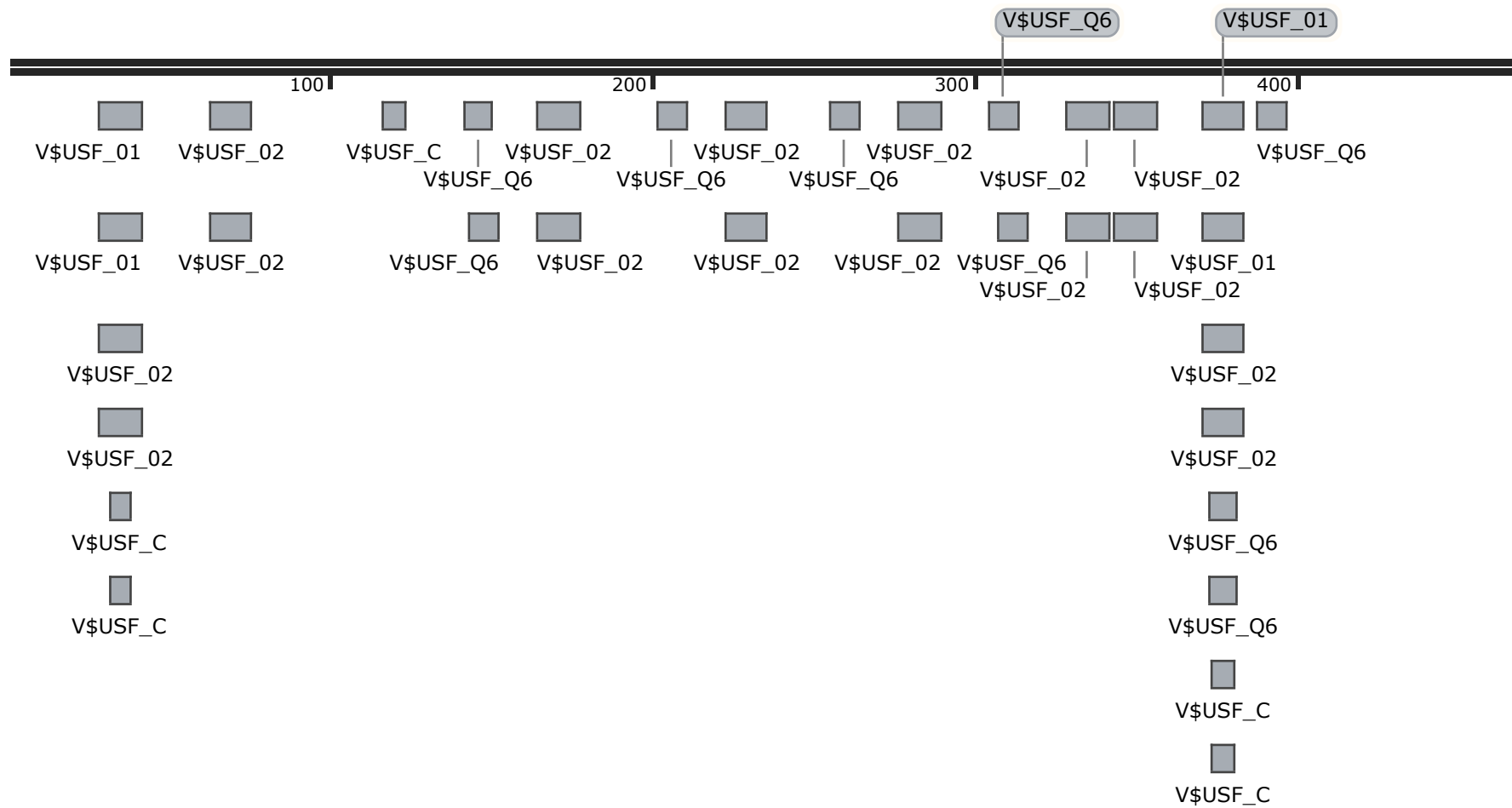

**AY530201.1**  
465 bp

Supplement: File S2 — Unabridged consensus transcription factor binding sites within the *4 allele of the hs1.2 enhancer. Allele *4 of the human hs1.2 enhancer is depicted as a detailed overview of the schema in . A general schematic of allele *4 is represented on page 1. A “core” (magenta) and a “tail” (light blue) sequence are common to all the hs1.2 allelic variants. The core sequence is also highly conserved in vertebrates. The “repeat” (yellow box) may be present from one to four times in human (see Figure 7 ) (alleles with more copies were found in primates). The allele *4 was analyzed in silico for potential transcription factor binding sites using TFBIND (tfbind.hgc.jp) and the results were depicted using SnapGene Viewer (https://www.snapgene.com). One transcription factor is reported on each page, starting from page 2. Codes refer to the sequence consensus motif in each transcription factor binding site, as listed in the “Gene Set Enrichment Analysis” database (GSEA - https://www.gsea-msigdb.org/gsea/msigdb/genesets.jsp). [file DataSheet_1.pdf]
